# Telomere-to-telomere genome of common bean (*Phaseolus vulgaris* L., YP4)

**DOI:** 10.1093/gigascience/giaf001

**Published:** 2025-05-14

**Authors:** Yan Wang, Xiaopeng Hao, Chunhai Chen, Haigang Wang, Peng Gao, Xukui Yang, Xue Dong, Huibin Qin, Meng Li, Sen Hou, Jianbo Jian, Jianwu Chang, Jing Wu, Zhixin Mu

**Affiliations:** Center for Agricultural Genetic Resources Research, Shanxi Agricultural University, Taiyuan 030031, China; Key Laboratory of Crop Gene Resources and Germplasm Enhancement on Loess Plateau, Ministry of Agriculture, Taiyuan 030031, China; Center for Agricultural Genetic Resources Research, Shanxi Agricultural University, Taiyuan 030031, China; Key Laboratory of Crop Gene Resources and Germplasm Enhancement on Loess Plateau, Ministry of Agriculture, Taiyuan 030031, China; BGI Genomics, Shenzhen 518083, China; Center for Agricultural Genetic Resources Research, Shanxi Agricultural University, Taiyuan 030031, China; Key Laboratory of Crop Gene Resources and Germplasm Enhancement on Loess Plateau, Ministry of Agriculture, Taiyuan 030031, China; BGI Genomics, Shenzhen 518083, China; BGI Genomics, Shenzhen 518083, China; Center for Agricultural Genetic Resources Research, Shanxi Agricultural University, Taiyuan 030031, China; Key Laboratory of Crop Gene Resources and Germplasm Enhancement on Loess Plateau, Ministry of Agriculture, Taiyuan 030031, China; Center for Agricultural Genetic Resources Research, Shanxi Agricultural University, Taiyuan 030031, China; Key Laboratory of Crop Gene Resources and Germplasm Enhancement on Loess Plateau, Ministry of Agriculture, Taiyuan 030031, China; Center for Agricultural Genetic Resources Research, Shanxi Agricultural University, Taiyuan 030031, China; Key Laboratory of Crop Gene Resources and Germplasm Enhancement on Loess Plateau, Ministry of Agriculture, Taiyuan 030031, China; Center for Agricultural Genetic Resources Research, Shanxi Agricultural University, Taiyuan 030031, China; Key Laboratory of Crop Gene Resources and Germplasm Enhancement on Loess Plateau, Ministry of Agriculture, Taiyuan 030031, China; BGI Genomics, Shenzhen 518083, China; Center for Agricultural Genetic Resources Research, Shanxi Agricultural University, Taiyuan 030031, China; Key Laboratory of Crop Gene Resources and Germplasm Enhancement on Loess Plateau, Ministry of Agriculture, Taiyuan 030031, China; Institute of Crop Sciences, Chinese Academy of Agricultural Sciences, Beijing 100089, China; Center for Agricultural Genetic Resources Research, Shanxi Agricultural University, Taiyuan 030031, China; Key Laboratory of Crop Gene Resources and Germplasm Enhancement on Loess Plateau, Ministry of Agriculture, Taiyuan 030031, China

**Keywords:** telomere-to-telomere (T2T), YP4, genome assembly, centromere

## Abstract

**Background:**

Common bean is a significant grain legume in human diets. However, the lack of a complete reference genome for common beans has hindered efforts to improve agronomic cultivars.

**Findings:**

Herein, we present the first telomere-to-telomere (T2T) genome assembly of common bean (*Phaseolus vulgaris* L., YP4) using PacBio High-Fidelity reads, ONT ultra-long sequencing, and Hi-C technologies. The assembly resulted in a genome size of 560.30 Mb with an N50 of 55.11 Mb, exhibiting high completeness and accuracy (BUSCO score: 99.5%, quality value (QV): 54.86). The sequences were anchored into 11 chromosomes, with 20 of 22 telomeres identified, leading to the formation of 9 T2T pseudomolecules. Furthermore, we identified repetitive elements accounting for 61.20% of the genome and predicted 29,925 protein-coding genes. Phylogenetic analysis suggested an estimated divergence time of approximately 11.6 million years ago between *P. vulgaris* and *Vigna angularis*. Comparative genome analysis revealed the expanded gene families and variations between YP4 and G19833 associated with defense response.

**Conclusions:**

The T2T reference genome and genomic insights presented here are crucial for future genetic studies not only in common bean but also in other legumes.

## Background

The common bean (*Phaseolus vulgaris* L., NCBI:txid3885; 2n=22) is an essential protein source that complements carbohydrate-rich foods such as rice, maize, and cassava [1]. It is globally significant as the most widely consumed legume, contributing substantially to daily caloric and protein intake, particularly in Africa and the Americas. In some regions, it accounts for up to 15% of total daily calories and 36% of daily protein intake [2]. Over 200 million people in sub-Saharan Africa rely on it as a staple food. Furthermore, the common bean is rich in health-beneficial nutrients, and their concentrations are heritable [3, 4]. Breeding programs aim to enhance these nutrient concentrations globally [5]. As a representative of the legume family, the common bean plays a vital role in global food security and offers significant potential for further nutritional enhancement through breeding efforts.

Extensive molecular genetics research has focused on the common bean. Common bean is organized in 2 geographically isolated and genetically differentiated wild gene pools: the Mesoamerican gene pool and the Andean gene pool [6]. In 2014, the genome of the Mesoamerican gene pool material (G19833) was decoded, revealing a scaffold length of 521.08 Mb with a contig N50 of 39,053 [2]. In 2016, the Andean gene pool (BAT93) was sequenced, yielding a genome size of 549.60 Mb and a contig N50 of 10,795 [7]. A 2020 study utilized 4.8 million single nucleotide polymorphims (SNPs) to conduct whole-genome association analysis on 20 agronomic traits, identifying over 500 genetic loci [8], providing precise markers for key traits in molecular breeding. Advancements in sequencing and assembly programs have resulted in a more contiguous common bean genome with a contig N50 size of 19.79 Mb [9]. Furthermore, a pan-genome study identified approximately 234 Mb of additional sequences containing 6,905 protein-coding genes [10]. Comparative genomic analysis revealed 376 nucleotide-binding site leucine-rich repeat (NLR) genes in common bean, compared to 319 NLR genes in soybean [11, 12]. This discrepancy may be attributed to the stronger adaptive capacity of common bean to ecological environments, leading to the evolution of more resistance mechanisms and thus more resistance genes [7]. Besides, numerous transcriptomic studies shed light on the genetic regulation and molecular mechanisms underlying various traits in this important crop, such as the GATA transcription factor, MADS-box gene family, and WOX gene family [13–15]. These highlight the significant interest in common beans and the importance of ongoing studies in this field.

**Figure 1:**
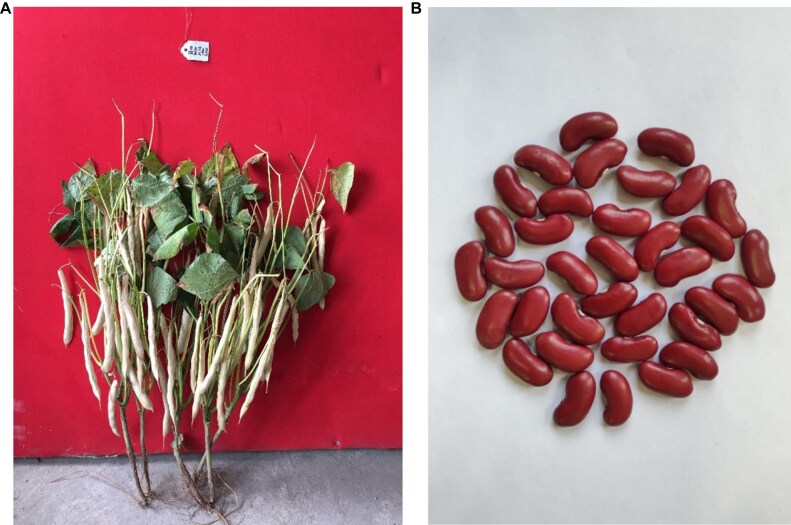
The YP4 plant sequenced in this study. (A) The plant of YP4. (B) The beans of YP4.

*De novo* genome assembly is a crucial tool in genomics research, but it has been hindered by assembly errors, large gaps, unplaced scaffolds, and strain-specific variants [16]. However, advances in sequencing and assembly algorithms have make telomere-to-telomere (T2T) genome assembly feasible, enabling comprehensive genome identification. Currently, over 63 T2T plant assemblies have been generated [17], including several essential crops, such as rice [18], maize [19], soybean [20], and sorghum [21]. Although the common bean holds great significance in agricultural and nutritional contexts, a T2T genome assembly for this important crop has not yet been reported. This study aims to bridge this gap by integrating Pacific Biosciences (PacBio) HiFi sequencing, Oxford Nanopore Technologies (ONT) ultra-long sequencing, and chromosomal conformational capture (Hi-C) technology to assemble a T2T genome of common bean variety Pinjinyun No. 4 (YP4). YP4 is a new variety of red kidney bean that was successfully bred by Shanxi Province in 2020. It has been officially named “Jinrenyun 202001” and represents a significant addition to the agricultural biodiversity of the region. It originates from British red variety seeds irradiated by cobalt-60 and belongs to the Andean center of cultivation, with a growth period of 99 days, tall stature, superior branching, and high stalk and seed yield (Fig. 1A). Its seeds are wide and plump, with a lustrous, vivid seed coat (Fig. 1B). Significantly, the average weight of 100 seeds is 51.4 g, and the seed contains 26.4% crude protein and 54.66% starch. This variety boasts a wide range of advantages and promising prospects, making it suitable for various applications, including grain consumption, processing, export, and a source of mature straw for feed. The deep sequencing of the YP4 whole genome holds significant value and importance for genetic research and molecular breeding development.

## Materials and Methods

### Sample collection

An individual plant of YP4 from Xiaodian District, Taiyuan, China (112.579°E,37.778°N), was selected for sequencing. Fresh leaves were harvested from this individual for genome DNA sequencing. Additionally, leaf, stem, root, flower, and pod samples were collected for RNA sequencing (RNA-seq) to facilitate gene annotation. All samples were promptly frozen in liquid nitrogen and stored at −80°C to ensure their preservation for further analysis.

### Sequencing and filtering

High-molecular-weight genomic DNA was extracted from the sample using a modified cetyltrimethylammonium bromide method [22] to facilitate subsequent library construction. For PacBio sequencing, libraries were prepared with an insert size of 15 kb using the SMRTbell Template Prep Kits from Pacific Biosciences. The sequencing was conducted in circular consensus sequencing mode on the PacBio Sequel II platform (RRID:SCR_017990). Subsequently, the subreads were processed using SMRTLink v8.0.0 [23] with the following parameters: “-minPasses 3 -minPredictedAccuracy 0.99 -minLength 500.”

For ONT sequencing, ONT ultra-long insert libraries were generated utilizing the Oxford Nanopore SQK-LSK109 kit and sequenced on the PromethION sequencer (RRID:SCR_017987). The ONT data underwent processing using NanoFilt v2.8.020 (RRID:SCR_016966) [24] with a quality threshold of 7.

In addition, Hi-C libraries based on *Dpn*II restriction enzymes were generated for Hi-C sequencing, following previously described methods [25]. These libraries were sequenced on the MGISEQ-2000 platform, generating paired-end 150-bp reads. Clean Hi-C data were obtained using SOAPnuke v2.0 (RRID:SCR_015025) [26] with parameters set as “-n 0.01 -l 20 -q 0.1 -i -Q 2 -G 2 -M 2 -A 0.5.”

For RNA-seq, libraries were constructed using the NEBNext Ultra RNA Library Prep Kit for Illumina (NEB) following the manufacturer’s protocol. The libraries were then sequenced on a MGISEQ-2000 instrument, producing 150-bp paired-end reads. Quality control of the RNA-seq data was performed using fastp v0.19.5 (RRID:SCR_016962) [27] with the following parameters: “–adapter_sequence AAGTCGGAGGCCAAGCGGTCTTAGGAAGACAA –adapter_sequence_r2 AAGTCGGATCGTAGCCATGTCGTTCTGTGAGCCAAGGAGTTG –average_qual 15 -l 150.”

### Genome assembly and Hi-C scaffolding

The *de novo* genome assembly of YP4 comprised 4 steps: primary assembly, Hi-C scaffolding, gap filling, and optimization. At first, the primary contigs were generated via Hifiasm v 0.15.1 (RRID:SCR_021069) [28] with the recommend command “hifiasm -o YP4.asm -t32 –ul ul.fq.gz –h1 read1.fq.gz –h2 read2.fq.gz HiFi-reads.fq.gz.” Subsequently, we used Bowtie2 v 2.2.9 (RRID:SCR_016368) [29] to align the Hi-C clean data to the primary contigs for anchoring contigs onto chromosomes. Low-quality reads were eliminated using the HiC-Pro pipeline (RRID:SCR_017643) [30] with default parameters. The remaining valid reads were utilized to anchor chromosomes with Juicer v 1.6 (RID:SCR_017226) [31] and 3D-DNA pipeline v 180419 (RRID:SCR_017227) [32]. Referring to the methods described in the gap-free genome of *Neosalanx taihuensis* [33], we applied the LR_Gapcloser (RRID:SCR_017021) [34] program to close gaps in the assembled chromosomes. To further enhance the genome quality, a polishing procedure described by Mc Cartney et al. [35] was implemented. Briefly, Winnowmap2 v 2.03 [36] was used to align the HiFi reads to the chromosomes, followed by filtering of alignments to exclude secondary alignments and those with excessive clipping using the “falconc bam-filter-clipped” tool. Finally, racon v 1.5.0 (RRID:SCR_017642) [37] was performed with the filtered alignments.

The completeness of the assembly was evaluated utilizing BUSCO v 5.5.0 (RRID:SCR_015008) [38] based on the embryophyta_odb10 database (1614 orthologs). The quality value was generated by Merqury program v 1.3 (RRID:SCR_022964) [39] with 17-mer.

### Genome annotations

We followed methods similar to those described by Qu et al. [40] for annotating repetitive sequences. Tandem Repeats Finder v 4.10 (RRID:SCR_022065) [41] was used to identify the tandem repeat elements. To detect interspersed repetitive sequences, we employed a strategy that combined *de novo* prediction and known repeat searching. RepeatModeler v 1.0.8 (RRID:SCR_015027) [42] and LTR_FINDER v 1.0.6 (RRID:SCR_015247) [43] were used to predict *de novo* repeat sequences. Subsequently, RepeatMasker v 4.0.7 (RRID:SCR_012954) [44] was applied to screen the YP4 genome against the combined *de novo* transposable element library. Additionally, RepeatMasker v 4.0.7 (RRID:SCR_012954) [44] and the Repbase database (RRID:SCR_021169) [45] were utilized to identify known transposable element repeats.

Similar to the method described for the wild blueberry T2T assembly [46], telomeric sequences and the centromeres region in the YP4 genome assembly were identified using quartet v 1.0.3 [47] with the “-c plant” option. The telomere repeat monomer identified by the TeloExplorer module in quarTeT program was “AAACCCT.” To identify the repeat unit, tandem repeats ranging from 30 to 500 bp with a copy number greater than 10 were scanned within the centromeric regions. The cd-hit v 4.8.1 (RRID:SCR_007105) [48] program was utilized to cluster the candidate repeats, and the representative sequences with the highest copy number were selected as the repeat unit.

The gene prediction process involved a comprehensive approach that integrated transcriptome-based, homology-based, and *ab initio* prediction methods. Initially, RNA-seq clean reads were assembled using Trinity v 2.15.1 (RRID:SCR_013048) [49] with parameters “–max_memory 200 G –CPU 40 –min_contig_length 200 –genome_guided_bam merged_sorted.bam –full_cleanup –min_kmer_cov 4 –min_glue 4 –bfly_opts ‘-V 5 –edge-thr=0.1 –stderr’ –genome_guided_max_intron 10000.” The resulting assembled transcripts were then aligned to the assembly utilizing Program to Assemble Spliced Alignment (PASA) v 2.4.1 (RRID:SCR_014656) [50]. Gene structures were generated from valid transcript alignments (PASA-set). Additionally, RNA-seq clean reads were mapped to the assembly via Hisat2 v 2.0.1 (RRID:SCR_015530) [51]. Subsequently, Stringtie v 1.2.2 (RRID:SCR_016323) [52] and TransDecoder v 5.7.1 (RRID:SCR_017647) were employed to assemble the transcripts and identify candidate coding regions, resulting in the creation of gene models (Stringtie-set). Homologous genomes from 6 assemblies, including *Glycine max* (Zhonghuang 13) [53], *Glycine max* (Wm82-NJAU) [54], *Arabidopsis thaliana* [55], *Phaseolus vulgaris* L. (G19833) [2], *Vigna angularis* (ensemble release-57), and *Medicago truncatula* (ensemble release-57) were downloaded and used as queries to search against the assembly using GeMoMa v 1.9 (RRID:SCR_017646) [56]. These homology predictions were referred to as “Homology-set.” For *ab initio* prediction methods, AUGUSTUS v 3.2.3 (RRID:SCR_008417) [57] was used to predict coding regions in the soft-masked genome. The gene models from these 3 sources were then merged using EvidenceModeler v 2.1.0 (RRID:SCR_014659) [58], with different weight parameters assigned to evidence from different sources (10 for PASA-set, 5 for Stringtie-set, 5 for Homology-set, and 1 for AUGUSTUS gene prediction). Finally, the generated gene models underwent further refinement with PASA v 2.4.1 (RRID:SCR_014656) [50] to obtain untranslated regions and alternative splicing variation information.

The integrated gene set was translated into amino acid sequences and annotated using the method described in Zhou et al. [33]. Furthermore, we employed the RGAugury pipeline [59] to screen the whole gene set for resistance gene analog (RGA) gene prediction with a method similar to that described in the eggplant genome study [60]. The default *P* value cutoff for initial RGA gene filtering was set to le-5 for BLASTP.

### Gene families and phylogenomic analysis

The OrthoMCL v2.0.9 (RRID:SCR_007839) [61] program, with default settings except for an inflation factor set at 1.5, was applied to determine gene families among 8 plants: *A. thaliana* [55], *Cicer arietinum* (GCF_000331145), *Cajanus cajan* (GCF_000340665.1), *G. max* [54], *M. truncatula* (ensembl release-57), *Lupinus angustifolius* (ensembl release-57), *P. vulgaris* (YP4, this study), and *V. angularis* (ensembl release-57). The input for OrthoMCL comprised the results of an all-versus-all BLASTP with an E-value cutoff of 1e-5. The outcomes of gene family clustering were summarized using UpSet (RRID:SCR_022731) [62]. A total of 1,296 single-copy gene families among these species were aligned using muscle v 5.1 [63] (RRID:SCR_011812). Subsequently, the alignments were concatenated into a super alignment matrix to reconstruct the phylogenetic tree via the maximum likelihood method using iqtree2 v 2.2.0 [64] with parameters of “-m MFP -B 1000.” The program MCMCtree v 4.4 in the PAML package (RRID:SCR_014932) [65] was used to estimate the divergence times among the 8 species, with the JC69 nucleotide substitution model and an independent rates clock. Two standard divergence time points from the TimeTree database (RRID: SCR_021162) [66] were used for calibration: (i) *A. thaliana–C. cajan* 102.0–112.5 million years ago (MYA) and (ii) *M. truncatula–C. arietinum* 24.9–51.0 MYA. CAFE v 4.2.1 (RRID:SCR_005983) [67] was used to measure the expansion and contraction of gene families. Based on the maximum likelihood modeling of gene gain and loss, we analyzed gene families for signs of expansion or contraction. Gene Ontology (GO) enrichment of YP4-specific genes, as well as genes in the expansion gene families, was conducted using clusterProfiler v4.2.2 (RRID:SCR_016884) [68].

### Comparative genomic analysis

We performed whole-genome alignment between YP4 and G19833 [2] using mummer v 4.0.0rc1 (RRID:SCR_018171) [69] with the following parameters: “–mum -g 1000 -c 90 -l 40.” The delta-filter program was used to identify alignment blocks with the setting “-1.” Subsequently, the show-snps program was utilized to detect SNPs and insertions/deletions (indels) with the settings “-Clr -x 1 -T.” SNPs and indels were annotated using the ANNOVAR package (RRID:SCR_012821) [70].

At the gene level, a pairwise synteny search was conducted using LAST v1270 (RRID:SCR_006119). The alignment results were refined using the JCVI utility libraries in MCScan (RRID:SCR_017650) (Python version) [71] with parameter “–cscore =0.99,” followed by visualization of the syntenic regions.

The method employed in the Sorghum T2T study [72] was used to investigate the YP4-specific genes. Initially, a sliding window strategy was applied to segment the YP4 genome, with a window size of 500 bp and a step of 100 bp. Subsequently, all segmented sequences were aligned to the G19833 genome using the BWA tool v 0.7.13-r1126 (RRID:SCR_010910) [73] with the MEM algorithm (-w 500 -M -t 16). Sequences that either failed to align with the G19833 genome or exhibited less than 25% coverage were classified as YP4-specific sequences. To determine YP4-specific genes, the longest coding sequence (CDS) for each gene was extracted. Genes with over 75% of their CDS covered by these specific sequences were designated as putative YP4-specific genes.

## Results

### Assembly of T2T common bean reference genome for YP4

**Figure 2:**
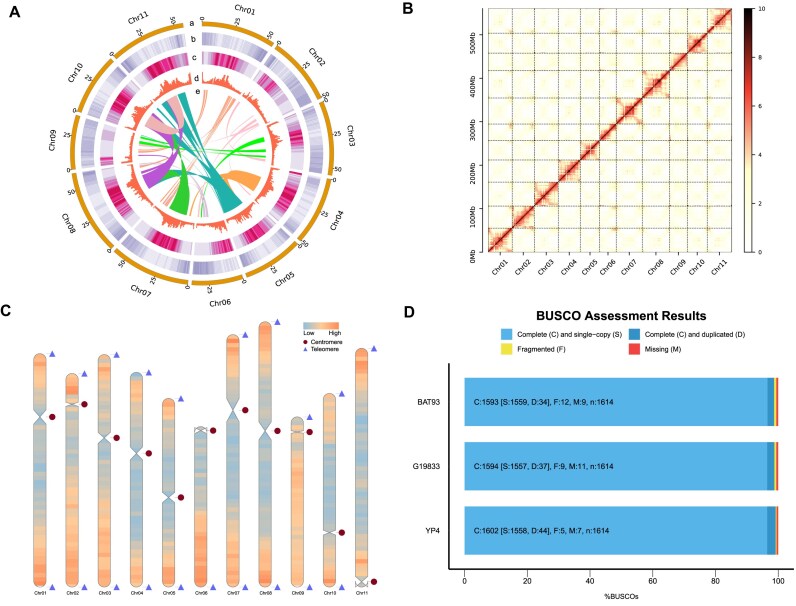
High-quality reference of YP4 genome. (A) Circos plot showing the characterization of the YP4 genome. From outside to inside: a, The length of pseudo-chromosome in the size of Mb. b, Gene density in 1-Mb sliding windows. c, Percentage of repetitive elements in 1-Mb sliding windows. d, GC content in nonoverlapping 1-Mb windows. e, Collinear regions within the YP4 assembly. (B) Heatmap displaying Hi-C interactions of YP4 pseudomolecules. Chr01–Chr11 are the abbreviations of 11 chromosomes. The abscissa and ordinate represent the order of each bin on the corresponding chromosome group. The color block illuminates the intensity of interaction from yellow (low) to red (high). (C) Telomere and centromere detection map. Triangles and circles represent telomeres and centromeres within the YP4 assembled chromosomes. The orange color represents regions with high gene density, while the sky blue color represents regions with low gene density. (D) BUSCO assessments of the YP4, G19833, and BAT93 genome.

The genome assembly of YP4 utilized multiple sequencing technologies, including PacBio HiFi reads, ONT ultra-long reads, and Hi-C reads. In summary, 31.75 Gb (∼56.67× coverage) of PacBio HiFi reads, 177.04 Gb of ONT ultra-long reads (∼315.97× coverage), and 144.79 Gb (∼258.42× coverage) of Hi-C data (Supplementary Table S1) were generated. The N50 length of the HiFi reads exceeded 15 kb, while the N50 length of the ONT reads was over 57 kb (Supplementary Table S1, Supplementary Fig. S1, Supplementary Fig. S2). The contigs were assembled using hifiasm, resulting in 558 contigs with a total size of 606.25 Mb and an N50 length of 32.18 Mb (Supplementary Table S2). Notably, the contig N50 of our assembly was significantly longer than that of the previously published genome versions, being 1.63-, 823.96-, and 2,980.83-fold longer than the Flavert (contig N50: 19.79 Mb), G19833 (contig N50: 39.05 kb), and BAT93 (contig N50: 10.80 kb) assemblies, respectively (Table 1, Supplementary Table S2). This substantial improvement establishes a solid foundation for the creation of a T2T genome assembly. Subsequently, the initial contigs served as the backbone to scaffold contigs into chromosomes with Hi-C data. Our result showed that the hifiasm assembly consisted of continuous sequences spanning the entire lengths of chromosomes 2 and 9, with 17 gaps distributed across 9 of the chromosomes (Supplementary Table S3). After gap filling and polishing, the final assembly achieved a total size of 560.30 Mb with an N50 of 55.11 Mb, comprising 11 gap-free chromosomes ranging from 38.04 to 62.89 Mb in length (Fig. 2A, Table 1).

**Table 1:** Comparison of 3 common bean assemblies

Assembly feature	YP4	G19833*	BAT93^†^	Flavert^#^
Size of assembly	560,297,700	521,076,696	549,748,340	615,703,893
Contig N50	55,110,595	39,053	10,795	19,791,875
Scaffold N50	55,110,595	50,367,376	39,037,607	54,932,568
Longest scaffold	62,894,056	59,662,532	50,710,336	63,359,058
Number of gaps	0	40,860	45,300	34
Number of protein-coding genes	29,925	28,134	30,491	29,549
Repetitive elements	61.20%	45.42%	35.50%	—
Quality value	54.86	—	—	—
Complete BUSCOs (*N* = 1,614)	99.50%	99.40%	99.40%	99.19%

* G19833 from Ensembl database (release-56).

† BAT93 from NCBI database under accession GCA_001517995.1.

# Flavert from NCBI database under accession number GCA_029448765.1.

To validate the accuracy and completeness of the YP4 T2T genome assembly, multiple approaches were employed. First, the Hi-C heatmap displayed a high level of consistency across all chromosomes, providing evidence for the accurate sequencing, ordering, and orientation of contigs in the YP4 genome assembly (Fig. 2B). Second, 100% of ONT reads and 99.95% of HiFi reads were effectively mapped to the YP4 genome assembly, resulting in genome coverage of 99.49% and 98.90%, respectively. Furthermore, the Merqury-estimated quality value of YP4 was 54.86, confirming the high accuracy of the assembly (Table 1). Third, all 11 centromeres were predicted in the YP4 genome assembly, with lengths ranging from 611,691 to 3,362,683 bp (Fig. 2C). Remarkably, 20 of the 22 telomeres were detected, leading to 9 T2T pseudomolecules for the entire genome (Fig. 2C, Supplementary Table S4). Finally, the BUSCO test indicated that the YP4 assembly successfully identified 99.5% of the 1,614 embryophyta gene set (Fig. 2D, Table 1). Overall, these findings demonstrate the high quality and reliability of the YP4 genome assembly.

### Annotation of repetitive elements and protein-coding genes

Approximately 342.40 Mb of the assembled YP4 genome was classified as repetitive sequences, constituting 61.20% of the genome (Supplementary Table S5). This proportion is higher than that in G19833 (45.42%) and BAT93 (35.50%; Table 1). Among the repetitive sequences, most were long terminal repeats (LTRs), which comprised 36.48% of the genome (Supplementary Table S6). The DNA, long interspersed nuclear element (LINE), and short interspersed nuclear element (SINE) classes accounted for 4.24%, 2.58%, and 0.11% of the genome, respectively (Supplementary Table S6).

**Figure 3:**
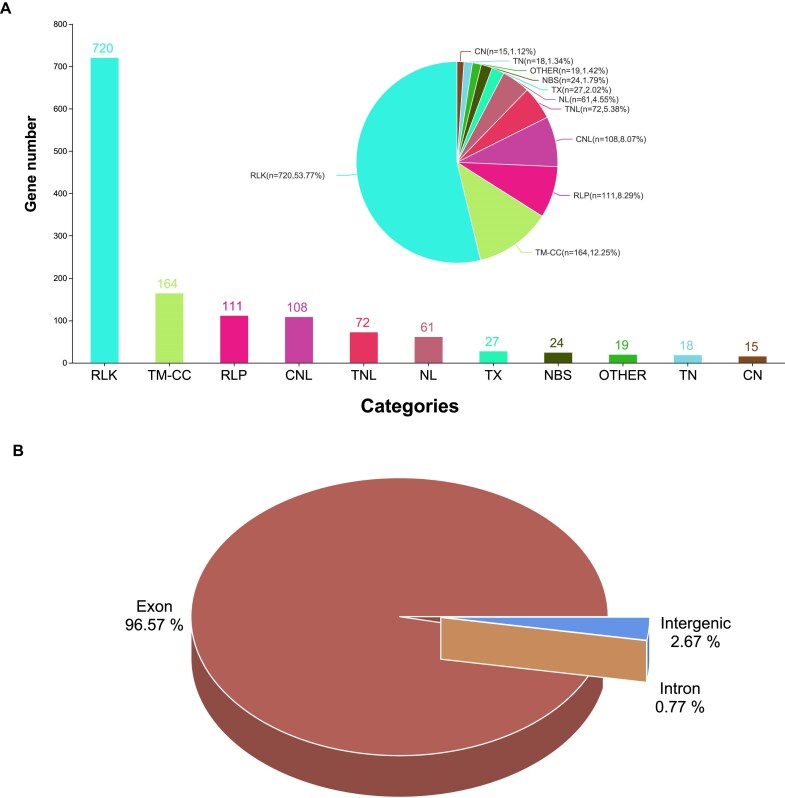
The gene annotation of the YP4 assembly. (A) Summary of RGA categories in the YP4 assembly. CC: coiled-coil; CN: CC-NBS; CNL: CC-NBS-LRR; NBS: nucleotide-binding site; NL: NBS-LRR; RLK: receptor-like kinase; RLP: receptor-like protein; TM: transmembrane; TN: TIR-NBS; TNL: TIR-NBS-LRR; TX: TIR-unknown domain. (B) RNA-seq clean data verified the accuracy of protein-coding gene prediction.

To facilitate genome annotation of the YP4 assembly, RNA sequencing was conducted on various tissues, including root, stem, leaf, flower, and pod, yielding a total of 118.68 Gb of clean reads (Supplementary Table S7). A combined prediction strategy identified 29,925 protein-coding genes, with mean lengths of 4,042 bp for the gene, 710 bp for the intron, and 1,241 bp for the coding sequence (Supplementary Table S8). The BUSCO assessment of the predicted gene sets showed 98.7% completeness with only 0.37% missing genes, indicating the robustness of the gene annotation (Supplementary Table S9). The length distribution of messenger RNA, coding sequences, exons, and introns among related species further supported the reliability of the annotation results (Supplementary Fig. S3). Of the predicted genes, 29,426 (98.33%) carried at least 1 conserved functional domain (Supplementary Table S10). Additionally, 1,339 RGAs were identified in the YP4 assembly, surpassing the 852 RGAs found in the BAT93 genome [7] (Fig. 3A). The largest category among the RGAs was receptor-like kinases (RLKs), comprising a total of 720 genes. Notably, 96.57% of the RNA-seq reads aligned to the predicted exons (Fig. 3B). Moreover, 23,006 (78.18%) of the genes exhibited a fragments per kilobase of transcript per million mapped reads (FPKM) value above 1.0 in at least 1 RNA-seq sample (Supplementary Fig. S4). These results confirm the completeness and accuracy of gene prediction across the YP4 genome.

### Detection of centromeres

The centromeric sequences of the 11 chromosomes were predicted, with an average length of 2.54 Mb. The longest centromeric sequence was 5.47 Mb on chromosome 7, while the shortest was 0.61 Mb on chromosome 9 (Table 2). The average repeat content in the centromeric sequences was 88.89%, significantly higher than the genome-wide repeat content of 61.20%. In most plants, centromere regions are characterized by a high abundance of retrotransposons and tandem repeats. In the case of YP4, tandem repeats comprised an average of 46.99% of the centromeric sequences, with LTR-Gypsy being the predominant interspersed repeat type (Table 2, Supplementary Table S11). A total of 400 genes overlapped with the centromeric regions, of which 377 had homologs in public databases. GO analysis indicated that these genes were significantly enriched in 7 terms, including “nucleic acid binding,” “sucrose transmembrane transporter activity,” “sucrose transport,” “DNA integration,” “zinc ion binding,” “plasma membrane,” and “endonuclease activity” (Supplementary Fig. S5). Notably, nucleic acid binding activity was significantly enriched among the rice centromeric genes [74], while DNA integration was significantly enriched among the grapevine centromeric genes [75]. Within the centromeric regions, 11 tandem repeat units were identified (Supplementary Table S12). Among them, 7 repeat units located on chromosome 1, chromosome 2, chromosome 4, chromosome 7, chromosome 8, chromosome 9, and chromosome 10 could be clustered together using cd-hit with a sequence identity threshold of 85%. To validate the authentic centromere locations, additional experiments such as fluorescence in situ hybridization and chromatin immunoprecipitation will be required.

**Table 2:** The characteristic of centromeres in YP4 assembly

Chromosomes	Start	End	Centro length	Repeat content (%)	Tandem repeats content (%)	LTR-Gypsy (%)	Gene number
Chr01	13,328,531	16,691,213	3,362,683	98.20	47.42	46.76	23
Chr02	6,817,424	7,789,605	972,182	89.28	69.75	9.22	28
Chr03	18,427,633	21,069,777	2,642,145	96.82	31.08	60.78	20
Chr04	17,673,363	20,755,122	3,081,760	95.35	38.33	48.26	22
Chr05	22,263,001	24,704,779	2,441,779	99.22	61.46	38.69	10
Chr06	227,300	1,666,213	1,438,914	66.82	37.00	19.35	39
Chr07	15,175,230	20,645,715	5,470,486	92.31	31.18	48.27	77
Chr08	23,748,882	28,008,021	4,259,140	93.89	30.97	53.06	55
Chr09	3,289,103	3,900,793	611,691	89.63	71.30	10.17	10
Chr10	32,420,713	33,554,620	1,133,908	88.27	40.52	30.39	25
Chr11	54,078,975	56,597,072	2,518,098	68.01	57.84	0.74	91

### Phylogenetic relationship analysis

**Figure 4:**
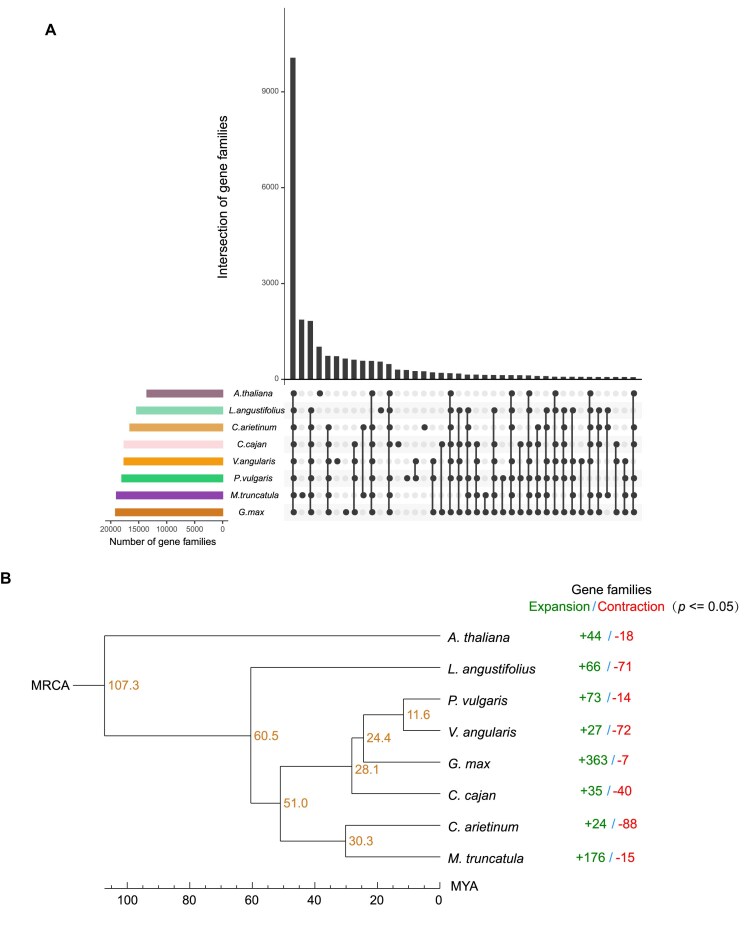
Evolution of the YP4 genome. (A) UpSetPlot representing the intersections of gene families among the 8 species. Rows and columns represent gene families and intersections, respectively. Black and gray circles indicate the existence or absence of a given intersection. The horizontal bar chart on the left side of the matrix indicates the size of gene family. (B) Phylogenetic tree of the 8 species. Numbers on nodes indicate the differentiation time. MRCA: most recent common ancestor.

The protein-coding genes of 7 plant species, including *A. thaliana, C. arietinum, C. cajan, G. max, M. truncatula, L.angustifolius*, and *V. angularis*, were clustered into 25,888 gene families together with the protein-coding genes of YP4 (Fig. 4A). Specifically, 294 gene families containing 1,755 genes were identified as specific to YP4 when compared with the other 7 plant species (Supplementary Table S13). Among these YP4-specific genes, 1,557 (88.72%) were supported by functional annotation (Supplementary Table S14), and they were significantly enriched in 23 GO terms. The top 10 most significantly enriched GO terms included “nucleic acid binding,” “zinc ion binding,” “inositol catabolic process,” “inositol oxygenase activity,” “nutrient reservoir activity,” “structural constituent of cell wall,” “manganese ion transmembrane transporter activity,” “cellular manganese ion homeostasis,” “response to auxin,” and “ribonuclease P complex” (Supplementary Fig. S6). A phylogenetic tree was constructed for the 8 plant species, with *A. thaliana* serving as an outgroup (Fig. 4B). The estimated divergence time between YP4 and *V. angularis* was around 11.6 MYA. Comparing with the most recent common ancestor (MRCA), YP4 showed 73 expansion events and 14 contraction events of each gene family (Fig. 4B). The expanded gene families of YP4 were mainly enriched in functions such as “ADP binding,” “defense response,” “signal transduction,” “terpene synthase activity,” “lyase activity,” “magnesium ion binding,” “manganese ion binding,” “hydrolase activity, hydrolyzing O-glycosyl compounds,” “phosphoric diester hydrolase activity,” “carbohydrate metabolic process,” and others (Supplementary Fig. S7).

### Comparison of YP4 and G19833 genomes

**Figure 5:**
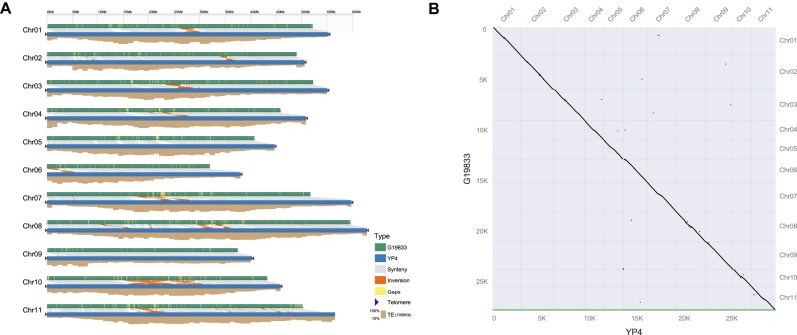
Genomic comparision between YP4 and G19833. (A) Collinearity between YP4 and G19833. Gray lines illustrate collinear regions between YP4 and G19833. Triangles denote the presence of telomere sequence repeats in YP4. The yellow bar indicates gap regions in G19833. (B) Dot-plot alignment between YP4 and G19833.

YP4 exhibited a longer assembly length compared to G19833, with 20 telomeres assembled in YP4 but none in G19833 (Table 1, Fig. 5A). Additionally, all 40,860 gaps present in the G19833 assembly were successfully filled in the YP4 assembly, achieving complete gap closure (Table 1, Fig. 5A). The JCVI analysis showed high collinearity between YP4 and G19833 (Fig. 5B). The syntenic regions contained 23,539 orthologous pairs, with 78.66% in YP4 and 83.43% in G19833. Given that the contig N50 of G19833 was only 39,053, indicating a lack of genomic continuity in the assembly, our focus was on the variations of SNPs and indels (2–50 bp) between YP4 and G19833. A total of 1,203,386 SNPs and 317,537 indels were detected between the 2 genomes (Supplementary Fig. S8). Among these variations, 44,734 (3.72%) SNPs and 3,126 (0.98%) indels were located in exonic regions (Supplementary Table S15, Supplementary Table S16). Specifically, there were 23,753 SNPs and 2,008 indels that potentially affected gene function, associated with 6,930 genes (Supplementary Table S17). GO enrichment analysis highlighted significant enrichments in 11 terms, including “ADP binding,” “defense response,” “ATP binding,” “protein kinase activity,” “protein phosphorylation,” “protein binding,” “protein serine/threonine kinase activity,” “sulfotransferase activity,” “oxidoreductase activity, acting on paired donors, with incorporation or reduction of molecular oxygen,” “monooxygenase activity,” and “recognition of pollen” (Supplementary Fig. S9). Furthermore, we identified 135 YP4-specific genes (Supplementary Table S18), which included 3 RGAs and 11 transcription factors. These YP4-specific genes were associated with various biological processes, such as “DNA binding,” “DNA repair,” “base-excision repair,” “regulation of RNA metabolic process,” and “positive regulation of DNA-binding transcription factor activity” (Supplementary Table S19). This indicated that YP4 possesses stronger resistance and a more intricate regulatory network compared to G19833. Notably, RNA-seq analysis revealed that 84 of these genes exhibited expression levels of FPKM ≥1, providing further evidence for their functional significance.

## Conclusions

The first T2T genome assembly of a typical common bean, YP4, was successfully accomplished using PacBio HiFi reads, ONT ultra-long sequencing, and Hi-C technologies. This assembly is notable for its exceptional completeness and accuracy. A total of 11 chromosomes were assembled, with 9 chromosomes meeting the telomere-to-telomere standard. Furthermore, the assembly predicted 342.40 Mb of repetitive sequences and identified 29,925 protein-coding genes. Evolutionary analysis suggests that investigating defense responses may be a promising avenue for understanding the genetic characteristics of common beans, further supported by comparative genomics analysis. Overall, this dataset provides a valuable resource for future genetic breeding research in common beans.

## Supplementary Material

giaf001_Supplemental_Files

giaf001_GIGA-D-24-00244_Original_Submission

giaf001_GIGA-D-24-00244_Revision_1

giaf001_Response_to_Reviewer_Comments_Original_Submission

giaf001_Reviewer_1_Report_Original_Submission Wei Fan -- 7/24/2024

giaf001_Reviewer_1_Report_Revision_1 Wei Fan -- 11/10/2024

giaf001_Reviewer_2_Report_Original_Submission Debasis Chattopadhyay -- 8/7/2024

## Data Availability

The genome assembly and all the sequencing data have been deposited in NCBI under the accession number BioProject PRJNA1072282. All additional supporting data are available in the *GigaScience* repository, GigaDB [76].

## Additional Files

**Supplementary Fig. S1**. The histogram illustrating the length distribution of PacBio HiFi reads.

**Supplementary Fig. S2**. The histogram illustrating the length distribution of ONT long reads.

**Supplementary Fig. S3**. Distribution of the gene components in the YP4 assembly. Window refers to the length of every point. No obvious unexpected differences exist among these 3 organisms, indicating the high quality of gene structure annotation.

**Supplementary Fig. S4**. The heatmap for log2(FPKM+1) values of 23,006 expressed genes. BS: branching stage; FL: flower; FS: flowering stage; LF: leaf; PD: pod; PFS: pod formation stage; RT: root; ST: stem.

**Supplementary Fig. S5**. GO enrichment analysis of genes within centromeric regions. Gene ratio (x-axis) is the percentage of the number of genes present in this GO term over the total number of genes in this category. Larger size of a GO term represents a higher gene number.

**Supplementary Fig. S6**. GO enrichment analysis of YP4-specific genes. Gene ratio (x-axis) is the percentage of the number of genes present in this GO term over the total number of genes in this category. Larger size of a GO term represents a higher gene number.

**Supplementary Fig. S7**. GO enrichment analysis of YP4-expanded genes. Gene ratio (x-axis) is the percentage of the number of genes present in this GO term over the total number of genes in this category. Larger size of a GO term represents a higher gene number.

**Supplementary Fig. S8**. Genomic variations between YP4 and G19833. (A) The density plot of SNPs between YP4 and G19833. (B) The density plot of indels between YP4 and G19833. Window size is 1 Mb, and the color depth of each window represents the range of mutations.

**Supplementary Fig. S9**. GO enrichment analysis of 6,930 genes affected by variations between YP4 and G19833. Gene ratio (x-axis) is the percentage of the number of genes present in this GO term over the total number of genes in this category. Larger size of a GO term represents a higher gene number.

**Supplementary Table S1**. Summary of whole-genome sequencing data of the YP4 genome.

**Supplementary Table S2**. The statistics of the hifiasm assembly.

**Supplementary Table S3**. The statistics of the anchored chromosome length.

**Supplementary Table S4**. The identified telomeres in the YP4 assembly.

**Supplementary Table S5**. General statistics of repeats in the YP4 assembly.

**Supplementary Table S6**. The summary of interspersed repeat contents in YP4 assembly.

**Supplementary Table S7**. The summary of RNA-seq sequencing quality.

**Supplementary Table S8**. Summary of gene structure prediction in the YP4 genome.

**Supplementary Table S9**. BUSCO analysis of YP4 gene set completeness.

**Supplementary Table S10**. Number of functional annotations for predicted genes in the YP4 assembly.

**Supplementary Table S11**. Statistics on the main types of interspersed repeats within the centromere.

**Supplementary Table S12**. Tandem repeat unit sequence within the centromere.

**Supplementary Table S13**. Gene families in YP4 and other species.

**Supplementary Table S14**. The list of YP4-specific genes.

**Supplementary Table S15**. The annotation of SNPs between YP4 and G19833.

**Supplementary Table S16**. The annotation of indels between YP4 and G19833.

**Supplementary Table S17**. The gene list impacted by variation between YP4 and G19833.

**Supplementary Table S18**. The YP4-specific gene list.

**Supplementary Table S19**. GO enrichment of the YP4-specific gene.

## Abbreviations

BLAST: Basic Local Alignment Search Tool; BUSCO: Benchmarking Universal Single-Copy Orthologs; Gb: gigabase pairs; GO: Gene Ontology; Hi-C: high-throughput chromosome conformation capture; HiFi: high-fidelity; KEGG: Kyoto Encyclopedia of Genes and Genomes; indels: insertions/deletions; LINE: long interspersed nuclear element; LTR: long terminal repeat; Mb: megabase pairs; MRCA: most recent common ancestor; MYA: million years ago; PASA: Program to Assemble Spliced Alignments; RGAs: resistance gene analogs; RNA-seq: RNA sequencing; SMRT: single-molecule real-time sequencing; SNPs: single nucleotide polymorphisms; T2T: telomere-to-telomere.
