# Supplementary material for: Telomere-to-telomere genome of common bean (Phaseolus vulgaris L., YP4)
Source: Gigascience. 2025 May 14;14:giaf001. doi: 10.1093/gigascience/giaf001 (PMC12077395; doi:10.1093/gigascience/giaf001)
Supplement: giaf001_Supplemental_Files [file giaf001_supplemental_files.zip › Supplementary Figure1-9.docx]

**Supplementary Figures**


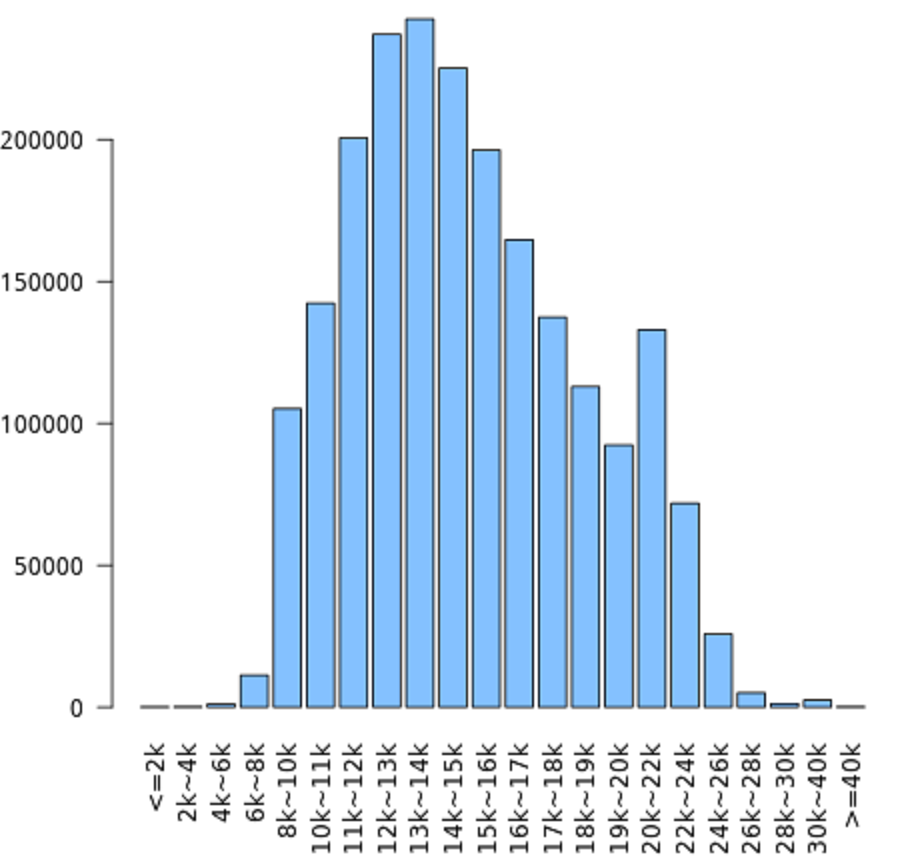


**Supplementary Fig. 1 The histogram illustrating the length distribution of PacBio HiFi reads.**


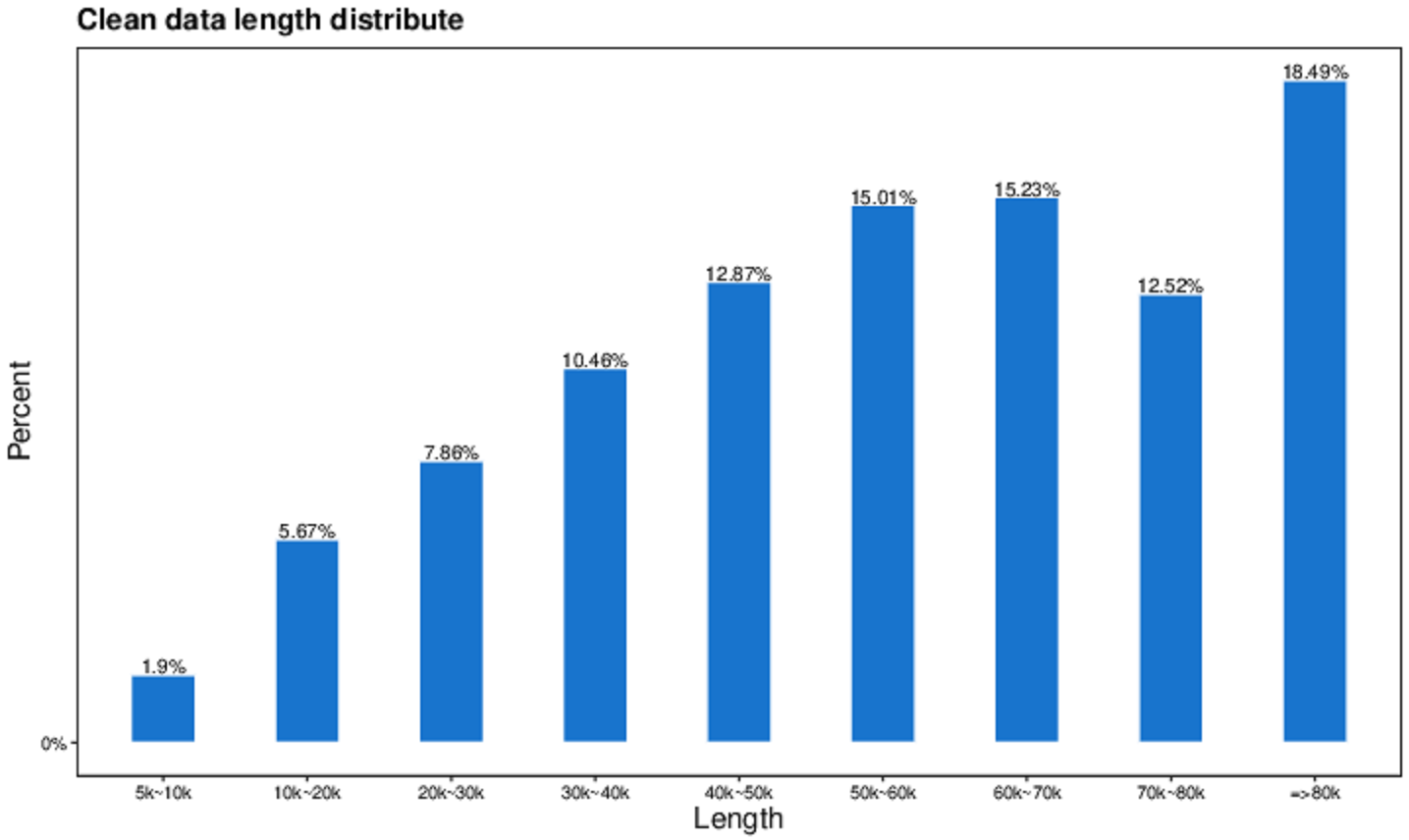


**Supplementary Fig. 2 The histogram illustrating the length distribution of ONT long reads.**


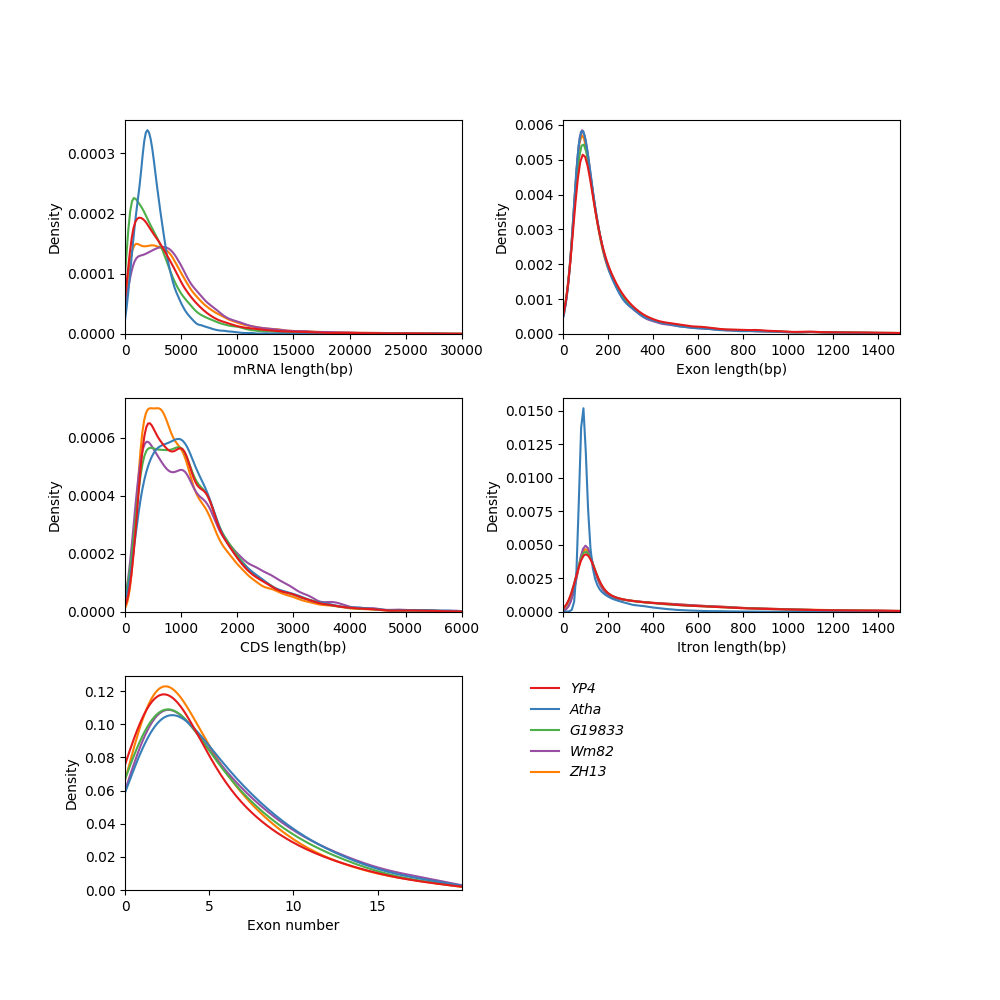
**Supplementary Fig. 3 Distribution of the gene components in the YP4 assembly.** Window refers to the length of every point. No obvious unexpected differences exist among these three organisms, indicating the high quality of gene structure annotation.


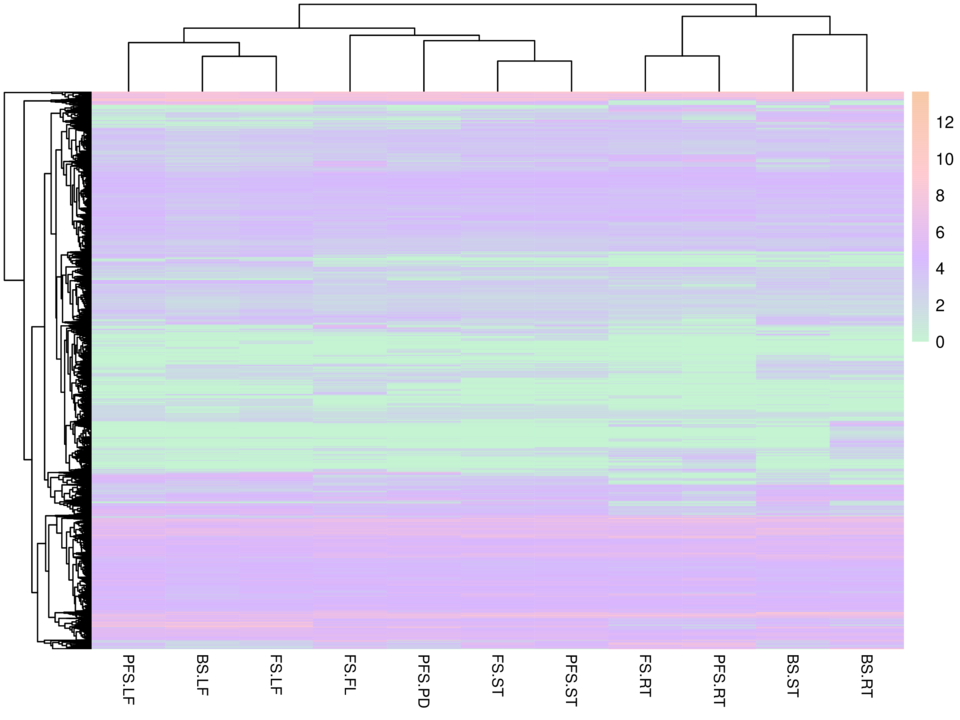


**Supplementary Fig. 4 The heatmap for log2(FPKM+1) values of 23,006 expressed genes.** BS：Branching Stage; FS: Flowering Stage; PFS: Pod Formation Stage; LF: Leaf; ST: Stem; RT: Root; FL: Flower; PD: Pod.


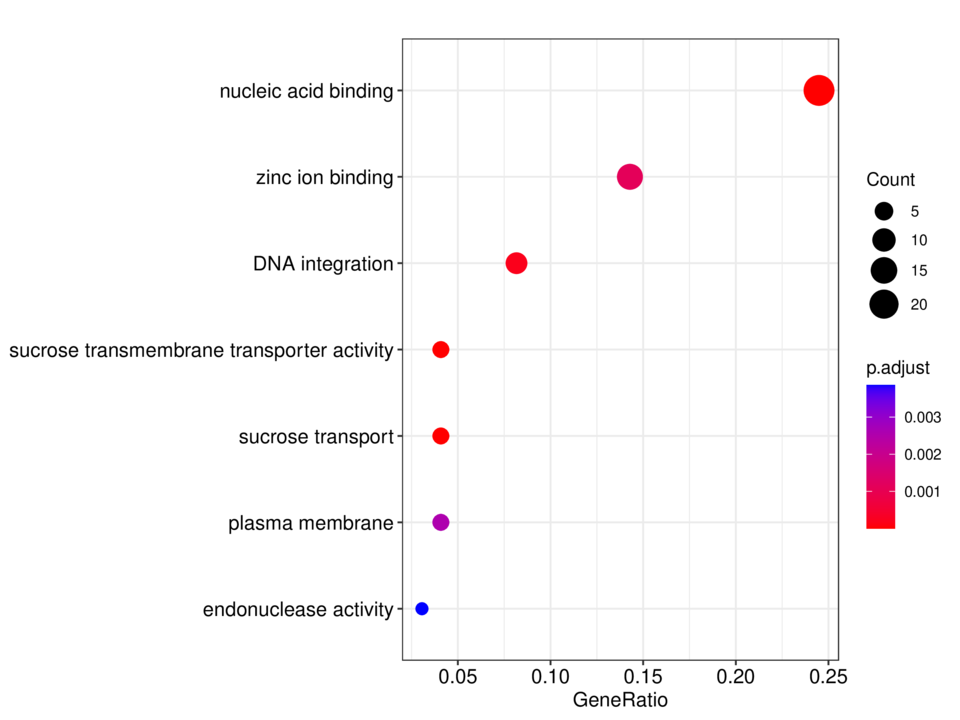


**Supplementary Fig. 5 GO enrichment analysis of genes within centromeric regions.** Gene ratio (x-axis) is the percentage of the number of genes present in this GO term over the total number of genes in this category. Larger size of a GO term represents a higher gene number.


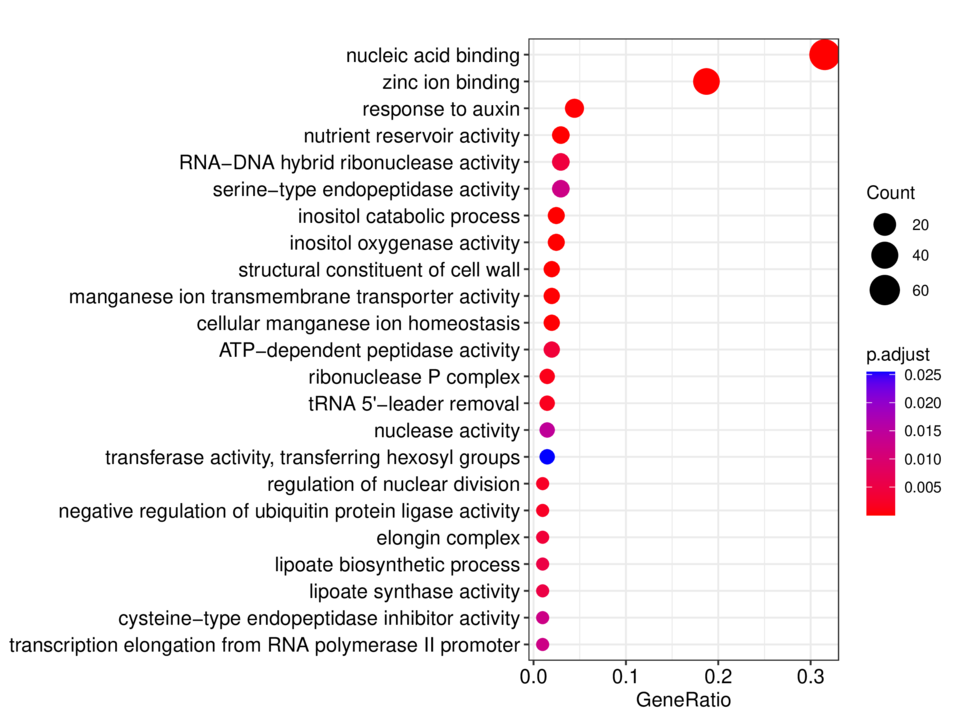


**Supplementary Fig. 6 GO enrichment analysis of YP4 specific genes.** Gene ratio (x-axis) is the percentage of the number of genes present in this GO term over the total number of genes in this category. Larger size of a GO term represents a higher gene number.


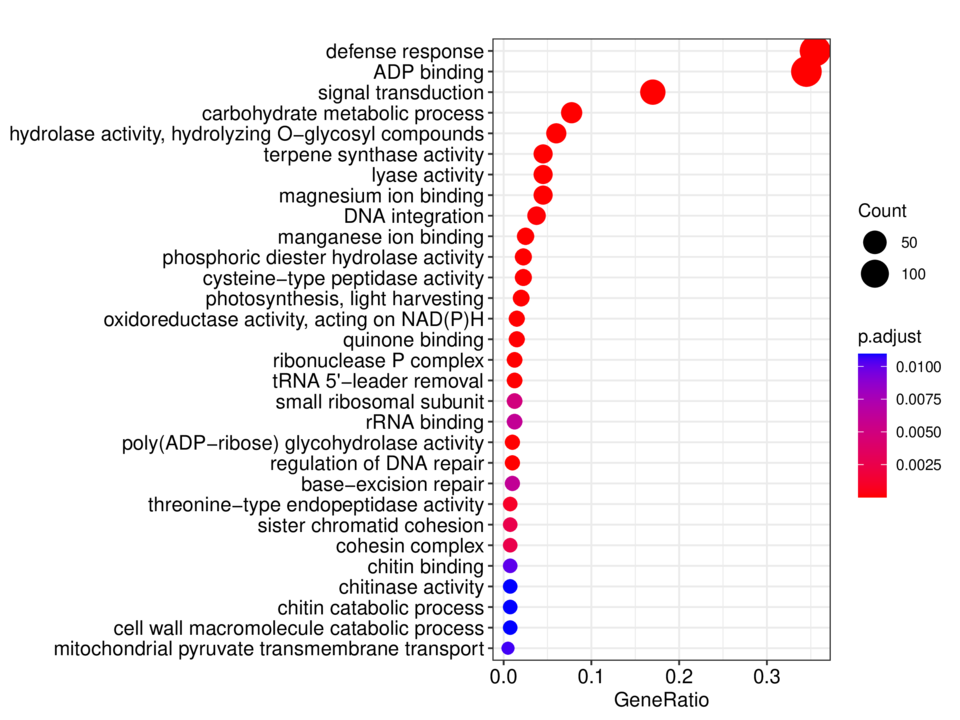


**Supplementary Fig. 7 GO enrichment analysis of YP4 expanded genes.** Gene ratio (x-axis) is the percentage of the number of genes present in this GO term over the total number of genes in this category. Larger size of a GO term represents a higher gene number.


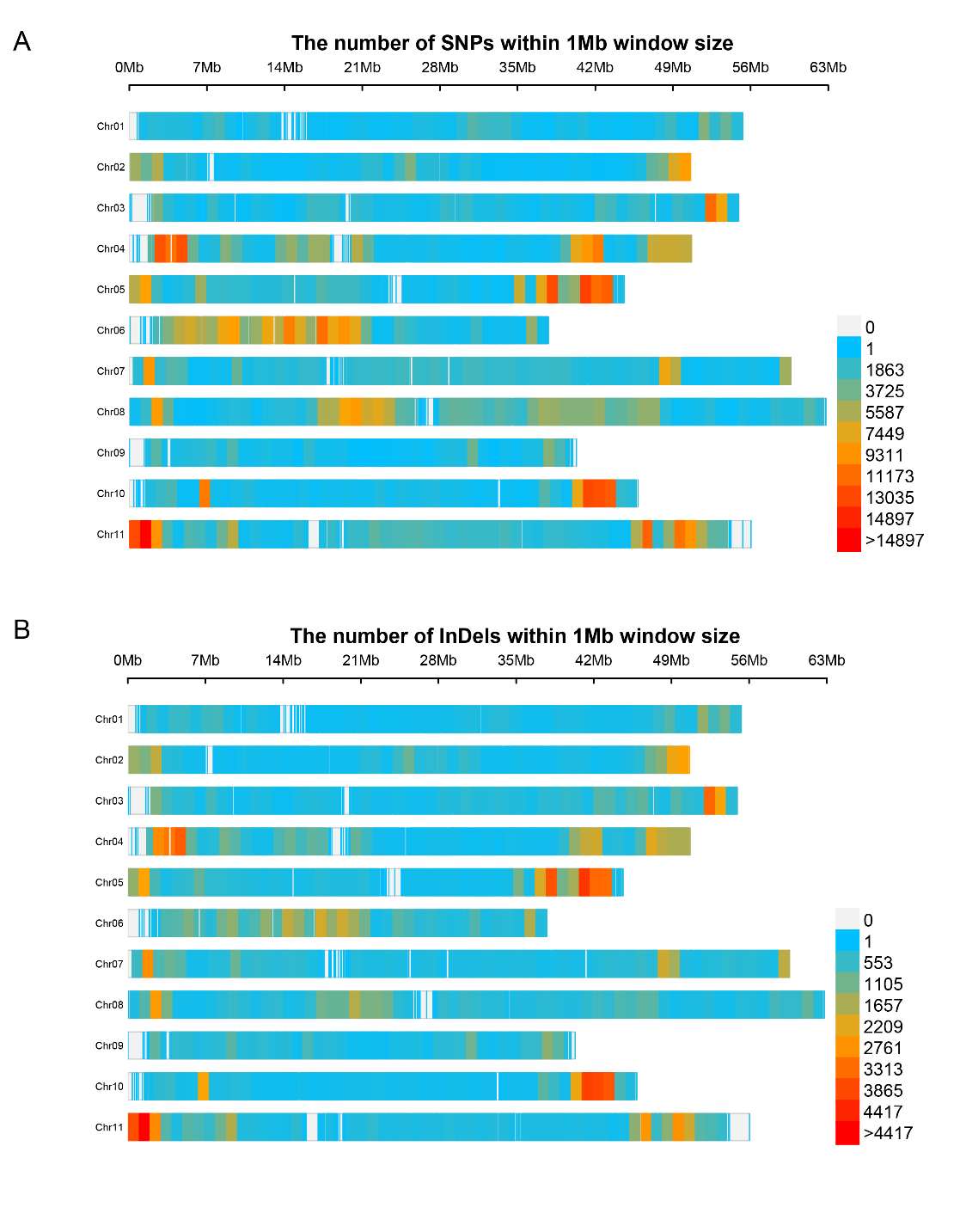


**Supplementary Fig. 8 Genomic variations between YP4 and G19833. A**, The density plot of SNPs between YP4 and G19833. **B**, The density plot of Indels between YP4 and G19833. Window size is 1 Mb, and the color depth of each window represents the range of mutations.


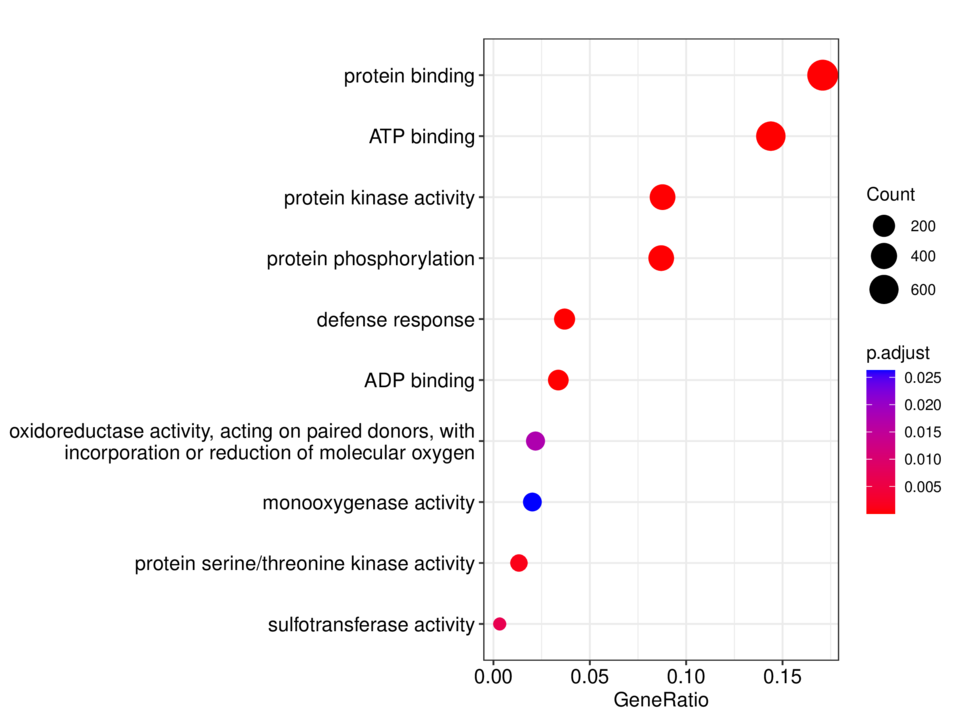


**Supplementary Fig. 9 GO enrichment analysis of 6,930 genes affected by variations between YP4 and G19833.** Gene ratio (x-axis) is the percentage of the number of genes present in this GO term over the total number of genes in this category. Larger size of a GO term represents a higher gene number.
