# Supplementary material for: Telomere-to-telomere genome of common bean (Phaseolus vulgaris L., YP4)
Source: Gigascience. 2025 May 14;14:giaf001. doi: 10.1093/gigascience/giaf001 (PMC12077395; doi:10.1093/gigascience/giaf001)
Supplement: giaf001_GIGA-D-24-00244_Original_Submission [file giaf001_giga-d-24-00244_original_submission.pdf]

GigaScience

Telomere-to-telomere gap-free genome of common bean (Phaseolus vulgaris L., YP4)

--Manuscript Draft--

**Manuscript Number:**	GIGA-D-24-00244
**Full Title:**	Telomere-to-telomere gap-free genome of common bean (Phaseolus vulgaris L., YP4)
**Article Type:**	Research
**Funding Information:**	National Natural Science Foundation of China (32241041) — Mr. Jing Wu<br>The Major Special Science and Technology Projects in Shanxi Province (202101140601027) — Mr. Haigang Wang<br>China Agriculture Research System of MOF and MARA-Food Legumes (CARS-08) — Mr. Xiaopeng Hao<br>The National Key Research and Development Program of China (2021YFD1600600) — Mr. Jianwu Chang<br>Biological Breeding Engineering of Shanxi Agricultural University (YZGC148) — Mr. Xiaopeng Hao

Abstract:

Background

Common bean is a significant grain legume in human diets. However, the lack of a complete reference genome for common beans has hindered efforts to enhance agronomic cultivars.

Findings

Herein, we report the first telomere-to-telomere gap-free genome assembly of common bean (Phaseolus vulgaris L., YP4) using PacBio High-Fidelity reads, ONT ultra-long sequencing, and Hi-C technologies. The assembly yielded a genome size of 560.30 Mb with an N50 of 55.11 Mb, exhibiting high completeness and accuracy (BUSCO score: 99.5%, QV: 54.86). The sequences were anchored into eleven chromosomes, with 20 out of 22 telomeres identified, resulting in nine T2T pseudomolecules. Furthermore, we identified repetitive elements accounting for 61.20% of the genome and predicted 29,925 protein-coding genes. Phylogenetic analysis suggested an estimated divergence time of approximately 11.6 MYA between P. vulgaris and V. angularis. Comparative genome analysis found that the expanded gene families and variations between YP4 and G19833 related to defense response.

Conclusions

The telomere-to-telomere gap-free reference genome and genomic insights presented here are crucial for future genetic studies not only in common bean but also in other legumes.

**Corresponding Author:**	yan wang<br>Shanxi Agricultural University<br>Taiyuan, CHINA
**Corresponding Author Secondary Information:**	
**Corresponding Author's Institution:**	Shanxi Agricultural University
**Corresponding Author's Secondary Institution:**	
**First Author:**	yan wang
**First Author Secondary Information:**	

Powered by Editorial Manager® and ProduXion Manager® from Aries Systems Corporation

Order of Authors:	yan wang
	Xiaopeng Hao
	Chunhai Chen
	Haigang Wang
	Peng Gao
	Xukui Yang
	Xue Dong
	Huibin Qin
	Meng Li
	Sen Hou
	Jianbo Jian
	Jianwu Chang
	Jing Wu
	Zhixin Mu
Order of Authors Secondary Information:	
Additional Information:	
Question	Response
Are you submitting this manuscript to a special series or article collection?	No
**Experimental design and statistics**<br><br>Full details of the experimental design and statistical methods used should be given in the Methods section, as detailed in our Minimum Standards Reporting Checklist. Information essential to interpreting the data presented should be made available in the figure legends.<br><br>Have you included all the information requested in your manuscript?	Yes
**Resources**<br><br>A description of all resources used, including antibodies, cell lines, animals and software tools, with enough information to allow them to be uniquely identified, should be included in the Methods section. Authors are strongly encouraged to cite Research Resource Identifiers (RRIDs) for antibodies, model organisms and tools, where possible.	Yes

Powered by Editorial Manager® and ProduXion Manager® from Aries Systems Corporation

Have you included the information requested as detailed in our Minimum Standards Reporting Checklist?	
**Availability of data and materials**<br><br>All datasets and code on which the conclusions of the paper rely must be either included in your submission or deposited in publicly available repositories (where available and ethically appropriate), referencing such data using a unique identifier in the references and in the “Availability of Data and Materials” section of your manuscript.<br><br>Have you have met the above requirement as detailed in our Minimum Standards Reporting Checklist?	Yes

Powered by Editorial Manager® and ProduXion Manager® from Aries Systems Corporation

Manuscript
Click here to access/download;Manuscript;Manuscript.docx

DATANOTE

Telomere-to-telomere gap-free genome of common bean (*Phaseolus vulgaris* L., YP4)

Yan Wang[1,2†], Xiaopeng Hao[1,2,†], Chunhai Chen[3,†], Haigang Wang[1,2,†], Peng Gao[3,†], Xukui Yang[3,†], Xue Dong[1,2], Huibin Qin[1,2], Meng Li[1,2], Sen Hou[1,2], Jianbo Jian[3], Jianwu Chang[1,2,*], Jing Wu[4,*], Zhixin Mu[1,2,*]

[1]Center for Agricultural Genetic Resources Research, Shanxi Agricultural University, Taiyuan 030031, China

[2]Key Laboratory of Crop Gene Resources and Germplasm Enhancement on Loess Plateau, Ministry of Agriculture, Taiyuan 030031, China

[3]BGI Genomics, Shenzhen 518083, China

[4]Institute of Crop Sciences, Chinese Academy of Agricultural Sciences, Beijing 100089, China

[*]Correspondence address. Zhixin Mu, Center for Agricultural Genetic Resources Research, Shanxi Agricultural University, NO.161 Longcheng North Street, Xiaodian District Taiyuan, Shanxi Province, China. E-mail: muzx2008@sina.com; Jing Wu, Institute of Crop Sciences, Chinese Academy of Agricultural Sciences, NO.12 Zhongguancun South Street, Haidian District Beijing, China. E-mail: wujing@caas.cn; Jianwu Chang, Center for Agricultural Genetic Resources Research, Shanxi Agricultural University, NO. 161 Longcheng North Street, Xiaodian District Taiyuan, Shanxi Province, China. E-mail: changjianw2005@163.com

[†] These authors contributed equally to this work.

Abstract

Background

Common bean is a significant grain legume in human diets. However, the lack of a complete reference genome for common beans has hindered efforts to enhance agronomic cultivars.

Findings

Herein, we report the first telomere-to-telomere gap-free genome assembly of common bean (*Phaseolus vulgaris* L., YP4) using PacBio High-Fidelity reads, ONT ultra-long sequencing, and Hi-C technologies. The assembly yielded a genome size of 560.30 Mb with an N50 of 55.11 Mb, exhibiting high completeness and accuracy (BUSCO score: 99.5%, QV: 54.86). The sequences were anchored into eleven chromosomes, with 20 out of 22 telomeres identified, resulting in nine T2T pseudomolecules. Furthermore, we identified repetitive elements accounting for 61.20% of the genome and predicted 29,925 protein-coding genes. Phylogenetic analysis suggested an estimated divergence time of approximately 11.6 MYA between *P. vulgaris* and *V. angularis*. Comparative genome analysis found that the expanded gene families and variations between YP4 and G19833 related to defense response.

Conclusions

The telomere-to-telomere gap-free reference genome and genomic insights presented here are crucial for future genetic studies not only in common bean but also in other legumes.

Background

The common bean (*Phaseolus vulgaris* L., 2n=22) is an essential protein source that complements carbohydrate-rich foods such as rice, maize, and cassava [1]. It holds global

significance as the most consumed legume, substantially contributing to daily caloric and protein intake, particularly in Africa and the Americas. In some regions, it constitutes up to 15% of total daily calories and 36% of daily protein [2]. Over 200 million people in sub-Saharan Africa depend on it as a staple. Furthermore, the common bean is rich in nutrients that benefit health, and their concentrations are heritable [3, 4]. Breeding programs aim to enhance these nutrient concentrations globally [5]. In conclusion, as a representative of the legume family, the common bean plays a vital role in global food security and provides immense potential for further nutritional enhancement through breeding efforts.

Extensive molecular genetics research has focused on the common bean. Common bean is organized in two geographically isolated and genetically differetiated wild gene pools: the Mesoamerican gene pool and the Andean gene pool [6]. In 2014, the genome of the Mesoamerican gene pool material (G19833) was decoded, revealing a scaffold length of 521.08 Mb with a contig N50 of 39,053 [2]. Subsequently, in 2016, researchers performed genome *de novo* sequencing of the Andean gene pool material (BAT93), yielding a sequence of 549.60 Mb, with a contig N50 of 10,795 [7]. A 2020 study utilized 4.8 million SNPs to conduct whole-genome association analysis on 20 agronomic traits, identifying over 500 genetic loci [8]. This discovery provided precise markers for key traits in molecular breeding. Comparative genomic analysis unveiled the identification of 376 nucleotide-binding site-leucine-rich repeat (NLR) genes in common bean, compared to 319 NLR genes in soybean [9, 10]. This discrepancy may be attributed to the stronger adaptive capacity of common bean to ecological environments, leading to the evolution of more resistance mechanisms and thus more resistance genes [7]. Besides, numerous transcriptomic studies shed light on the genetic regulation and molecular

mechanisms underlying various traits in this important crop, such as the investigation of GATA transcription factor, MADS-box gene family, and WOX gene family [11-13]. These highlight the significant interest in common beans and the importance of ongoing studies in this field.

De novo genome assembly is a crucial tool in genomics research, but it has been hindered by assembly errors, large gaps, unplaced scaffolds, and strain-specific variants. [14]. Advances in sequencing and assembly algorithms now make telomere-to-telomere (T2T) gap-free genome assembly feasible, enabling comprehensive genome identification. Currently, over 63 T2T plant assemblies have been generated [15], including several essential crops, such as rice [16], maize [17], soybean [18], and sorghum [19]. Although the common bean holds great significance in agricultural and nutritional contexts, a T2T gap-free genome assembly for this important crop has not been reported to date. In this study, we aim to bridge this gap by integrating Pacific Biosciences (PacBio) HiFi sequencing, Oxford Nanopore Technologies (ONT) ultra-long sequencing, and chromosomal conformational capture (Hi-C) technology to assemble a T2T gap-free genome of common bean variety Pinjinyun No. 4 (YP4). YP4 is a novel red kidney bean identified by Shanxi Province in 2021 and designated as Jinrenyun 202001. It originating from British red variety seeds irradiated by cobalt-60, belongs to the Andean center of cultivation, with a growth period of 99 days, tall stature, superior branching, and high stalk yield (Fig. 1A). Its seeds are wide, plump, with a lustrous, vivid seed coat (Fig. 1B). Notably, the average 100-seed weight is 51.4 grams, with crude protein and starch contents of 26.4% and 54.66%, respectively. This variety has wider advantages and prospects whether it is used for grain consumption, processing, exportation, or as mature straw feed. The deep sequencing of the whole genome of

YP4 holds significant value and importance for genetic research and molecular breeding development.

Materials and methods

Sample collection

An individual plant of YP4 from Xiaodian district, Taiyuan, China (112.579° E, 23 37.778° N), was selected for sequencing. Fresh leaves harvested from this individual for genome DNA sequencing. Additionally, leaf, stem, root, flower, and pod samples were collected for RNA-sequencing (RNA-seq) to facilitate gene annotation. All samples were promptly frozen in liquid nitrogen and stored at -80°C to ensure their preservation for further analysis.

Sequencing and filtering

High-molecular-weight genomic DNA was extracted from the sample using a modified cetyltrimethylammonium bromide method [20] to facilitate subsequent library construction. For PacBio sequencing, libraries were prepared with an insert size of 15 kb using the SMRTbell Template Prep Kits from Pacific Biosciences of California, Inc. The sequencing was conducted in circular consensus sequencing mode on the PacBio Sequel II platform (RRID:SCR_017990). Subsequently, the subreads were processed using SMRTLink v8.0.0 [21] with the following parameters: "-minPasses 3 -minPredictedAccuracy 0.99 -minLength 500".

For ONT sequencing, ONT ultra-long insert libraries were generated using the Oxford Nanopore SQK-LSK109 kit and sequenced on the PromethION sequencer (RRID:SCR_017987).

The ONT data underwent processing using NanoFilt v2.8.020 (RRID:SCR_016966) [22] with a quality threshold of 7.

In addition, Hi-C libraries based on *Dpn*II restriction enzymes were generated for Hi-C sequencing, as previously described [23]. These libraries were sequenced on the MGISEQ-2000 platform, generating paired-end 150 bp reads. Clean Hi-C data were obtained using SOAPnuke v2.0 (RRID:SCR_015025) [24] with parameters set as "-n 0.01 -l 20 -q 0.1 -i -Q 2 -G 2 -M 2 -A 0.5".

For RNA-seq, libraries were constructed using the NEBNext® Ultra™ RNA Library Prep Kit for Illumina® (NEB, Ipswich, MA, USA) following the manufacturer's protocol. The libraries were then sequenced on a MGISEQ-2000 instrument, producing 150 bp paired-end reads. Quality control of the RNA-seq data was performed using fastp v0.19.5 (RRID:SCR_016962) [25] with the following parameters: "--adapter_sequence AAGTCGGAGGCCAAGCGGTCTTAGGAAGACAA --adapter_sequence_r2 AAGTCGGATCGTAGCCATGTCGTTCTGTGAGCCAAGGAGTTG --average_qual 15 -l 150".

Genome assembly and Hi-C scaffolding

The *de novo* genome assembly of YP4 comprised the four steps: primary assembly, Hi-C scaffolding, gap-filling, and optimization. At first, the primary contigs were generated via Hifiasm v 0.15.1 (RRID:SCR_021069) [26] with the recommend command "hifiasm -o YP4.asm -t32 --ul ul.fq.gz --h1 read1.fq.gz --h2 read2.fq.gz HiFi-reads.fq.gz". Subsequently, we used Bowtie2 v 2.2.9 (RRID:SCR_016368) [27] to align the Hi-C clean data to the primary contigs for anchoring contigs onto chromosomes. Low-quality reads were eliminated using the HiC-Pro

pipeline (RRID:SCR_017643) [28] with default parameters. The remaining valid reads were utilized to anchor chromosomes with Juicer v 1.6 (RID:SCR_017226) [29] and 3d-dna pipeline v 180419 (RRID:SCR_017227) [30]. Referring to the methods described in the gap-free genome of *Neosalanx taihuensis* [31], we applied the LR_Gapcloser (RRID:SCR_017021) [32] program to close gaps in the assembled chromosomes. To further enhance the genome quality, a polishing procedure described by Mc Cartney *et al*. 2022 [33] was implemented. Briefly, Winnowmap2 v 2.03 [34] was used to align the HiFi reads to the chromosomes, followed by filtering of alignments to exclude secondary alignments and those with excessive clipping using the 'falconc bam-filter-clipped' tool. Finally, racon v 1.5.0 (RRID:SCR_017642) [35] was performed with the filtered alignments.

The completeness of the assembly was evaluated utilizing Benchmarking Universal Single-Copy Orthologs (BUSCO) v 5.5.0 (RRID:SCR_015008) [36] based on the embryophyta_odb10 database (1614 orthologs). The quality value was generated by Merqury program v 1.3 (RRID:SCR_022964) [37] with 17-mer.

Genome annotations

We followed methods similar to those described in Qu *et al*. [38] for annotating repetitive sequences. Tandem Repeats Finder v 4.10 (RRID:SCR_022065) [39] was used to identify the tandem repeat elements. To detect interspersed repetitive sequences, we employed a strategy that combined *de novo* prediction and known repeat searching. RepeatModeler v 1.0.8 (RRID:SCR_015027) [40] and LTR_FINDER v 1.0.6 (RRID:SCR_015247) [41] were used to predict *de novo* repeat sequences. Subsequently, RepeatMasker v 4.0.7 (RRID:SCR_012954) [42]

was applied to screen the YP4 genome against the combined *de novo* transposable element library. Additionally, RepeatMasker v 4.0.7 (RRID:SCR_012954) [42] along with the Repbase database (RRID:SCR_021169) [43] were utilized to identify known transposable element repeats.

Similar to the method described for wild blueberry T2T assembly [44], telomeric sequences and centromeres region in the YP4 genome assembly were identified using quartet v 1.0.3 [45] with the "-c plant" option. The telomere repeat monomer identified by TeloExplorer module in quarTeT program was "AAACCCT".

The gene prediction process involved a comprehensive approach integrating transcriptome-based, homology-based, and *ab initio* prediction methods. Initially, RNA-seq clean reads were assembled using Trinity v 2.15.1 (RRID:SCR_013048) [46] with parameters '--max_memory 200G --CPU 40 --min_contig_length 200 --genome_guided_bam merged_sorted.bam --full_cleanup --min_kmer_cov 4 --min_glue 4 --bfly_opts '-V 5 --edge-thr=0.1 --stderr' --genome_guided_max_intron 10000'. The resulting assembled transcripts were then aligned to the assembly utilizing Program to Assemble Spliced Alignment (PASA) v 2.4.1 (RRID:SCR_014656) [47]. Gene structures were generated from valid transcript alignments (PASA-set). Additionally, RNA-seq clean reads were mapped to the assembly via Hisat2 v 2.0.1 (RRID:SCR_015530) [48]. Subsequently, Stringtie v 1.2.2 (RRID:SCR_016323) [49] and TransDecoder v 5.7.1 (RRID:SCR_017647) were employed to assemble the transcripts and identify candidate coding regions, resulting in the creation of gene models (Stringtie-set). Homologous genomes from six assemblies, including *Glycine max* (Zhonghuang 13) [50], *Glycine max* (Wm82-NJAU) [51], *Arabidopsis thaliana* [52] , *Phaseolus vulgaris* L. (G19833) [2], *Vigna angularis* (ensemble release-57), and *Medicago truncatula* (ensemble release-57) were

downloaded and used as queries to search against the assembly using GeMoMa v 1.9 (RRID:SCR_017646) [53]. These homology predictions were referred to as "Homology-set". For *ab initio* prediction methods, AUGUSTUS v 3.2.3 (RRID:SCR_008417) [54] was used to predict coding regions in the soft-masked genome. The gene models from these three sources were then merged using EvidenceModeler v 2.1.0 (RRID:SCR_014659) [55], with different weight parameters assigned to evidence from different sources (10 for PASA-set, 5 for Stringtie-set, 5 for Homology-set, and 1 for AUGUSTUS gene prediction). Finally, the generated gene models underwent further refinement with PASA v 2.4.1 (RRID:SCR_014656) [47] to obtain untranslated regions and alternative splicing variation information.

The integrated gene set was translated into amino-acid sequences and annotated using the method described in Zhou *et al.* [31]. Furthermore, we employed the RGAugury pipeline [56] to screen the whole gene set for resistance gene analogs (RGAs) gene prediction with a method similar to that described in the eggplant genome study [57]. The default *P*-value cutoff for initial RGAs gene filtering was set to le-5 for BLASTP.

Gene families and phylogenomic analysis

The OrthoMCL v2.0.9 (RRID:SCR_007839) [58] program, with default settings except for an inflation factor set at 1.5, was applied to determine gene families among eight plants, namely *A. thaliana* [52], *Cicer arietinum* (GCF_000331145), *Cajanus cajan* (GCF_000340665.1), *G. max* [51], *M. truncatula* (ensembl release-57), *Lupinus angustifolius* (ensembl release-57), *P. vulgaris* (YP4, this study), and *V. angularis* (ensembl release-57). The input for OrthoMCL comprised the results of an all-versus-all BLASTP with an E-value cutoff of 1e-5. The outcomes of gene family

clustering were summarized using UpSet (RRID:SCR_022731) [59]. A total of 1,296 single copy gene families among these species were aligned using muscle v 5.1 [60] (RRID:SCR_011812). Subsequently, the alignments of single copy gene families were concatenated into a super alignment matrix to reconstruct the phylogenetic tree via the maximum likelihood method using iqtree2 v 2.2.0 [61] with parameters of "-m MFP -B 1000". The program MCMCtree v 4.4 in the PAML package (RRID:SCR_014932) [62] was used to estimate the divergence times among the eight species, with the JC69 nucleotide substitution model and an independent rates clock. Two standard divergence time points from the TimeTree database (RRID: SCR_021162) [63] were used for calibration: 1) *A. thaliana* - *C. cajan* 102.0 - 112.5 Mya; 2) *M. truncatula* - *C. arietinum* 24.9 - 51.0 Mya. CAFE v 4.2.1 (RRID:SCR_005983) [64] was used to measure the expansion and contraction of gene families. On the basis of the maximum likelihood modeling of gene gain and loss, we analyzed gene families for signs of expansion or contraction. GO enrichment of YP4 specific genes, as well as genes in the expansion gene families, was conducted using clusterProfiler v4.2.2 (RRID:SCR_016884) [65].

Comparative genomic analysis

We performed whole genome alignment between YP4 and G19833 [2] using mummer v 4.0.0rc1 (RRID:SCR_018171) [66] with parameters: "--mum -g 1000 -c 90 -l 40". The delta-filter program was used to identify alignment blocks with the setting "-1". Subsequently, the show-snps program was utilized to detect SNPs and insertions/deletions (InDels) with the settings "-Clr -x 1 -T". SNPs and InDels were annotated using the ANNOVAR package (RRID:SCR_012821) [67].

At the gene level, pairwise synteny search was conducted using LAST v1270 (RRID:SCR_006119). The alignment results were refined using the JCVI utility libraries in MCScan (RRID:SCR_017650) (Python version) [68] with parameter: "–cscore =0.99", followed by visualization of the syntenic regions.

Results

Assembly of T2T gap-free common bean reference genome for YP4

The genome assembly of YP4 utilized multiple sequencing technologies, including PacBio HiFi reads, ONT ultra-long reads, and Hi-C reads. In summary, 31.75 Gb (~56.67× coverage) of PacBio HiFi reads, 177.04 Gb of ONT ultra-long reads (~315.97× coverage), and 144.79 Gb (~258.42× coverage) of Hi-C data (Supplementary Table S1) were generated. The N50 length of the HiFi reads exceeded 15 kb, while the N50 length of the ONT reads was over 57 kb (Supplementary Table S1; Supplementary Fig. S1; Supplementary Fig. S2). The contigs were assembled using hifiasm, resulting in 558 contigs with a total size of 606.25 Mb and an N50 length of 32.18 Mb (Supplementary Table S2). Notably, the contig N50 was 824-, and 2981-fold longer than earlier released genome versions (Table 1; Supplementary Table S2), establishing a robust basis for T2T assembly. Subsequently, the initial contigs served as the backbone to scaffold contigs into chromosomes with Hi-C data. Our result showed that the hifiasm assembly consisted of continuous sequences spanning the entire lengths of chromosomes 2 and 9, with 17 gaps distributed across 9 of the chromosomes (Supplementary Table S3). After gap filling and polishing, the final assembly achieved a total size of 560.30 Mb with an N50 of 55.11 Mb,

comprising 11 gap-free chromosomes ranging from 38.04 to 62.89 Mb in length (Fig. 2A; Table 1).

To validate the accuracy and completeness of the YP4 T2T gap-free genome assembly, multiple approaches were employed. Firstly, the Hi-C heatmap displayed a high level of consistency across all chromosomes, providing evidence for the accurate sequencing, ordering, and orientation of contigs in the YP4 genome assembly (Fig. 2B). Secondly, 100% ONT reads and 99.95% of HiFi reads were successfully aligned to the YP4 genome assembly, resulting in genome coverage of 99.49% and 98.90%, respectively. Furthermore, the Merqury-estimated quality value of YP4 was 54.86, attesting to the high accuracy of the assembly (Table 1). Thirdly, all 11 centromeres were predicted from the YP4 genome assembly, with lengths ranging from 611,691 bp to 3,362,683 bp (Fig. 2C; Supplementary Table S4). Notably, 20 out of the 22 telomeres were detected, resulting in nine telomere-to-telomere (T2T) pseudomolecules for the entire genome (Fig. 2C; Supplementary Table S5). Finally, the Benchmarking Universal Single-Copy Orthologs (BUSCO) test indicated that the YP4 assembly successfully identified 99.5% of 1,614 embryophyta gene set (Fig. 2D; Table 1). Overall, these findings demonstrate the high quality and reliability of the YP4 genome assembly.

Annotation of repetitive elements and protein-coding genes

Approximately 342.40 Mb of the assembled YP4 genome was classified as repetitive sequences, accounting for 61.20% of the genome. This percentage is higher than that in G19833 (45.42%) and BAT93 (35.50%; Table 1; Supplementary Table S6). Among the repetitive sequences, the majority were long terminal repeats (LTRs), which comprised 36.48% of the genome.

(Supplementary Table S7). The DNA, long interspersed nuclear elements (LINE), and short interspersed nuclear elements (SINE) classes accounted for 4.24%, 2.58%, and 0.11% of the genome, respectively (Supplementary Table S7).

To facilitate genome annotation of the YP4 assembly, RNA sequencing was performed on various tissues, including root, stem, leaf, flower, and pod. A total of 118.68 Gb clean reads were obtained (Supplementary Table S8). Through a combined prediction strategy, 29,925 protein-coding genes were predicted, with mean lengths of 4,042 bp for the gene, 710 bp for the intron, and 1241 bp for the coding sequence (Supplementary Table S9). The BUSCO assessment of the predicted gene sets showed 98.7% completeness and only 0.37% missing genes, indicating the robustness of the gene annotation (Supplementary Table S10). The length distribution of messenger RNA, coding sequences, exons, and introns among related species further supported the reliability of the annotation results (Supplementary Fig. S3). Among the predicted genes, 29,426 (98.33%) carried at least one conserved functional domain (Supplementary Table S11). Additionally, 1,339 resistance gene analogs (RGAs) were identified in the YP4 assembly, a higher number compared to the 852 RGAs found in the BAT93 genome [7] (Fig. 3A). The largest category among the RGAs was receptor-like kinases (RLKs), comprising a total of 720 genes. Notably, 96.57% the RNA-seq reads were aligned to the predicted exons (Fig. 3B). Moreover, 23,006 (78.18%) genes had an FPKM value above 1.0 in at least one RNA-seq sample (Supplementary Fig. S4). These results confirmed the completeness and accuracy of gene prediction across YP4 genome.

Phylogenetic relationship analysis

The protein-coding genes of seven plant species, including *A. thaliana*, *C. arietinum*, *C. cajan*, *G. max*, *M. truncatula*, *L.angustifolius*, and *V. angularis*, were clustered into 25,888 gene families together with the protein-coding genes of YP4 (Fig. 4A). Specifically, 294 gene families containing 1,755 genes were identified as specific to YP4 when compared with the other 7 plant species (Supplementary Table S12). Among these YP4-specific genes, 1,557 (88.72%) were supported by functional annotation, and they were significantly enriched in 23 GO terms (Supplementary Table S13). The top 10 most significantly enriched GO terms included "nucleic acid binding", "zinc ion binding", "inositol catabolic process", "inositol oxygenase activity", "nutrient reservoir activity", "structural constituent of cell wall", "manganese ion transmembrane transporter activity", "cellular manganese ion homeostasis", "response to auxin", and "ribonuclease P complex" (Supplementary Fig. S5). A phylogenetic tree was constructed for the 8 plant species, with *A. thaliana* considered as an outgroup (Fig. 4B). The estimated divergence time between YP4 and *V. angularis* was approximately 11.6 million years ago (MYA). Comparing with the most recent common ancestor (MRCA), YP4 showed 73 expansion and 14 contraction events of each gene family (Fig. 4B). The expanded gene families of YP4 were mainly enriched in functions such as "ADP binding," "defense response," "signal transduction," "terpene synthase activity," "lyase activity," "magnesium ion binding," "manganese ion binding," "hydrolase activity, hydrolyzing O-glycosyl compounds," "phosphoric diester hydrolase activity," "carbohydrate metabolic process," and others (Supplementary Fig. S6).

Comparison of YP4 and G19833 genomes

YP4 exhibited a longer assembly length compared to G19833, with 20 telomeres assembled in YP4 but none in G19833 (Table 1; Fig. 5A). Additionally, all 40,860 gaps present in the G19833 assembly were successfully filled in the G42 assembly, achieving complete gap closure. The JCVI analysis showed high collinearity between YP4 and G19833 (Fig. 5B). The syntenic regions contained 23,539 orthologous pairs, with 78.66% in YP4 and 83.43% in G19833. Given that the contig N50 of G19833 was only 39,053, indicating a lack of genomic continuity in the assembly, our focus was on the variations of SNPs and InDels between YP4 and G19833. A total of 1,203,386 SNPs and 317,537 InDels were detected between the two genomes (Supplementary Fig. S7). Among these variations, 44,734 (3.72%) SNPs and 3,126 (0.98%) InDels located in exonic regions (Supplementary Table S14; Supplementary Table S15). Specifically, there were 23,753 SNPs and 2,008 InDels that potentially affecting gene function, associated with 6,930 genes (Supplementary Table S16). GO enrichment analysis highlighted significant enrichments in 11 terms, including "ADP binding", "defense response", "ATP binding", "protein kinase activity", "protein phosphorylation", "protein binding", "protein serine/threonine kinase activity", "sulfotransferase activity", "oxidoreductase activity, acting on paired donors, with incorporation or reduction of molecular oxygen", "monooxygenase activity", and "recognition of pollen" (Supplementary Fig. S8).

Conclusions

The first T2T gap-free genome assembly of a typical common bean, YP4, was successfully achieved using PacBio HiFi reads, ONT ultra-long sequencing, and Hi-C technologies. This assembly is characterized by its high level of completeness and accuracy. A total of 11

chromosomes were assembled, with 9 chromosomes meeting the telomere-to-telomere standard. In addition, the assembly predicted a total of 342.40 Mb repetitive sequences and identified 29,925 protein-coding genes. Evolutionary analysis indicates that exploring defense response may be a promising avenue for understanding the genetic characteristics of common beans, further supported by comparative genomics analysis. Overall, this dataset provides a valuable resource for future research in the genetic breeding of common beans.

Data Availability

The genome assembly and all the sequencing data have been deposited in GenBank database under the accession number PRJNA1072282.

Figure

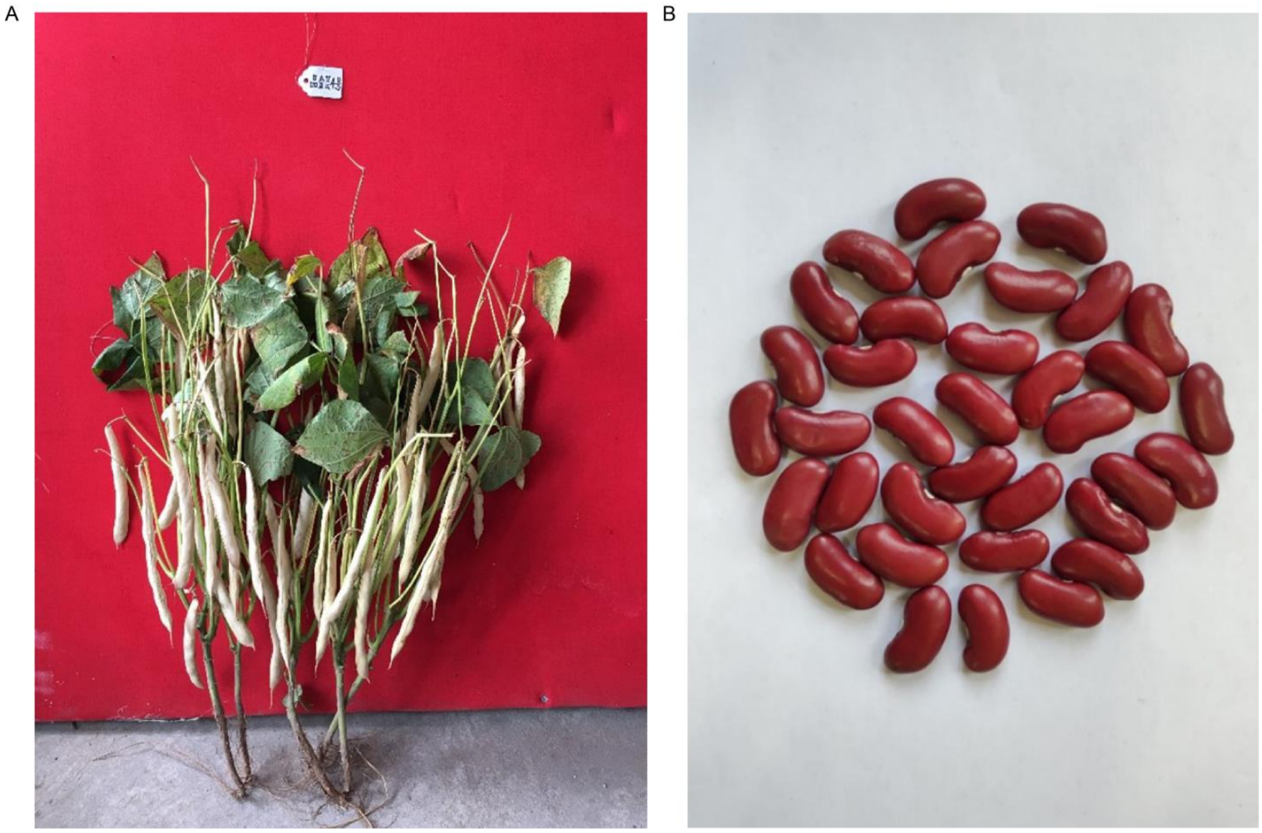

Fig. 1 | The YP4 plant sequenced in this study.A, The plant of YP4. **B**, The beans of YP4.

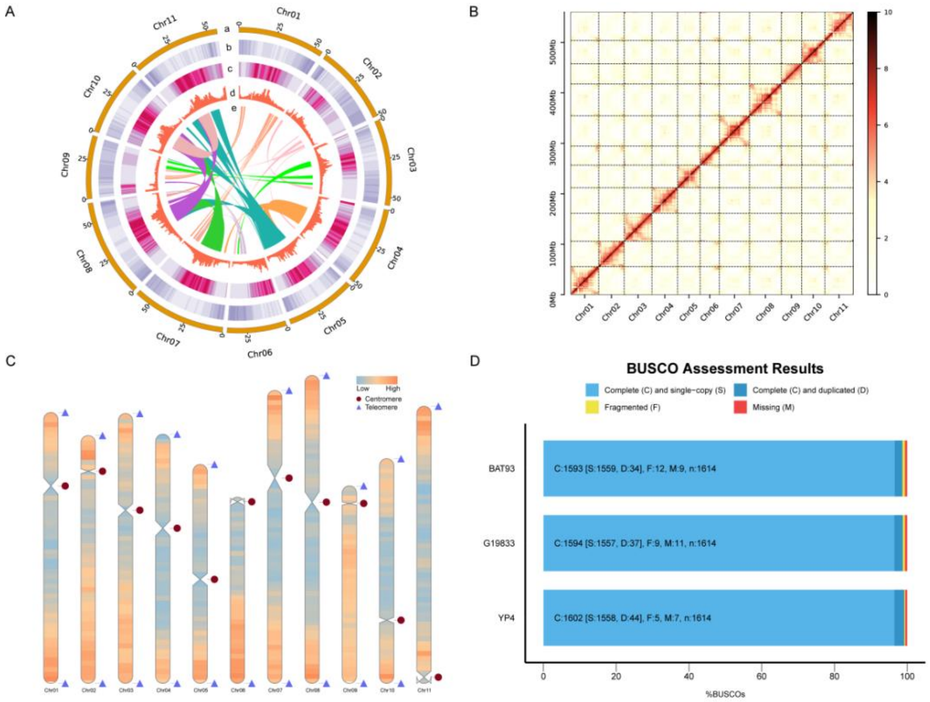

Fig. 2 | High-quality reference of YP4 genome. A, Circos plot showing the characterization of YP4 genome. From outside to inside: a, The length of pseudo-chromosome in the size of Mb. b, gene density in 1-Mb sliding windows. c, percentage of repetitive elements in 1 Mb sliding windows. d, GC content in non-overlapping 1Mb windows. e, collinear regions within the YP4 assembly. **B**, Heatmap displaying Hi-C interactions of YP4 pseudomolecules. Chr01 - Chr11 are the abbreviations of 11 Chromosome. The abscissa and ordinate represent the order of each bin on the corresponding chromosome group. The colour block illuminates the intensity of interaction from yellow (low) to red (high). **C**, Telomere and centromere detection map. Triangles and circles represent telomeres and centromere within the YP4 assembled chromosomes. The orange color represents regions with high gene density, while the sky blue color represents regions with low gene density. **D**, BUSCO assessments of the YP4, G19833, and BAT93 genome.

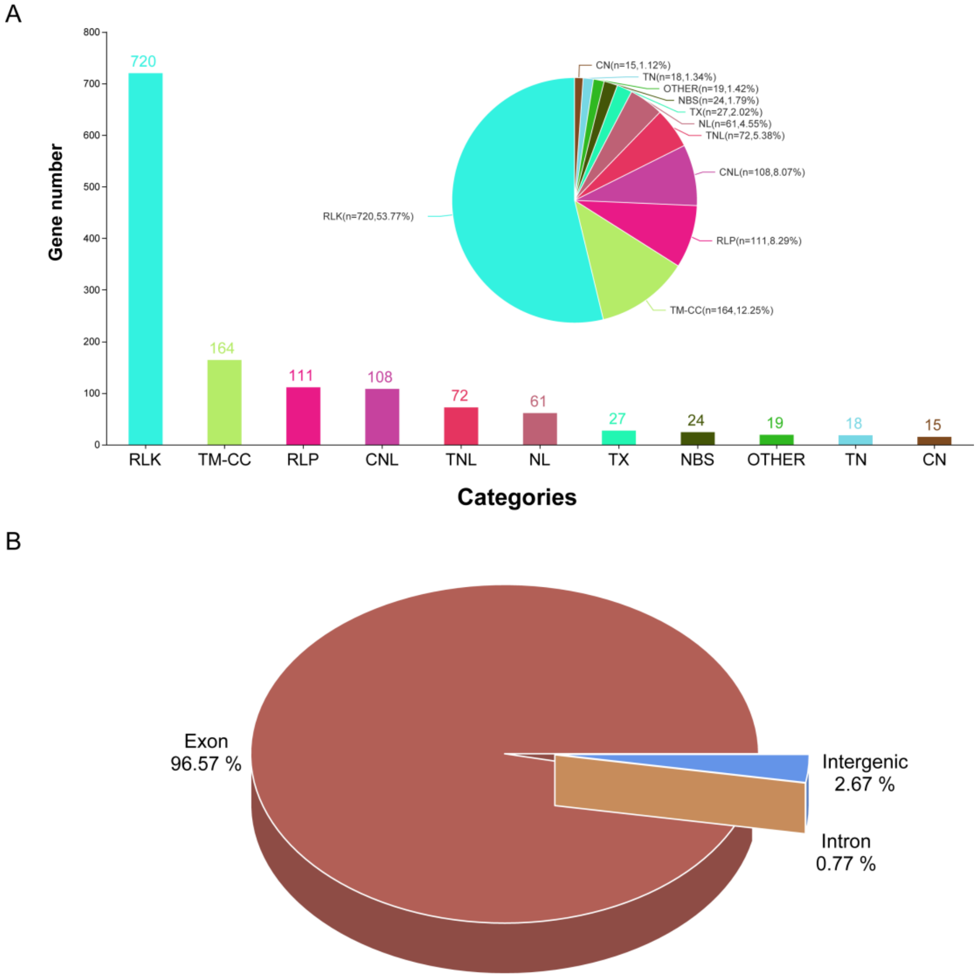

Fig. 3 | The gene annotation of YP4 assembly. A, Summary of RGAs categories in YP4 assembly. RLK, receptor-like kinase; TM, transmembrane; CC, coiled-coil; RLP, receptor-like protein; CNL, CC-NBS-LRR; TNL, TIR-NBS-LRR; NL, NBS-LRR; TX, TIR-unknown domain; NBS, nucleotide-binding site; TN, TIR-NBS; CN, CC-NBS. **B,** RNA-seq clean data verified the accuracy of protein-coding gene prediction.

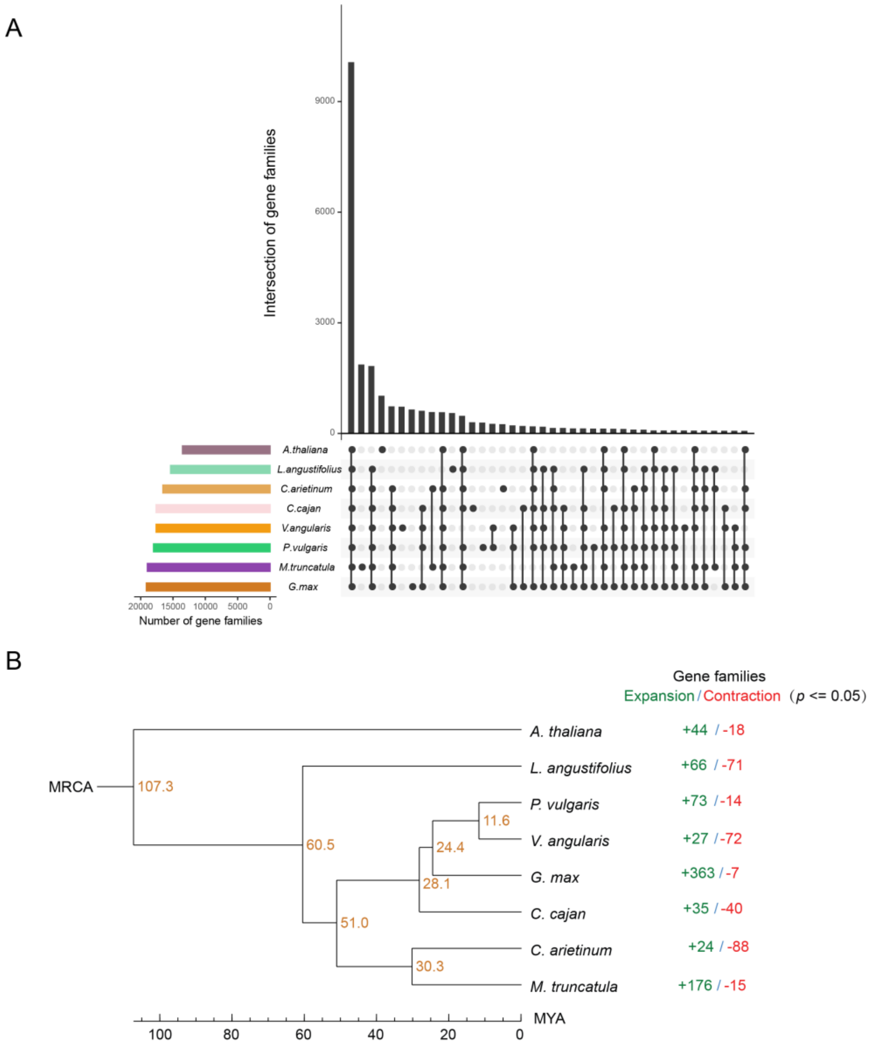

Fig. 4 | Evolution of the YP4 genome. A, UpSetPlot representing the intersections of gene families among the 8 species. Rows and columns represent gene families and intersections, respectively. Black and gray circles indicate the existence or absence of a given intersection. The horizontal bar chart on the left side of the matrix indicates the size of gene family. **B,** Phylogenetic tree of the 8 species. Numbers on nodes indicate the differentiation time. MRCA, most recent common ancestor.

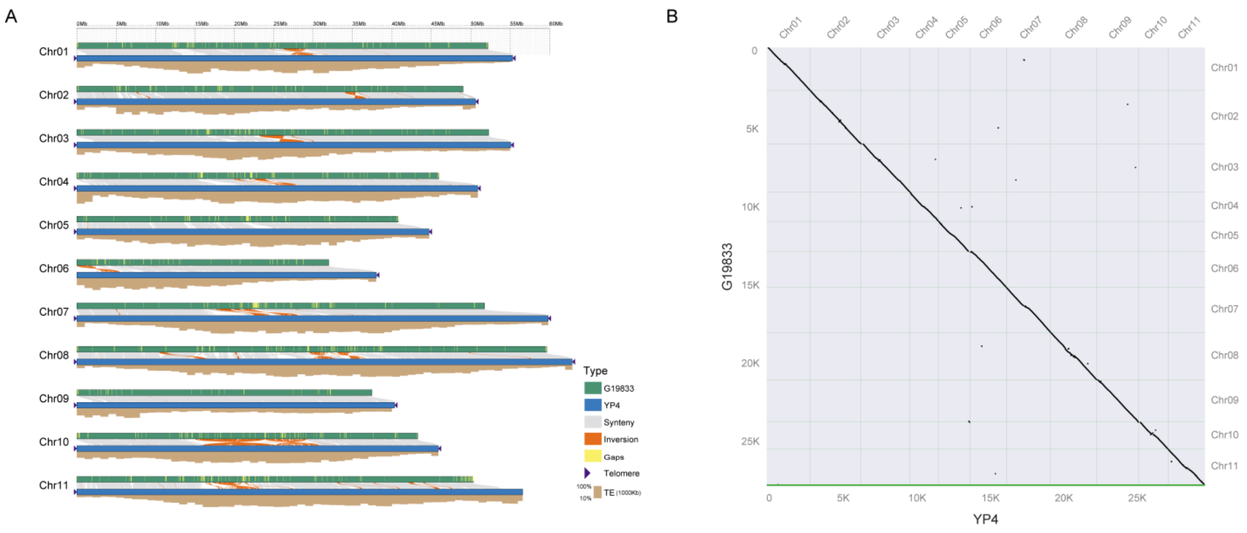

Fig. 5 | Genomic comparision between YP4 and G19833. A, Collinearity between YP4 and G19833. Gray lines illustrate collinear regions between YP4 and G19833. Triangles denote the presence of telomere sequence repeats in YP4. The yellow bar indicates gap regions in G19833. **B,** Dot-plot alignment between YP4 and G19833.

Abbreviations

BLAST: Basic Local Alignment Search Tool; BUSCO: Benchmarking Universal Single-Copy Orthologs; Gb: gigabase pairs; GO: Gene Ontology; Hi-C: High-Throughput Chromosome Conformation Capture; HiFi: High-Fidelity; KEGG: Kyoto Encyclopedia of Genes and Genomes; InDels: insertions/deletions; LINE: long interspersed nuclear element; LTR: long terminal repeat; Mb: megabase pairs; MRCA: most recent common ancestor; MYA: million years ago; RGAs: resistance gene analogs; RNA-seq: RNA sequencing; PASA: Program to Assemble Spliced Alignments; SMRT: Single-Molecule Real-time Sequencing; SNPs: single nucleotide polymorphisms; T2T: telomere-to-telomere

Additional Files

Supplementary Table 1. Summary of whole genome sequencing data of YP4 genome.

Supplementary Table 2. The statistics of the hifiasm assembly.

Supplementary Table 3. The statistics of the anchored chromosome length.

Supplementary Table 4. The distribution of centromeres in YP4 assembly.

Supplementary Table 5. The identified telomeres in YP4 assembly.

Supplementary Table 6. General statistics of repeats in the YP4 assembly.

Supplementary Table S7. The summary of interspersed repeat contents in YP4 assembly.

Supplementary Table S8. The summary of RNAseq sequencing quality.

Supplementary Table S9. Summary of gene structure prediction in YP4 genome.

Supplementary Table S10. BUSCOs analysis of YP4 gene set completeness.

Supplementary Table S11. Number of functional annotations for predicted genes in the YP4 assembly.

Supplementary Table S12. Gene families in YP4 and other species.

Supplementary Table S13. The list of YP4 specific genes.

Supplementary Table S14. The annotation of SNPs between YP4 and G19833.

Supplementary Table S15. The annotation of Indels between YP4 and G19833.

Supplementary Table S16. The gene list impacted by variation between YP4 and G19833.

Funding

This study is supported by the National Natural Science Foundation of China (32241041), The Major Special Science and Technology Projects in Shanxi Province (202101140601027), China

Agriculture Research System of MOF and MARA-Food Legumes (CARS-08), The National Key Research and Development Program of China(2021YFD1600600), Biological Breeding Engineering of Shanxi Agricultural University(YZGC148),

Ethical Approval

No ethical issues were involved in this study.

Competing Interests

The authors declare that they have no competing interests.

Authors' Contribution

Z. X.M., J.W.C., J.W. and H.G.W. conceived and developed the research; Y.W. and X.P.H. collected the samples, conducted experiments and analyzed the data; C.H.C, P.G. and X.K.Y. performed the data analysis; Y.W., X.P.H. and C.H.C. wrote the first draft of the manuscript; X.D., M.L., H.B.Q and S.H. participated in the research discussions and provided comments to improve the manuscript.

Acknowledgements

We thank every project that provides funding and material support for the study. We also thank each author for their ideas and skills in study design, experimentation, data collection, data analysis and manuscript writing. We sincerely thank the editors and reviewers for their valuable suggestions and comments on this study.

References

1. Graham, P.H. and C.P. Vance, *Legumes: importance and constraints to greater use.* Plant physiology, 2003. **131**(3): p. 872-877.
2. Schmutz, J., et al., *A reference genome for common bean and genome-wide analysis of dual domestications.* Nature Genetics, 2014. **46**: p. 707 - 713.
3. Geil, P.B. and J.W. Anderson, *Nutrition and health implications of dry beans: a review.* Journal of the American College of Nutrition, 1994. **13 6**: p. 549-58.
4. Cichy, K.A., et al., *QTL Analysis of Seed Iron, Zinc, and Phosphorus Levels in an Andean Bean Population.* Crop science, 2009. **49**(5): p. 1742-1750.
5. Beebe, S., *Common bean breeding in the tropics.* Plant Breeding Reviews, 2012. **36**: p. 357-426.
6. Mamidi, S., et al., *Demographic factors shaped diversity in the two gene pools of wild common bean Phaseolus vulgaris L.* Heredity, 2013. **110**(3): p. 267-276.
7. Vlasova, A., et al. *Genome and transcriptome analysis of the Mesoamerican common bean and the role of gene duplications in establishing tissue and temporal specialization of genes*. Genome biology, 2016. **17**, 32 DOI: 10.1186/s13059-016-0883-6.
8. Wu, J., et al., *Resequencing of 683 common bean genotypes identifies yield component trait associations across a north-south cline.* Nature genetics, 2020. **52**(1): p. 118-125.
9. Meziadi, C., et al., *Development of molecular markers linked to disease resistance genes in common bean based on whole genome sequence.* Plant science : an international journal of experimental plant biology, 2016. **242**: p. 351-357.
10. Kang, Y.J., et al. *Genome-wide mapping of NBS-LRR genes and their association with disease resistance in soybean*. BMC plant biology, 2012. **12**, 139 DOI: 10.1186/1471-2229-12-139.
11. Abdulla, M.F., et al., *GATA transcription factor in common bean: A comprehensive genome-wide functional characterization, identification, and abiotic stress response evaluation.* Plant Molecular Biology, 2023. **114**.
12. Okay, A., et al., *Omics approaches to understand the MADS-box gene family in common bean (Phaseolus vulgaris L.) against drought stress.* Protoplasma, 2024.
13. Akbulut, S.E., et al., *The genome-wide characterization of WOX gene family in Phaseolus vulgaris L. during salt stress.* Physiology and molecular biology of plants : an international journal of functional plant biology, 2022. **28**(6): p. 1297-1309.
14. Payne, Z.L., et al., *A gap-free genome assembly of Chlamydomonas reinhardtii and detection of translocations induced by CRISPR-mediated mutagenesis.* Plant Communications, 2023. **4**(2): p. 100493.
15. Xie, L., et al., *Technology-enabled great leap in deciphering plant genomes.* Nature plants, 2024. **10**(4): p. 551-566.
16. Shang, L., et al., *A complete assembly of the rice Nipponbare reference genome.* Molecular plant, 2023. **16**(8): p. 1232-1236.
17. Chen, J., et al., *A complete telomere-to-telomere assembly of the maize genome.* Nature genetics, 2023. **55**(7): p. 1221-1231.
18. Huang, Y., et al., *A complete reference genome for the soybean cv. Jack.* Plant communications, 2024. **5**(2): p. 100765.
19. Ding, Y., et al., *A telomere-to-telomere genome assembly of Hongyingzi, a sorghum cultivar used for Chinese Baijiu production.* The Crop Journal, 2024.
20. Porebski, S., L.G. Bailey, and B.R.B.R. Baum, *Modification of a CTAB DNA extraction protocol for plants*

containing high polysaccharide and polyphenol components. Plant Molecular Biology Reporter, 1997. **15**: p. 8-15.

21. Chin, C., et al., *Nonhybrid, finished microbial genome assemblies from long-read SMRT sequencing data.* Nature Methods, 2013. **10**: p. 563-569.
22. De Coster, W., et al., *NanoPack: visualizing and processing long-read sequencing data.* Bioinformatics, 2018. **34**: p. 2666 - 2669.
23. Belton, J.-M., et al., *Hi-C: a comprehensive technique to capture the conformation of genomes.* Methods, 2012. **58 3**: p. 268-76.
24. Chen, Y., et al., *SOAPnuke: a MapReduce acceleration-supported software for integrated quality control and preprocessing of high-throughput sequencing data.* GigaScience, 2017. **7**.
25. Chen, S., et al., *fastp: an ultra-fast all-in-one FASTQ preprocessor.* Bioinformatics, 2018. **34**(17): p. i884-i890.
26. Cheng, H., et al., *Haplotype-resolved de novo assembly using phased assembly graphs with hifiasm.* Nature Methods, 2021. **18**(2): p. 170-175.
27. Langmead, B. and S.L. Salzberg, *Fast gapped-read alignment with Bowtie 2.* Nature Methods, 2012. **9**(4): p. 357-359.
28. Servant, N., et al., *HiC-Pro: An optimized and flexible pipeline for Hi-C data processing.* Genome Biology, 2015. **16**.
29. Durand, N., et al., *Juicer Provides a One-Click System for Analyzing Loop-Resolution Hi-C Experiments.* Cell Systems, 2016. **3**: p. 95-98.
30. Dudchenko, O., et al., *De novo assembly of the Aedes aegypti genome using Hi-C yields chromosome-length scaffolds.* Science, 2017. **356**: p. eaal3327.
31. Zhou, Y., et al. *Gap-free genome assembly of Salangid icefish Neosalanx taihuensis*. Scientific data, 2023. **10**, 768 DOI: 10.1038/s41597-023-02677-z.
32. Xu, G.-C., et al., *LR_Gapcloser: a tiling path-based gap closer that uses long reads to complete genome assembly.* GigaScience, 2018. **8**.
33. Mc Cartney, A.M., et al., *Chasing perfection: validation and polishing strategies for telomere-to-telomere genome assemblies.* Nature methods, 2022. **19**(6): p. 687-695.
34. Jain, C., et al., *Long-read mapping to repetitive reference sequences using Winnowmap2.* Nature methods, 2022. **19**(6): p. 705-710.
35. Vaser, R., et al., *Fast and accurate de novo genome assembly from long uncorrected reads.* Genome research, 2017. **27 5**: p. 737-746.
36. Seppey, M., M. Manni, and E.M. Zdobnov, *BUSCO: Assessing Genome Assembly and Annotation Completeness.* Methods in molecular biology (Clifton, N.J.), 2019. **1962**: p. 227-245.
37. Rhie, A., et al., *Merqury: reference-free quality, completeness, and phasing assessment for genome assemblies.* Genome Biology, 2020. **21**.
38. Qu, C., et al., *Comparative genomic analyses reveal the genetic basis of the yellow-seed trait in Brassica napus.* Nature Communications, 2023. **14**(1): p. 5194.
39. Benson, G., *Tandem repeats finder: a program to analyze DNA sequences.* Nucleic acids research, 1999. **27 2**: p. 573-80.
40. Flynn, J.M., et al., *RepeatModeler2 for automated genomic discovery of transposable element families.* Proceedings of the National Academy of Sciences of the United States of America, 2020. **117**(17): p. 9451-9457.
41. Xu, Z. and H. Wang, *LTR_FINDER: an efficient tool for the prediction of full-length LTR retrotransposons.* Nucleic Acids Research, 2007. **35**: p. W265 - W268.

42. Chen, N., *Using RepeatMasker to Identify Repetitive Elements in Genomic Sequences.* Current Protocols in Bioinformatics, 2004. **5**.
43. Bao, W., K.K. Kojima, and O. Kohany, *Repbase Update, a database of repetitive elements in eukaryotic genomes.* Mobile DNA, 2015. **6**.
44. Zeng, T., et al., *The Telomere-to-telomere gap-free reference genome of wild blueberry (Vaccinium duclouxii) provides its high soluble sugar and anthocyanin accumulation.* Horticulture Research, 2023.
45. Lin, Y., et al., *quarTeT: a telomere-to-telomere toolkit for gap-free genome assembly and centromeric repeat identification.* Horticulture Research, 2023.
46. Grabherr, M.G., et al., *Full-length transcriptome assembly from RNA-Seq data without a reference genome.* Nature biotechnology, 2011. **29 7**: p. 644-52.
47. Haas, B., *Improving the Arabidopsis genome annotation using maximal transcript alignment assemblies.* Nucleic Acids Research, 2003. **31**: p. 5654-5666.
48. Kim, D., B. Langmead, and S.L. Salzberg, *HISAT: a fast spliced aligner with low memory requirements.* Nature Methods, 2015. **12**(4): p. 357-360.
49. Kovaka, S., et al., *Transcriptome assembly from long-read RNA-seq alignments with StringTie2.* Genome Biology, 2019. **20**(1): p. 278.
50. Zhang, A., et al., *A telomere-to-telomere genome assembly of Zhonghuang 13, a widely-grown soybean variety from the original center of Glycine max.* The Crop Journal, 2023.
51. Wang, L., et al., *A telomere-to-telomere gap-free assembly of soybean genome.* Molecular plant, 2023.
52. Hou, X., et al., *A near-complete assembly of an Arabidopsis thaliana genome.* Molecular plant, 2022.
53. Jens, et al., *GeMoMa: Homology-Based Gene Prediction Utilizing Intron Position Conservation and RNA-seq Data.* Methods in Molecular Biology, 2019.
54. Stanke, M. and B. Morgenstern, *AUGUSTUS: a web server for gene prediction in eukaryotes that allows user-defined constraints.* Nucleic acids research, 2005. **33**(Web Server issue): p. W465-7.
55. Haas, B.J., et al., *Automated eukaryotic gene structure annotation using EVidenceModeler and the Program to Assemble Spliced Alignments.* Genome Biology, 2008. **9**(1): p. R7.
56. Li, P., et al., *RGAugury: a pipeline for genome-wide prediction of resistance gene analogs (RGAs) in plants.* BMC Genomics, 2016. **17**.
57. Li, D., et al., *A high-quality genome assembly of the eggplant provides insights into the molecular basis of disease resistance and chlorogenic acid synthesis.* Molecular ecology resources, 2021. **21**(4): p. 1274-1286.
58. Li, L., C.J. Stoeckert, and D.S. Roos, *OrthoMCL: identification of ortholog groups for eukaryotic genomes.* Genome research, 2003. **13**(9): p. 2178-2189.
59. Lex, A., et al., *UpSet: Visualization of Intersecting Sets.* IEEE transactions on visualization and computer graphics, 2014. **20**(12): p. 1983-1992.
60. Edgar, R.C., *Muscle5: High-accuracy alignment ensembles enable unbiased assessments of sequence homology and phylogeny.* Nature Communications, 2022. **13**(1): p. 6968.
61. Minh, B.Q., et al., *IQ-TREE 2: New Models and Efficient Methods for Phylogenetic Inference in the Genomic Era.* Molecular Biology and Evolution, 2019. **37**: p. 1530 - 1534.
62. Yang, Z., *PAML: a program package for phylogenetic analysis by maximum likelihood.* Computer applications in the biosciences : CABIOS, 1997. **13 5**: p. 555-6.
63. Hedges, S.B., J.T. Dudley, and S. Kumar, *TimeTree: a public knowledge-base of divergence times among organisms.* Bioinformatics, 2006. **22 23**: p. 2971-2.
64. Bie, T.D., et al., *CAFE: a computational tool for the study of gene family evolution.* Bioinformatics, 2006. **22 10**: p. 1269-71.
65. Wu, T., et al., *clusterProfiler 4.0: A universal enrichment tool for interpreting omics data.* The Innovation,

2021. **2**.

66. Marçais, G., et al., *MUMmer4: A fast and versatile genome alignment system.* PLoS Computational Biology, 2018. **14**.

67. Wang, K., M. Li, and H.H. Hakonarson, *ANNOVAR: functional annotation of genetic variants from high-throughput sequencing data.* Nucleic Acids Research, 2010. **38**: p. e164 - e164.

68. Tang, H., et al., *Synteny and Collinearity in Plant Genomes.* Science, 2008. **320**: p. 486 - 488.

 Click here to access/download;Table;Table1.xlsx

Table 1. Comparison of three common bean assemblies

Assembly feature	YP4	*G19833	†BAT93
Size of assembly	560,297,700	521,076,696	549,748,340
Contig N50	55,110,595	39,053	10,795
Scaffold N50	55,110,595	50,367,376	39,037,607
Longest scaffold	62,894,056	59,662,532	50,710,336
Number of gaps in chromosomes	0	40,860	45,300
Number of protein-coding genes	29,925	28,134	30,491
Repetitive elements	61.20%	45.42%	35.50%
Quality value	54.86	-	-
Complete BUSCOs (N=1,614)	99.50%	99.40%	99.40%

*G19833 from Ensembl database (release-56)

†BAT93 from NCBI database under accession GCA_001517995.1

Figure1
Click here to access/download;Figure;Figure1.pdf

A B

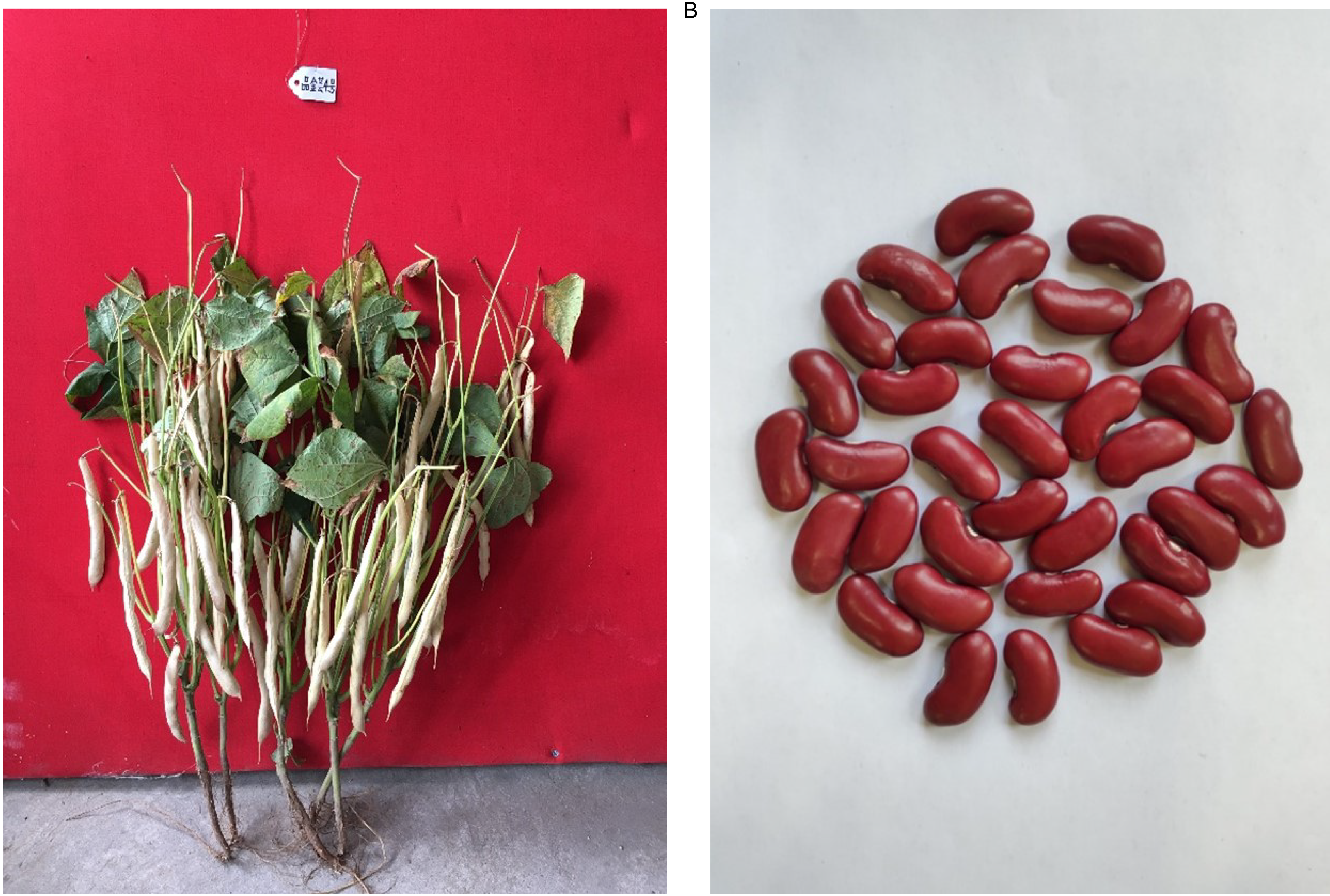

Figure2

Click here to access/download;Figure;Figure2.pdf

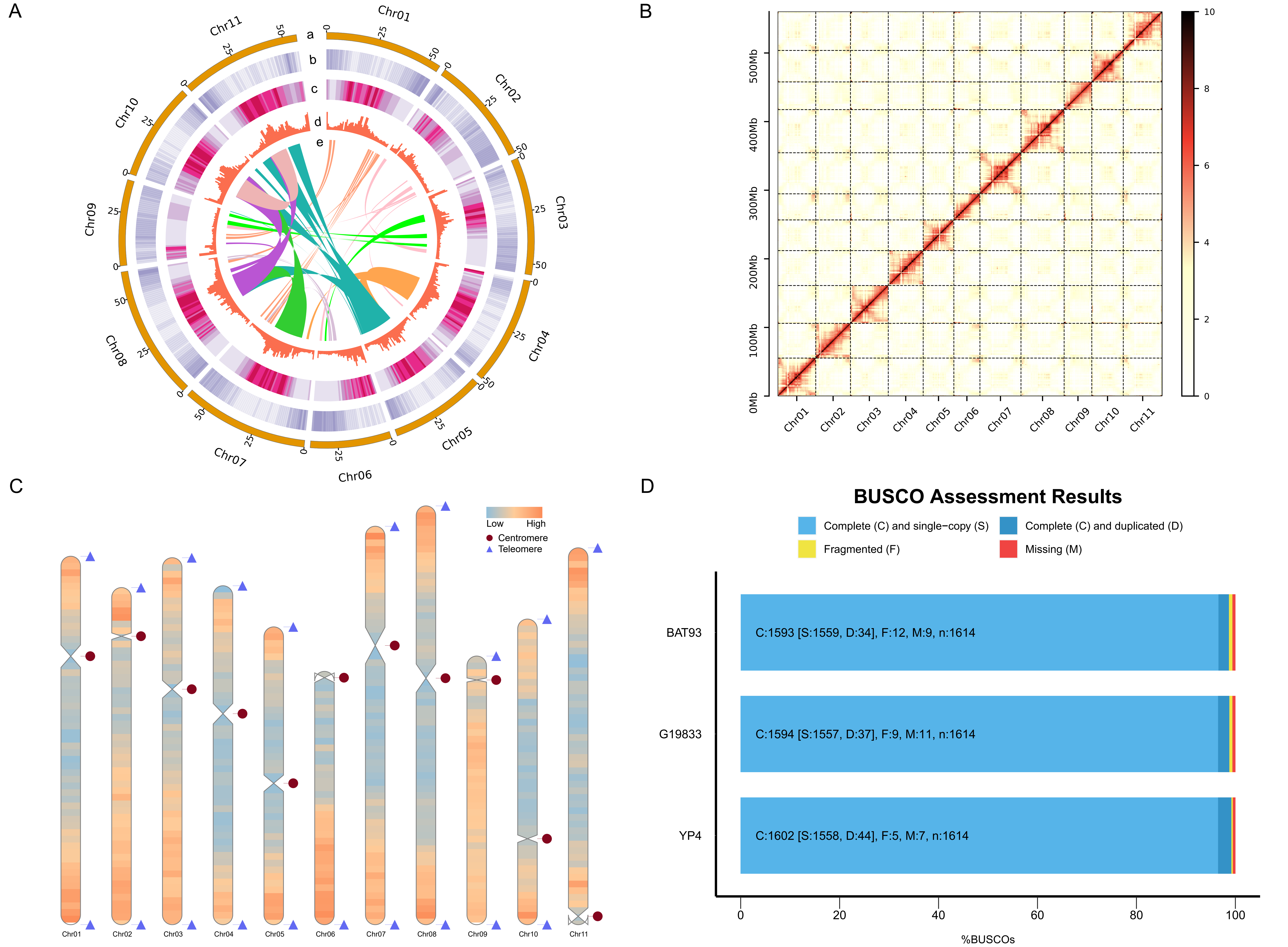

Figure3

Click here to access/download;Figure;Figure3.pdf

A

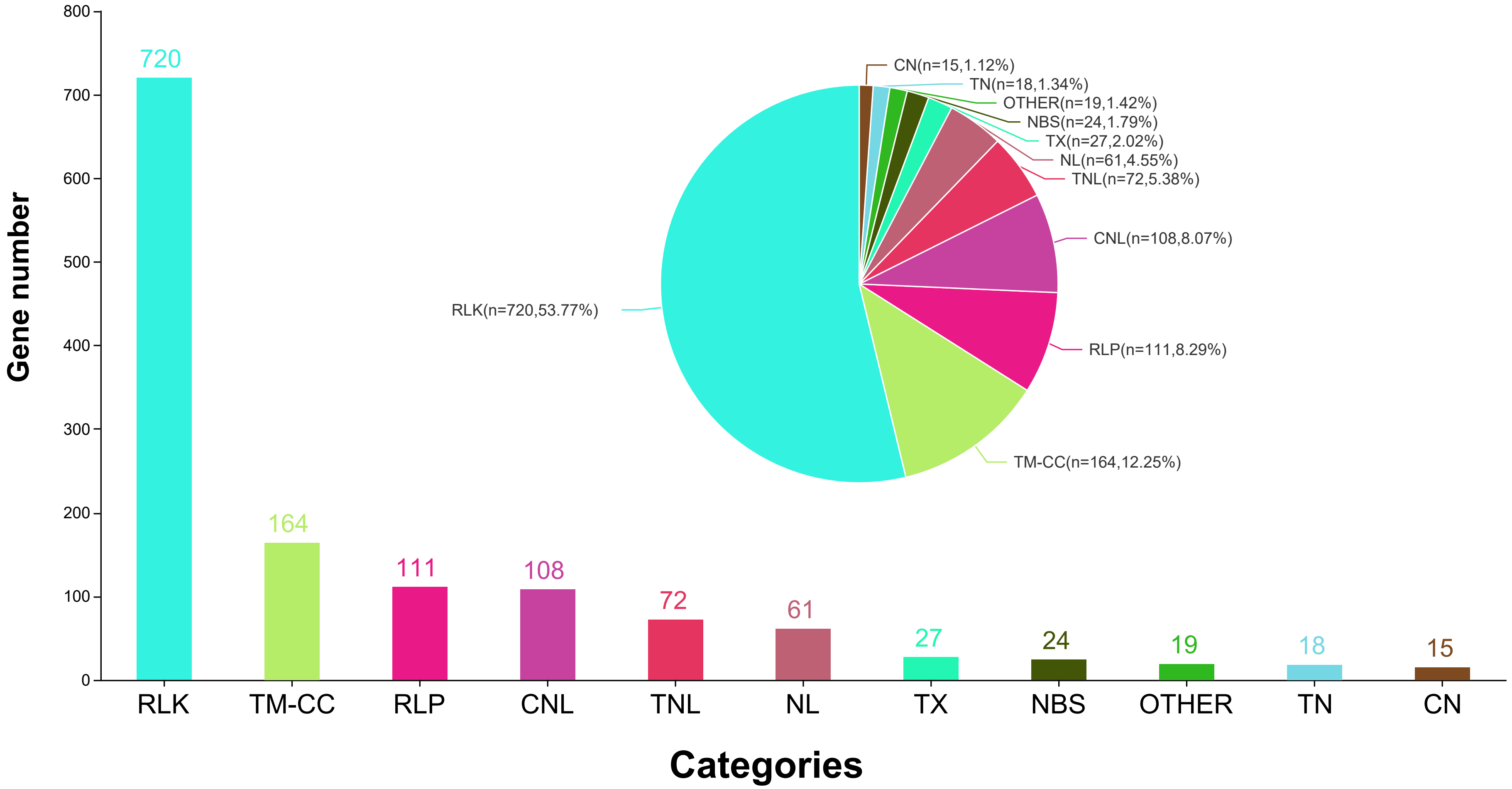

B

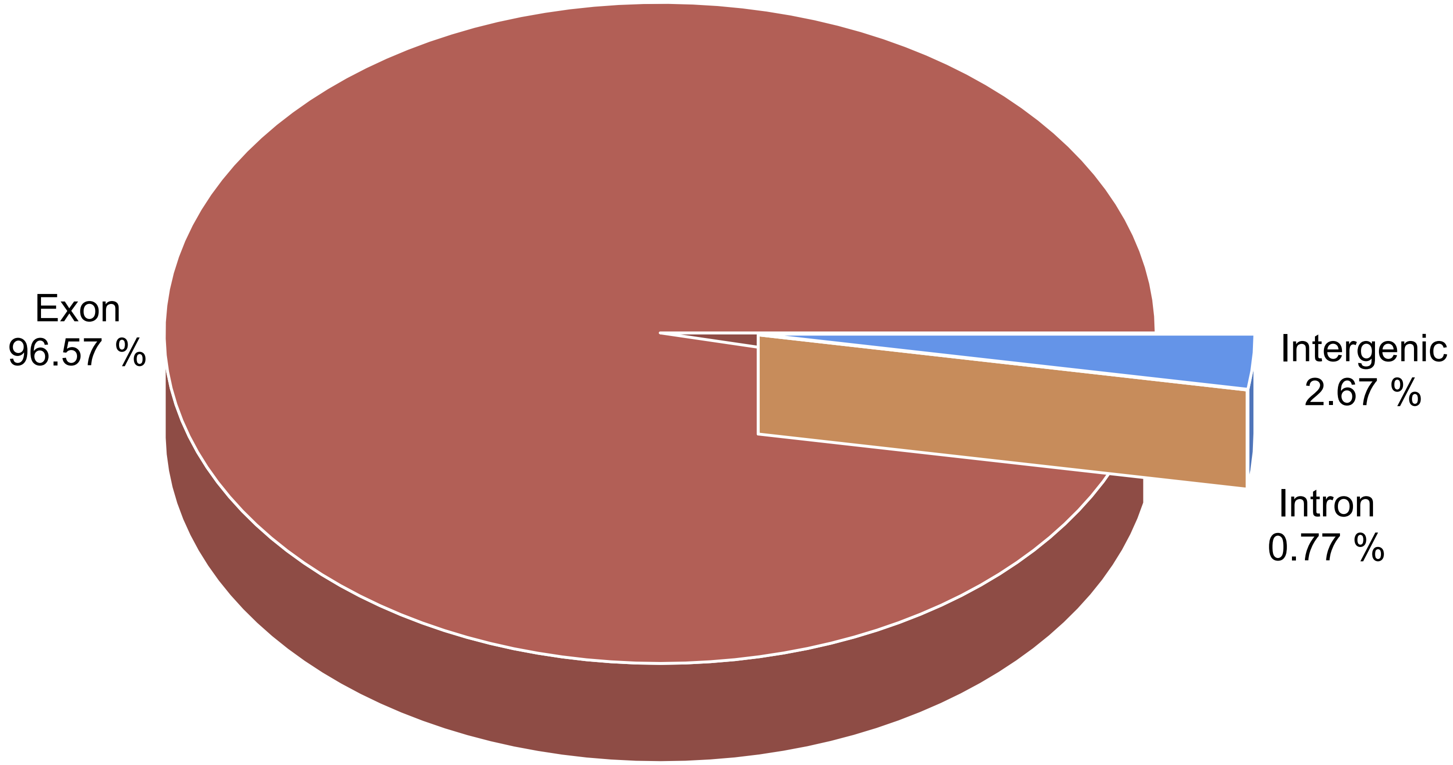

Figure4

Click here to access/download;Figure;Figure4.pdf

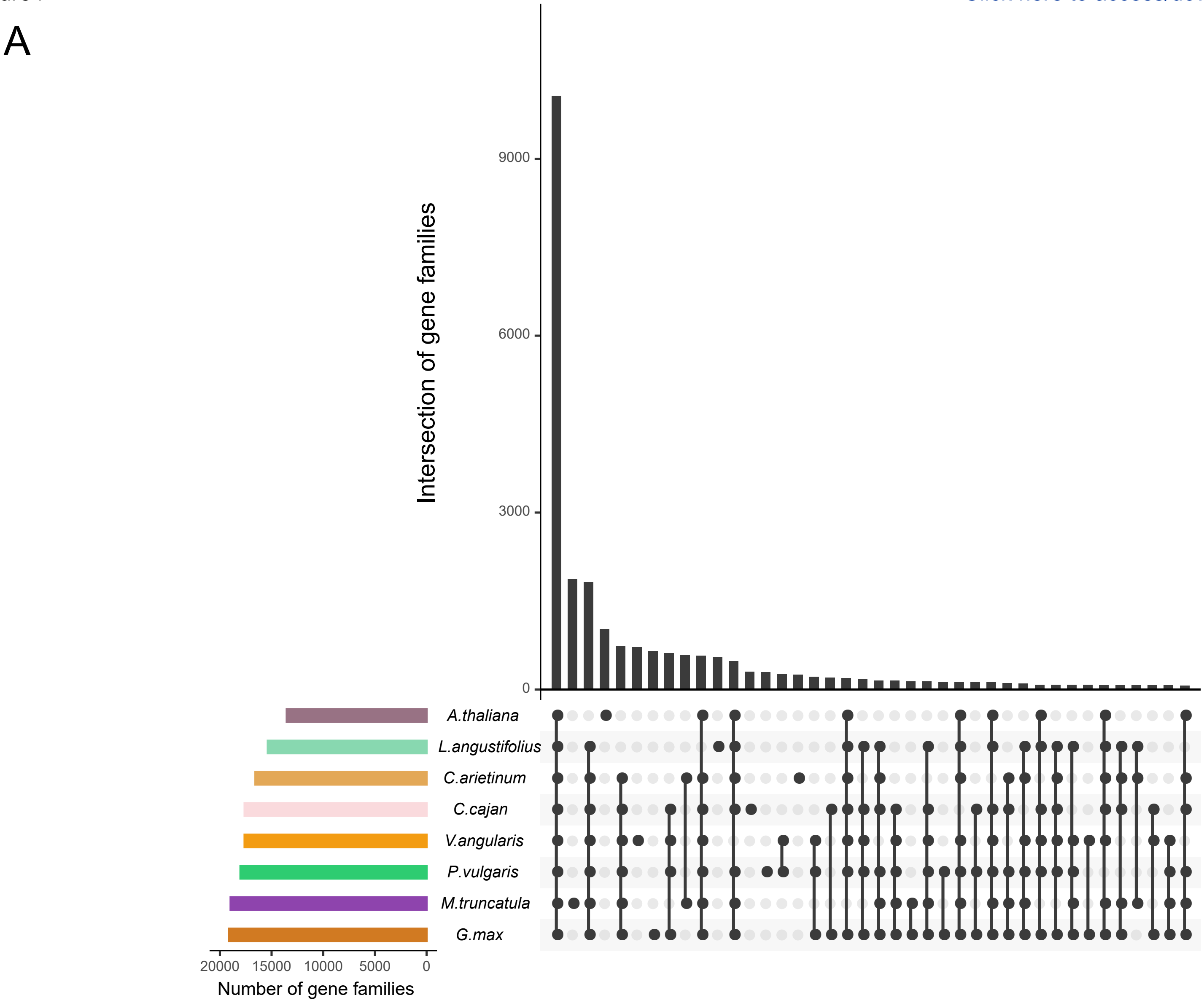

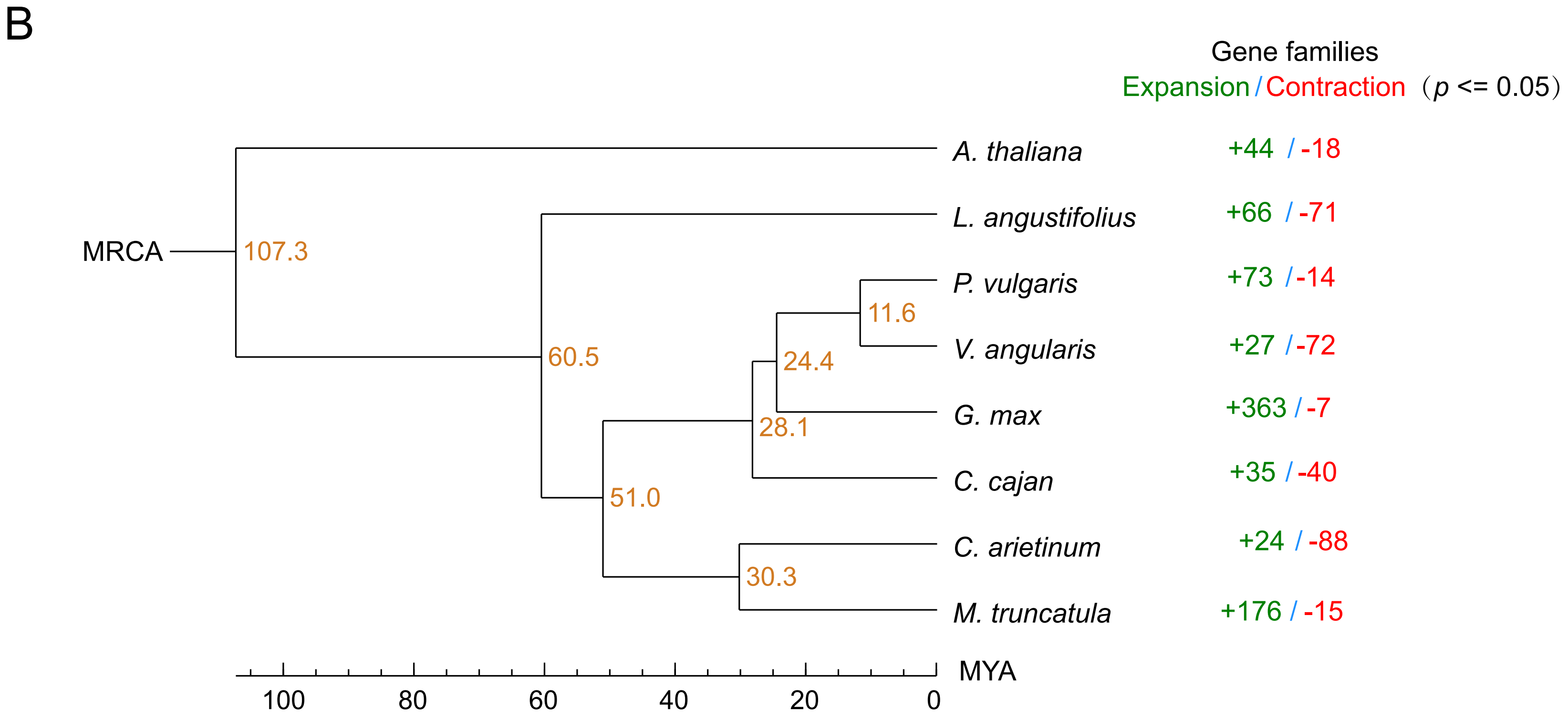

Figure5

Click here to access/download;Figure;Figure5.pdf

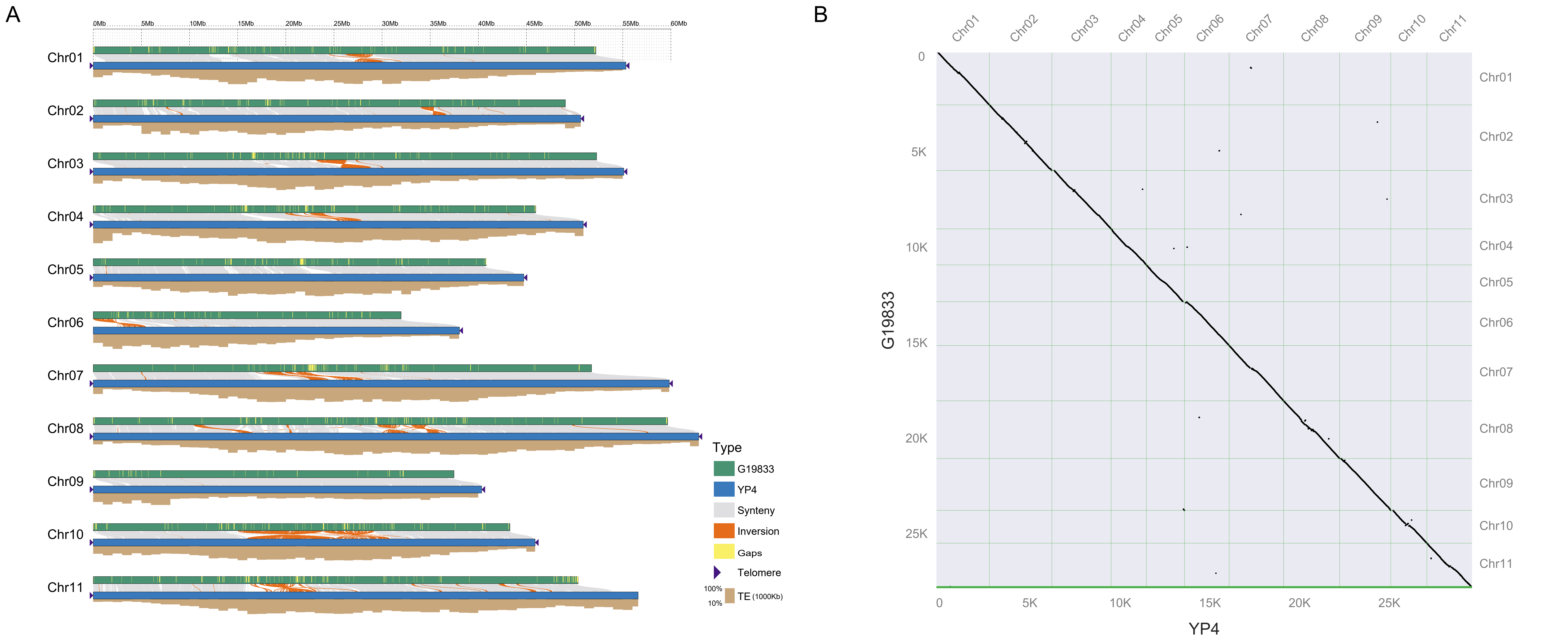

Click here to access/download
Supplementary Material
Supplementary Table.xlsx
