# Supplementary material for: Telomere-to-telomere genome of common bean (Phaseolus vulgaris L., YP4)
Source: Gigascience. 2025 May 14;14:giaf001. doi: 10.1093/gigascience/giaf001 (PMC12077395; doi:10.1093/gigascience/giaf001)
Supplement: giaf001_GIGA-D-24-00244_Revision_1 [file giaf001_giga-d-24-00244_revision_1.pdf]

GigaScience

Telomere-to-telomere genome of common bean (Phaseolus vulgaris L., YP4)

--Manuscript Draft--

**Manuscript Number:**	GIGA-D-24-00244R1
**Full Title:**	Telomere-to-telomere genome of common bean (Phaseolus vulgaris L., YP4)
**Article Type:**	Data Note
**Funding Information:**	National Natural Science Foundation of China (32241041) — Mr. Jing Wu<br>The Major Special Science and Technology Plan in Shanxi Province (202101140601027) — Mr. Haigang Wang<br>China Agriculture Research System of MOF and MARA-Food Legumes (CARS-08) — Mr. Xiaopeng Hao<br>The National Key Research and Development Program of China (2021YFD1600600) — Mr. Jianwu Chang<br>Biological Breeding Engineering of Shanxi Agricultural University (YZGC148) — Mr. Xiaopeng Hao<br>Hou Ji Laboratory in Shanxi Province (202304010930003) — Not applicable
**Abstract:**	Background<br>Common bean is a significant grain legume in human diets. However, the lack of a complete reference genome for common beans has hindered efforts to improve agronomic cultivars.<br>Findings<br>Herein, we present the first telomere-to-telomere genome assembly of common bean (Phaseolus vulgaris L., YP4) using PacBio High-Fidelity reads, ONT ultra-long sequencing, and Hi-C technologies. The assembly resulted in a genome size of 560.30 Mb with an N50 of 55.11 Mb, exhibiting high completeness and accuracy (BUSCO score: 99.5%, QV: 54.86). The sequences were anchored into eleven chromosomes, with 20 out of 22 telomeres identified, leading to the formation of nine T2T pseudomolecules. Furthermore, we identified repetitive elements accounting for 61.20% of the genome and predicted 29,925 protein-coding genes. Phylogenetic analysis suggested an estimated divergence time of approximately 11.6 MYA between P. vulgaris and V. angularis. Comparative genome analysis revealed the expanded gene families and variations between YP4 and G19833 associated with defense response.<br>Conclusions<br>The telomere-to-telomere reference genome and genomic insights presented here are crucial for future genetic studies not only in common bean but also in other legumes.
**Corresponding Author:**	yan wang<br>Shanxi Agricultural University<br>Taiyuan, CHINA
**Corresponding Author Secondary Information:**	
**Corresponding Author's Institution:**	Shanxi Agricultural University
**Corresponding Author's Secondary Institution:**	
**First Author:**	yan wang
**First Author Secondary Information:**	
**Order of Authors:**	yan wang<br>Xiaopeng Hao

Powered by Editorial Manager® and ProduXion Manager® from Aries Systems Corporation

	Chunhai Chen
	Haigang Wang
	Peng Gao
	Xukui Yang
	Xue Dong
	Huibin Qin
	Meng Li
	Sen Hou
	Jianbo Jian
	Jianwu Chang
	Jing Wu
	Zhixin Mu
**Order of Authors Secondary Information:**	
**Response to Reviewers:**	Dear editor,<br>We sincerely thank you and the reviewers for your valuable comments and instructive advice, which help us to improve and revise our manuscript (Manuscript Number: GIGA-D-24-00244, Title: Telomere-to-telomere gap-free genome of common bean (Phaseolus vulgaris L., YP4)). We have made detailed changes accordingly. Our point-by-point responses to the reviewers' comments are provided as follows for your consideration. For ease of reviewing, all the significant changes in the revised manuscript have been highlighted in red.<br>Reviewer reports:<br>Reviewer #1: This study presents a gap-free reference genome for the common bean. Although the data is good, it does not show much valuable biological insights. Maybe, the best application of the T2T assembly is for the study of centromere structure. However, the authors did not perform any analysis on the repeat unit in centromeres.<br>Answer: Thanks for your positive comments on our work. The manuscript, designed as a Data Note-type article, focuses on the analytical aspects of constructing the T2T genome rather than focusing on biological stories. It follows a format similar to the high-quality Chiridota heheva genome published in Gigascience (Pu, Zhou et al. 2024), prioritizing detailed analysis and data presentation.<br>Following your recommendations, we conducted an analysis of the centromere region, revealing conserved centromere function and 11 repeat unit sequences. Additionally, we explored YP4-specific genes to uncover valuable biological insights. Please find more details in lines 303-323 and 363-371 of the revised manuscript.<br><br>Reference：<br>Pu, Y., et al., A high-quality chromosomal genome assembly of the sea cucumber Chiridota heheva and its hydrothermal adaptation. GigaScience, 2024. 13.<br><br><br>Line 81: "It originating from" grammar error<br>Answer: Thank you for your reminder. We changed the sentence to "It originates from". Please find more details in lines 85 of the revised manuscript.<br><br>Line 234: "Notably, the contig N50 was 824-, and 2981-fold longer than earlier released genome versions" should list the two earlier version, respectively.<br>Answer: Thanks for your nice comment. These previously published genome versions are G19833 (Schmutz et al., 2014), BAT93 (Vlasova et al., 2016), and Flavert (recently published; Carrère, Mayjonade et al. 2023), and we provide detailed contig N50 values for these genomes. Please find more details in lines 250-253 of the revised manuscript.<br><br>Reference:<br>Carrère, S., et al., First whole genome assembly and annotation of a European common bean cultivar using PacBio HiFi and Iso-Seq data. Data in Brief, 2023. 48: p.

109182.
Schmutz, J., et al., A reference genome for common bean and genome-wide analysis of dual domestications. Nature Genetics, 2014. 46: p. 707 - 713.
Vlasova, A. et al. Genome and transcriptome analysis of the Mesoamerican common bean and the role of gene duplications in establishing tissue and temporal specialization of genes. in Genome biology Vol. 17 32 (2016).

Line 279: "Notably, 96.57% the RNA-seq reads" grammar error
Answer: Thank you for your reminder. We added the word “of” in the sentence. Please find more details in lines 297-298 of the revised manuscript.

Fig 3b seems strange. Does the whole pie should be cut into 3 parts? In addition, the legend "RNA-seq clean data verified the accuracy of protein-coding gene prediction" is not suitable.
Answer: Thanks for your comment. We referred to Fig 5b (see below) of the oriental armyworm genome study (Xu et al., 2023) to create Fig 3b and its legend. The pie chart in our figure comprises three segments: Exon, Intron, and Intergenic, displayed in a 3D stereoscopic format. Our primary objective is to showcase the precision of gene set prediction, underlined by the substantial proportion of transcriptome reads found in the exon region (96.57%). A similar figure was also created in the Astragalus membranaceus genome study (Fan et al., 2024).

Reference:
Fan, H., et al., Chromosome-scale genome assembly of Astragalus membranaceus using PacBio and Hi-C technologies. Scientific Data, 2024. 11(1): p. 1071.
Xu, C., et al. Chromosome level genome assembly of oriental armyworm Mythimna separata. Scientific data, 2023. 10, 597

There are lots of grammar error needs corrected. It is better to use some AI or ask a native speaker to polish the language.
Answer: Thanks for your comment. In the revised version, we initially utilized ChatGPT to improve the writing quality. Following that, we sought guidance from seasoned professors with extensive writing proficiency. We sincerely hope that these modifications comply with the publishing guidelines.

Reviewer #2: The manuscript by Wang et al., describes a telomere-to-telomere sequence assembly of common bean (P. vulgaris) and a basic annotation. Two high quality genome assemblies of common bean have been published before. One (Schmutz et al., 2014) has been mentioned, another (Carrere et al., 2023) has not been mentioned in the manuscript. The second assembly was based on SMRT reads and reported a 566.23 Mb-long chromosome scale assembly with 29,549 annotated genes and BUSCO completeness of 99.2%.
Answer: Thanks for your nice comment. In the revised article, the background section has been enhanced to incorporate the description of the common bean genome published in 2023 by Carrère, Mayjonade et al. (Carrère, Mayjonade et al. 2023). Key indicators related to this study have been summarized in Table 1. Furthermore, a recent report on the pan-genome research of the common bean by Cortinovis, Vincenzi et al. in 2024 (Cortinovis, Vincenzi et al. 2024) has been included in the background section. These updates contribute to a more comprehensive understanding of the current research landscape on the common bean genome. For further details, please refer to lines 60-62 of the revised manuscript.

Reference:
Carrère, S., et al., First whole genome assembly and annotation of a European common bean cultivar using PacBio HiFi and Iso-Seq data. Data in Brief, 2023. 48: p. 109182.
Cortinovis, G., et al., Adaptive gene loss in the common bean pan-genome during range expansion and domestication. Nature Communications, 2024. 15(1): p. 6698.

Powered by Editorial Manager® and ProduXion Manager® from Aries Systems Corporation

	The present assembly claims, it is gap-free. Actually, claiming gap-free is a big ask. It is difficult to prove. Telomere-to-telomere is a correct terminology. The present manuscript does not report any new scientific information.<br>Answer: Thank you for your reminder. The primary challenge in genome assembly is not the genome size, but the presence of repetitive sequences. In plants, centromere regions are rich in retrotransposons and tandem repeats, while telomeres consist of highly conserved tandem nucleotide repeats. In this study, we employed the latest sequencing technology and assembly algorithms to assemble the YP4 genome, successfully capturing all centromeres. Proving the quality of gap-free assemblies is difficult. Methods such as fluorescence in situ hybridization (FISH) and chromatin immunoprecipitation followed by high-throughput sequencing (ChIP-seq) are essential for validating the intricate genomic architectures and repetitive regions. These techniques will inform our future research efforts. To enhance the rigor of the article, we have removed the term "gap-free" in the revised manuscript.<br>The manuscript, intended for a Data Note-type article, emphasizes the analytical aspects of constructing the T2T genome for further study rather than focusing on biological stories. Its structure closely resembles that of the high-quality Chiridota heheva genome recently published in Gigascience (Pu, Zhou et al. 2024), prioritizing detailed analysis and data presentation.<br>To enhance the significance of the data, we identified genes within the centromeric regions and conducted GO functional enrichment analysis. The most significant GO term we discovered was "nucleic acid binding," a finding that aligns with similar observations in rice gap-free studies (Song et al., 2021). For further details, please refer to lines 303-323 of the revised manuscript.<br><br>Reference：<br>Pu, Y., et al., A high-quality chromosomal genome assembly of the sea cucumber Chiridota heheva and its hydrothermal adaptation. GigaScience, 2024. 13.<br>Song, J.-M., et al., Two gap-free reference genomes and a global view of the centromere architecture in rice. Molecular Plant, 2021. 14(10): p. 1757-1767.<br><br>Had they identified any gene or locus associated with an important trait in this assembly which has been absent in the previously published assemblies, then they could have argued the importance and better utility of these assembly. At present form of<br>the manuscript, it is just another high-quality assembly of common bean. The other analyses presented in the manuscript are routine and do not provide new insight.<br>Answer: Thanks for your nice comment. This study aims to provide the first T2T version of the common bean genome, enabling researchers to perform diverse omics analyses, including selection signal analysis of the centromere region, structural variation analysis, and pan-genomic studies. Additionally, we believe the new insights from our revised manuscript are as follows:<br>(1)We have added the characteristics of centromeres, discovering conserved centromere functions and identifying 11 repeat unit sequences.<br>(2)We conducted a comparative analysis of YP4-specific genes against G19833, indicating that YP4 may exhibit enhanced resistance and a more intricate regulatory network.<br><br>For further details, please refer to lines 303-323 and 363-371 of the revised manuscript.<br><br>With best regards,<br>Yours sincerely
**Additional Information:**	
**Question**	**Response**
Are you submitting this manuscript to a special series or article collection?	No
**Experimental design and statistics**	Yes

Powered by Editorial Manager® and ProduXion Manager® from Aries Systems Corporation

Full details of the experimental design and statistical methods used should be given in the Methods section, as detailed in our Minimum Standards Reporting Checklist. Information essential to interpreting the data presented should be made available in the figure legends.<br><br>Have you included all the information requested in your manuscript?	
**Resources**<br><br>A description of all resources used, including antibodies, cell lines, animals and software tools, with enough information to allow them to be uniquely identified, should be included in the Methods section. Authors are strongly encouraged to cite Research Resource Identifiers (RRIDs) for antibodies, model organisms and tools, where possible.<br><br>Have you included the information requested as detailed in our Minimum Standards Reporting Checklist?	Yes
**Availability of data and materials**<br><br>All datasets and code on which the conclusions of the paper rely must be either included in your submission or deposited in publicly available repositories (where available and ethically appropriate), referencing such data using a unique identifier in the references and in the “Availability of Data and Materials” section of your manuscript.<br><br>Have you have met the above requirement as detailed in our Minimum Standards Reporting Checklist?	Yes

Manuscript Click here to access/download;Manuscript;Manuscript_20241227.docx

DATANOTE

Telomere-to-telomere genome of common bean (*Phaseolus vulgaris* L., YP4)

Yan Wang[1,2†], Xiaopeng Hao[1,2,†], Chunhai Chen[3,†], Haigang Wang[1,2,†], Peng Gao[3,†], Xukui Yang[3,†], Xue Dong[1,2], Huibin Qin[1,2], Meng Li[1,2], Sen Hou[1,2], Jianbo Jian[3], Jianwu Chang[1,2], Jing Wu[4,*], Zhixin Mu[1,2,*]

[1]Center for Agricultural Genetic Resources Research, Shanxi Agricultural University, Taiyuan 030031, China

[2]Key Laboratory of Crop Gene Resources and Germplasm Enhancement on Loess Plateau, Ministry of Agriculture, Taiyuan 030031, China

[3]BGI Genomics, Shenzhen 518083, China

[4]Institute of Crop Sciences, Chinese Academy of Agricultural Sciences, Beijing 100089, China

[*]Correspondence address. Zhixin Mu, Center for Agricultural Genetic Resources Research, Shanxi Agricultural University, NO.161 Longcheng North Street, Xiaodian District Taiyuan, Shanxi Province, China. E-mail: muzx2008@sina.com; Jing Wu, Institute of Crop Sciences, Chinese Academy of Agricultural Sciences, NO.12 Zhongguancun South Street, Haidian District Beijing, China. E-mail: wujing@caas.cn.

[†] These authors contributed equally to this work.

ORCID IDs: Yan wang [0000-0002-6229-5807]; Xiaopeng Hao [0000-0002-9992-7492]; Chunhai Chen [0009-0001-7879-8716]; Haigang Wang [0009-0005-1731-8669]; Jianbo Jian [0000-0003-2187-5490].

Abstract

Background

Common bean is a significant grain legume in human diets. However, the lack of a complete reference genome for common beans has hindered efforts to improve agronomic cultivars.

Findings

Herein, we present the first telomere-to-telomere genome assembly of common bean (*Phaseolus vulgaris* L., YP4) using PacBio High-Fidelity reads, ONT ultra-long sequencing, and Hi-C technologies. The assembly resulted in a genome size of 560.30 Mb with an N50 of 55.11 Mb, exhibiting high completeness and accuracy (BUSCO score: 99.5%, QV: 54.86). The sequences were anchored into eleven chromosomes, with 20 out of 22 telomeres identified, leading to the formation of nine T2T pseudomolecules. Furthermore, we identified repetitive elements accounting for 61.20% of the genome and predicted 29,925 protein-coding genes. Phylogenetic analysis suggested an estimated divergence time of approximately 11.6 MYA between *P. vulgaris* and *V. angularis*. Comparative genome analysis revealed the expanded gene families and variations between YP4 and G19833 associated with defense response.

Conclusions

The telomere-to-telomere reference genome and genomic insights presented here are crucial for future genetic studies not only in common bean but also in other legumes.

Background

The common bean (*Phaseolus vulgaris* L., NCBI:txid3885; 2n=22) is an essential protein source that complements carbohydrate-rich foods such as rice, maize, and cassava [1]. It is globally significant as the most widely consumed legume, contributing substantially to daily caloric and protein intake, particularly in Africa and the Americas. In some regions, it accounts for up to 15%

of total daily calories and 36% of daily protein intake [2]. Over 200 million people in sub-Saharan Africa rely on it as a staple food. Furthermore, the common bean is rich in health-beneficial nutrients, and their concentrations are heritable [3, 4]. Breeding programs aim to enhance these nutrient concentrations globally [5]. As a representative of the legume family, the common bean plays a vital role in global food security and offers significant potential for further nutritional enhancement through breeding efforts.

Extensive molecular genetics research has focused on the common bean. Common bean is organized in two geographically isolated and genetically differentiated wild gene pools: the Mesoamerican gene pool and the Andean gene pool [6]. In 2014, the genome of the Mesoamerican gene pool material (G19833) was decoded, revealing a scaffold length of 521.08 Mb with a contig N50 of 39,053 [2]. In 2016, the Andean gene pool (BAT93) was sequenced, yielding a genome size of 549.60 Mb and a contig N50 of 10,795 [7]. A 2020 study utilized 4.8 million SNPs to conduct whole-genome association analysis on 20 agronomic traits, identifying over 500 genetic loci [8], providing precise markers for key traits in molecular breeding. Advancements in sequencing and assembly programs have resulted in a more contiguous common bean genome with a contig N50 size of 19.79 Mb [9]. Furthermore, a pan-genome study identified approximately 234 Mb of additional sequences containing 6,905 protein-coding genes [10]. Comparative genomic analysis revealed 376 nucleotide-binding site-leucine-rich repeat (NLR) genes in common bean, compared to 319 NLR genes in soybean [11, 12]. This discrepancy may be attributed to the stronger adaptive capacity of common bean to ecological environments, leading to the evolution of more resistance mechanisms and thus more resistance genes [7]. Besides, numerous transcriptomic studies shed light on the genetic regulation and molecular mechanisms underlying various traits in

this important crop, such as the GATA transcription factor, MADS-box gene family, and WOX gene family [13-15]. These highlight the significant interest in common beans and the importance of ongoing studies in this field.

De novo genome assembly is a crucial tool in genomics research, but it has been hindered by assembly errors, large gaps, unplaced scaffolds, and strain-specific variants [16]. However, advances in sequencing and assembly algorithms have make telomere-to-telomere (T2T) genome assembly feasible, enabling comprehensive genome identification. Currently, over 63 T2T plant assemblies have been generated [17], including several essential crops, such as rice [18], maize [19], soybean [20], and sorghum [21]. Although the common bean holds great significance in agricultural and nutritional contexts, a T2T genome assembly for this important crop has not yet been reported. This study aims to bridge this gap by integrating Pacific Biosciences (PacBio) HiFi sequencing, Oxford Nanopore Technologies (ONT) ultra-long sequencing, and chromosomal conformational capture (Hi-C) technology to assemble a T2T genome of common bean variety Pinjinyun No. 4 (YP4). YP4 is a new variety of red kidney bean that was successfully bred by Shanxi Province in 2020.It has been officially named "Jinrenyun 202001" and represents a significant addition to the agricultural biodiversity of the region. It originates from British red variety seeds irradiated by cobalt-60, belongs to the Andean center of cultivation, with a growth period of 99 days, tall stature, superior branching, and high stalk and seed yield (Fig. 1A). Its seeds are wide and plump, with a lustrous, vivid seed coat (Fig. 1B). Significantly, the average weight of 100 seeds is 51.4 grams, and the seed contains 26.4% crude protein and 54.66% starch. This variety boasts a wide range of advantages and promising prospects, making it suitable for various applications, including grain consumption, processing, export, and as a source of mature straw

for feed. The deep sequencing of the YP4 whole genome holds significant value and importance for genetic research and molecular breeding development.

Materials and methods

Sample collection

An individual plant of YP4 from Xiaodian district, Taiyuan, China (112.579° E, 23 37.778° N), was selected for sequencing. Fresh leaves harvested from this individual for genome DNA sequencing. Additionally, leaf, stem, root, flower, and pod samples were collected for RNA-sequencing (RNA-seq) to facilitate gene annotation. All samples were promptly frozen in liquid nitrogen and stored at -80°C to ensure their preservation for further analysis.

Sequencing and filtering

High-molecular-weight genomic DNA was extracted from the sample using a modified cetyltrimethylammonium bromide method [22] to facilitate subsequent library construction. For PacBio sequencing, libraries were prepared with an insert size of 15 kb using the SMRTbell Template Prep Kits from Pacific Biosciences of California, Inc. The sequencing was conducted in circular consensus sequencing mode on the PacBio Sequel II platform (RRID:SCR_017990). Subsequently, the subreads were processed using SMRTLink v8.0.0 [23] with the parameters: "-minPasses 3 -minPredictedAccuracy 0.99 -minLength 500".

For ONT sequencing, ONT ultra-long insert libraries were generated utilizing the Oxford Nanopore SQK-LSK109 kit and sequenced on the PromethION sequencer (RRID:SCR_017987).

The ONT data underwent processing using NanoFilt v2.8.020 (RRID:SCR_016966) [24] with a quality threshold of 7.

In addition, Hi-C libraries based on *Dpn*II restriction enzymes were generated for Hi-C sequencing, following previously described methods [25]. These libraries were sequenced on the MGISEQ-2000 platform, generating paired-end 150 bp reads. Clean Hi-C data were obtained using SOAPnuke v2.0 (RRID:SCR_015025) [26] with parameters set as "-n 0.01 -l 20 -q 0.1 -i -Q 2 -G 2 -M 2 -A 0.5".

For RNA-seq, libraries were constructed using the NEBNext® Ultra™ RNA Library Prep Kit for Illumina® (NEB, Ipswich, MA, USA) following the manufacturer's protocol. The libraries were then sequenced on a MGISEQ-2000 instrument, producing 150 bp paired-end reads. Quality control of the RNA-seq data was performed using fastp v0.19.5 (RRID:SCR_016962) [27] with the following parameters: "--adapter_sequence AAGTCGGAGGCCAAGCGGTCTTAGGAAGACAA --adapter_sequence_r2 AAGTCGGATCGTAGCCATGTCGTTCTGTGAGCCAAGGAGTTG --average_qual 15 -l 150".

Genome assembly and Hi-C scaffolding

The *de novo* genome assembly of YP4 comprised the four steps: primary assembly, Hi-C scaffolding, gap-filling, and optimization. At first, the primary contigs were generated via Hifiasm v 0.15.1 (RRID:SCR_021069) [28] with the recommend command "hifiasm -o YP4.asm -t32 --ul ul.fq.gz --h1 read1.fq.gz --h2 read2.fq.gz HiFi-reads.fq.gz". Subsequently, we used Bowtie2 v 2.2.9 (RRID:SCR_016368) [29] to align the Hi-C clean data to the primary contigs for anchoring contigs onto chromosomes. Low-quality reads were eliminated using the HiC-Pro pipeline (RRID:SCR_017643) [30] with default parameters. The remaining valid reads were utilized to

anchor chromosomes with Juicer v 1.6 (RID:SCR_017226) [31] and 3d-dna pipeline v 180419 (RRID:SCR_017227) [32]. Referring to the methods described in the gap-free genome of *Neosalanx taihuensis* [33], we applied the LR_Gapcloser (RRID:SCR_017021) [34] program to close gaps in the assembled chromosomes. To further enhance the genome quality, a polishing procedure described by Mc Cartney *et al*. 2022 [35] was implemented. Briefly, Winnowmap2 v 2.03 [36] was used to align the HiFi reads to the chromosomes, followed by filtering of alignments to exclude secondary alignments and those with excessive clipping using the 'falconc bam-filter-clipped' tool. Finally, racon v 1.5.0 (RRID:SCR_017642) [37] was performed with the filtered alignments.

The completeness of the assembly was evaluated utilizing Benchmarking Universal Single-Copy Orthologs (BUSCO) v 5.5.0 (RRID:SCR_015008) [38] based on the embryophyta_odb10 database (1614 orthologs). The quality value was generated by Merqury program v 1.3 (RRID:SCR_022964) [39] with 17-mer.

Genome annotations

We followed methods similar to those described by Qu *et al*. [40] for annotating repetitive sequences. Tandem Repeats Finder v 4.10 (RRID:SCR_022065) [41] was used to identify the tandem repeat elements. To detect interspersed repetitive sequences, we employed a strategy that combined *de novo* prediction and known repeat searching. RepeatModeler v 1.0.8 (RRID:SCR_015027) [42] and LTR_FINDER v 1.0.6 (RRID:SCR_015247) [43] were used to predict *de novo* repeat sequences. Subsequently, RepeatMasker v 4.0.7 (RRID:SCR_012954) [44] was applied to screen the YP4 genome against the combined *de novo* transposable element library.

Additionally, RepeatMasker v 4.0.7 (RRID:SCR_012954) [44] along with the Repbase database (RRID:SCR_021169) [45] were utilized to identify known transposable element repeats.

Similar to the method described for wild blueberry T2T assembly [46], telomeric sequences and the centromeres region in the YP4 genome assembly were identified using quartet v 1.0.3 [47] with the "-c plant" option. The telomere repeat monomer identified by TeloExplorer module in quarTeT program was "AAACCCT". To identify the repeat unit, tandem repeats ranging from 30 to 500 bp with a copy number greater than 10 were scanned within the centromeric regions. The cd-hit v 4.8.1 (RRID:SCR_007105) [48] program was utilized to cluster the candidate repeats, and the representative sequences with the highest copy number were selected as the repeat unit.

The gene prediction process involved a comprehensive approach that integrated transcriptome-based, homology-based, and *ab initio* prediction methods. Initially, RNA-seq clean reads were assembled using Trinity v 2.15.1 (RRID:SCR_013048) [49] with parameters '--max_memory 200G --CPU 40 --min_contig_length 200 --genome_guided_bam merged_sorted.bam --full_cleanup --min_kmer_cov 4 --min_glue 4 --bfly_opts '-V 5 --edge-thr=0.1 --stderr' --genome_guided_max_intron 10000'. The resulting assembled transcripts were then aligned to the assembly utilizing Program to Assemble Spliced Alignment (PASA) v 2.4.1 (RRID:SCR_014656) [50]. Gene structures were generated from valid transcript alignments (PASA-set). Additionally, RNA-seq clean reads were mapped to the assembly via Hisat2 v 2.0.1 (RRID:SCR_015530) [51]. Subsequently, Stringtie v 1.2.2 (RRID:SCR_016323) [52] and TransDecoder v 5.7.1 (RRID:SCR_017647) were employed to assemble the transcripts and identify candidate coding regions, resulting in the creation of gene models (Stringtie-set). Homologous genomes from six assemblies, including *Glycine max* (Zhonghuang 13) [53], *Glycine max* (Wm82-NJAU) [54],

Arabidopsis thaliana [55] , *Phaseolus vulgaris* L. (G19833) [2], *Vigna angularis* (ensemble release-57), and *Medicago truncatula* (ensemble release-57) were downloaded and used as queries to search against the assembly using GeMoMa v 1.9 (RRID:SCR_017646) [56]. These homology predictions were referred to as "Homology-set". For *ab initio* prediction methods, AUGUSTUS v 3.2.3 (RRID:SCR_008417) [57] was used to predict coding regions in the soft-masked genome. The gene models from these three sources were then merged using EvidenceModeler v 2.1.0 (RRID:SCR_014659) [58], with different weight parameters assigned to evidence from different sources (10 for PASA-set, 5 for Stringtie-set, 5 for Homology-set, and 1 for AUGUSTUS gene prediction). Finally, the generated gene models underwent further refinement with PASA v 2.4.1 (RRID:SCR_014656) [50] to obtain untranslated regions and alternative splicing variation information.

The integrated gene set was translated into amino-acid sequences and annotated using the method described in Zhou *et al.* [33]. Furthermore, we employed the RGAugury pipeline [59] to screen the whole gene set for resistance gene analogs (RGAs) gene prediction with a method similar to that described in the eggplant genome study [60]. The default *P*-value cutoff for initial RGAs gene filtering was set to le-5 for BLASTP.

Gene families and phylogenomic analysis

The OrthoMCL v2.0.9 (RRID:SCR_007839) [61] program, with default settings except for an inflation factor set at 1.5, was applied to determine gene families among eight plants: *A. thaliana* [55], *Cicer arietinum* (GCF_000331145), *Cajanus cajan* (GCF_000340665.1), *G. max* [54], *M. truncatula* (ensembl release-57), *Lupinus angustifolius* (ensembl release-57), *P. vulgaris* (YP4, this

study), and *V. angularis* (ensembl release-57). The input for OrthoMCL comprised the results of an all-versus-all BLASTP with an E-value cutoff of 1e-5. The outcomes of gene family clustering were summarized using UpSet (RRID:SCR_022731) [62]. A total of 1,296 single copy gene families among these species were aligned using muscle v 5.1 [63] (RRID:SCR_011812). Subsequently, the alignments were concatenated into a super alignment matrix to reconstruct the phylogenetic tree via the maximum likelihood method using iqtree2 v 2.2.0 [64] with parameters of "-m MFP -B 1000". The program MCMCtree v 4.4 in the PAML package (RRID:SCR_014932) [65] was used to estimate the divergence times among the eight species, with the JC69 nucleotide substitution model and an independent rates clock. Two standard divergence time points from the TimeTree database (RRID: SCR_021162) [66] were used for calibration: 1) *A. thaliana* - *C. cajan* 102.0 - 112.5 Mya; 2) *M. truncatula* - *C. arietinum* 24.9 - 51.0 Mya. CAFE v 4.2.1 (RRID:SCR_005983) [67] was used to measure the expansion and contraction of gene families. Based on the maximum likelihood modeling of gene gain and loss, we analyzed gene families for signs of expansion or contraction. Gene Ontology (GO) enrichment of YP4-specific genes, as well as genes in the expansion gene families, was conducted using clusterProfiler v4.2.2 (RRID:SCR_016884) [68].

Comparative genomic analysis

We performed whole genome alignment between YP4 and G19833 [2] using mummer v 4.0.0rc1 (RRID:SCR_018171) [69] with parameters: "--mum -g 1000 -c 90 -l 40". The delta-filter program was used to identify alignment blocks with the setting "-1". Subsequently, the show-snps program

was utilized to detect SNPs and insertions/deletions (InDels) with the settings "-Clr -x 1 -T". SNPs and InDels were annotated using the ANNOVAR package (RRID:SCR_012821) [70].

At the gene level, pairwise synteny search was conducted using LAST v1270 (RRID:SCR_006119). The alignment results were refined using the JCVI utility libraries in MCScan (RRID:SCR_017650) (Python version) [71] with parameter: "–cscore =0.99", followed by visualization of the syntenic regions.

The method employed in the Sorghum T2T study [72] was used to investigate the YP4-specific genes. Initially, a sliding window strategy was applied to segment the YP4 genome, with a window size of 500 bp and a step of 100 bp. Subsequently, all segmented sequences were aligned to the G19833 genome using the BWA tool v 0.7.13-r1126 (RRID:SCR_010910) [73] with the MEM algorithm (-w 500 -M -t 16). Sequences that either failed to align with the G19833 genome or exhibited less than 25% coverage were classified as YP4-specific sequences. To determine YP4-specific genes, the longest coding sequence (CDS) for each gene was extracted. Genes with over 75% of their CDS covered by these specific sequences were designated as putative YP4-specific genes.

Results

Assembly of T2T common bean reference genome for YP4

The genome assembly of YP4 utilized multiple sequencing technologies, including PacBio HiFi reads, ONT ultra-long reads, and Hi-C reads. In summary, 31.75 Gb (~56.67× coverage) of PacBio HiFi reads, 177.04 Gb of ONT ultra-long reads (~315.97× coverage), and 144.79 Gb (~258.42× coverage) of Hi-C data (Supplementary Table S1) were generated. The N50 length of the HiFi

reads exceeded 15 kb, while the N50 length of the ONT reads was over 57 kb (Supplementary Table S1; Supplementary Fig. S1; Supplementary Fig. S2). The contigs were assembled using hifiasm, resulting in 558 contigs with a total size of 606.25 Mb and an N50 length of 32.18 Mb (Supplementary Table S2). Notably, the contig N50 of our assembly was significantly longer than that of the previously published genome versions, being 1.63-, 823.96-, and 2980.83-fold longer than the Flavert (contig N50: 19.79 Mb), G19833 (contig N50: 39.05 kb), and BAT93 (contig N50: 10.80 kb) assemblies, respectively (Table 1; Supplementary Table S2). This substantial improvement establishes a solid foundation for the creation of a T2T genome assembly. Subsequently, the initial contigs served as the backbone to scaffold contigs into chromosomes with Hi-C data. Our result showed that the hifiasm assembly consisted of continuous sequences spanning the entire lengths of chromosomes 2 and 9, with 17 gaps distributed across 9 of the chromosomes (Supplementary Table S3). After gap filling and polishing, the final assembly achieved a total size of 560.30 Mb with an N50 of 55.11 Mb, comprising 11 gap-free chromosomes ranging from 38.04 to 62.89 Mb in length (Fig. 2A; Table 1).

To validate the accuracy and completeness of the YP4 T2T genome assembly, multiple approaches were employed. Firstly, the Hi-C heatmap displayed a high level of consistency across all chromosomes, providing evidence for the accurate sequencing, ordering, and orientation of contigs in the YP4 genome assembly (Fig. 2B). Secondly, 100% of ONT reads and 99.95% of HiFi reads were effectively mapped to the YP4 genome assembly, resulting in genome coverage of 99.49% and 98.90%, respectively. Furthermore, the Merqury-estimated quality value of YP4 was 54.86, confirming the high accuracy of the assembly (Table 1). Thirdly, all 11 centromeres were predicted in the YP4 genome assembly, with lengths ranging from 611,691 bp to 3,362,683 bp (Fig. 2C).

Remarkably, 20 out of the 22 telomeres were detected, leading to nine telomere-to-telomere (T2T) pseudomolecules for the entire genome (Fig. 2C; Supplementary Table S4). Finally, the Benchmarking Universal Single-Copy Orthologs (BUSCO) test indicated that the YP4 assembly successfully identified 99.5% of the 1,614 embryophyta gene set (Fig. 2D; Table 1). Overall, these findings demonstrate the high quality and reliability of the YP4 genome assembly.

Annotation of repetitive elements and protein-coding genes

Approximately 342.40 Mb of the assembled YP4 genome was classified as repetitive sequences, constituting 61.20% of the genome (Supplementary Table S5). This proportion is higher than that in G19833 (45.42%) and BAT93 (35.50%; Table 1). Among the repetitive sequences, the majority were long terminal repeats (LTRs), which comprised 36.48% of the genome. (Supplementary Table S6). The DNA, long interspersed nuclear elements (LINE), and short interspersed nuclear elements (SINE) classes accounted for 4.24%, 2.58%, and 0.11% of the genome, respectively (Supplementary Table S6).

To facilitate genome annotation of the YP4 assembly, RNA sequencing was conducted on various tissues, including root, stem, leaf, flower, and pod, yielding a total of 118.68 Gb of clean reads (Supplementary Table S7). A combined prediction strategy identified 29,925 protein-coding genes, with mean lengths of 4,042 bp for the gene, 710 bp for the intron, and 1241 bp for the coding sequence (Supplementary Table S8). The BUSCO assessment of the predicted gene sets showed 98.7% completeness with only 0.37% missing genes, indicating the robustness of the gene annotation (Supplementary Table S9). The length distribution of messenger RNA, coding sequences, exons, and introns among related species further supported the reliability of the

annotation results (Supplementary Fig. S3). Of the predicted genes, 29,426 (98.33%) carried at least one conserved functional domain (Supplementary Table S10). Additionally, 1,339 resistance gene analogs (RGAs) were identified in the YP4 assembly, surpassing the 852 RGAs found in the BAT93 genome [7] (Fig. 3A). The largest category among the RGAs was receptor-like kinases (RLKs), comprising a total of 720 genes. Notably, 96.57% of the RNA-seq reads aligned to the predicted exons (Fig. 3B). Moreover, 23,006 (78.18%) of the genes exhibited a fragments per kilobase of transcript per million mapped reads (FPKM) value above 1.0 in at least one RNA-seq sample (Supplementary Fig. S4). These results confirm the completeness and accuracy of gene prediction across the YP4 genome.

Detection of centromeres

The centromeric sequences of the 11 chromosomes were predicted, with an average length of 2.54 Mb. The longest centromeric sequence was 5.47 Mb on chromosome 7, while the shortest was 0.61 Mb on chromosome 9 (Table 2). The average repeat content in the centromeric sequences was 88.89%, significantly higher than the genome-wide repeat content of 61.20%. In most plants, centromere regions are characterized by a high abundance of retrotransposons and tandem repeats. In the case of YP4, tandem repeats comprised an average of 46.99% of the centromeric sequences, with LTR-Gypsy being the predominant interspersed repeat type (Table 2; Supplementary Table S11). A total of 400 genes overlapped with the centromeric regions, of which 377 had homologs in public databases. Gene Ontology (GO) analysis indicated that these genes were significantly enriched in seven terms, including “nucleic acid binding”, “sucrose transmembrane transporter activity”, “sucrose transport”, “DNA integration”, “zinc ion binding”, “plasma membrane”, and

“endonuclease activity” (Supplementary Fig. S5). Notably, nucleic acid binding activity was significantly enriched among the rice centromeric genes [74], while DNA integration was significantly enriched among the grapevine centromeric genes [75]. Within the centromeric regions, 11 tandem repeat units were identified (Supplementary Table S12). Among them, seven repeat units located on chromosome 1, chromosome 2, chromosome 4, chromosome 7, chromosome 8, chromosome 9, and chromosome 10 could be clustered together using cd-hit with a sequence identity threshold of 85%. To validate the authentic centromere locations, additional experiments such as fluorescence in situ hybridization and chromatin immunoprecipitation will be required.

Phylogenetic relationship analysis

The protein-coding genes of seven plant species, including *A. thaliana*, *C. arietinum*, *C. cajan*, *G. max*, *M. truncatula*, *L.angustifolius*, and *V. angularis*, were clustered into 25,888 gene families together with the protein-coding genes of YP4 (Fig. 4A). Specifically, 294 gene families containing 1,755 genes were identified as specific to YP4 when compared with the other 7 plant species (Supplementary Table S13). Among these YP4-specific genes, 1,557 (88.72%) were supported by functional annotation (Supplementary Table S14), and they were significantly enriched in 23 GO terms. The top 10 most significantly enriched GO terms included "nucleic acid binding", "zinc ion binding", "inositol catabolic process", "inositol oxygenase activity", "nutrient reservoir activity", "structural constituent of cell wall", "manganese ion transmembrane transporter activity", "cellular manganese ion homeostasis", "response to auxin", and "ribonuclease P complex" (Supplementary Fig. S6). A phylogenetic tree was constructed for the eight plant species, with *A. thaliana* serving as an outgroup (Fig. 4B). The estimated divergence time between YP4 and *V. angularis* was around

11.6 million years ago (MYA). Comparing with the most recent common ancestor (MRCA), YP4 showed 73 expansion events and 14 contraction events of each gene family (Fig. 4B). The expanded gene families of YP4 were mainly enriched in functions such as "ADP binding," "defense response," "signal transduction," "terpene synthase activity," "lyase activity," "magnesium ion binding," "manganese ion binding," "hydrolase activity, hydrolyzing O-glycosyl compounds," "phosphoric diester hydrolase activity," "carbohydrate metabolic process," and others (Supplementary Fig. S7).

Comparison of YP4 and G19833 genomes

YP4 exhibited a longer assembly length compared to G19833, with 20 telomeres assembled in YP4 but none in G19833 (Table 1; Fig. 5A). Additionally, all 40,860 gaps present in the G19833 assembly were successfully filled in the YP4 assembly, achieving complete gap closure (Table 1; Fig. 5A). The JCVI analysis showed high collinearity between YP4 and G19833 (Fig. 5B). The syntenic regions contained 23,539 orthologous pairs, with 78.66% in YP4 and 83.43% in G19833. Given that the contig N50 of G19833 was only 39,053, indicating a lack of genomic continuity in the assembly, our focus was on the variations of SNPs and InDels (2-50bp) between YP4 and G19833. A total of 1,203,386 SNPs and 317,537 InDels were detected between the two genomes (Supplementary Fig. S8). Among these variations, 44,734 (3.72%) SNPs and 3,126 (0.98%) InDels located in exonic regions (Supplementary Table S15; Supplementary Table S16). Specifically, there were 23,753 SNPs and 2,008 InDels that potentially affect gene function, associated with 6,930 genes (Supplementary Table S17). GO enrichment analysis highlighted significant enrichments in 11 terms, including "ADP binding", "defense response", "ATP binding", "protein

kinase activity", "protein phosphorylation", "protein binding", "protein serine/threonine kinase activity", "sulfotransferase activity", "oxidoreductase activity, acting on paired donors, with incorporation or reduction of molecular oxygen", "monooxygenase activity", and "recognition of pollen" (Supplementary Fig. S9). Furthermore, we identified 135 YP4-specific genes (Supplementary Table S18), which included 3 RGAs and 11 transcription factors. These YP4-specific genes were associated with various biological processes, such as “DNA binding”, “DNA repair”, “base-excision repair”, “regulation of RNA metabolic process”, and “positive regulation of DNA-binding transcription factor activity” (Supplementary Table S19). This indicated that YP4 possesses stronger resistance and a more intricate regulatory network compared to G19833. Notably, RNA-seq analysis revealed that 84 of these genes exhibited expression levels of FPKM $\geqslant$ 1, providing further evidence for their functional significance.

Conclusions

The first T2T genome assembly of a typical common bean, YP4, was successfully accomplished using PacBio HiFi reads, ONT ultra-long sequencing, and Hi-C technologies. This assembly is notable for its exceptional completeness and accuracy. A total of 11 chromosomes were assembled, with 9 chromosomes meeting the telomere-to-telomere standard. Furthermore, the assembly predicted 342.40 Mb of repetitive sequences and identified 29,925 protein-coding genes. Evolutionary analysis suggests that investigating defense responses may be a promising avenue for understanding the genetic characteristics of common beans, further supported by comparative genomics analysis. Overall, this dataset provides a valuable resource for future genetic breeding research in common beans.

Data Availability

The genome assembly and all the sequencing data have been deposited in NCBI under the accession number BioProject PRJNA1072282. All additional supporting data are available in the *GigaScience* repository, GigaDB [76].

Figure

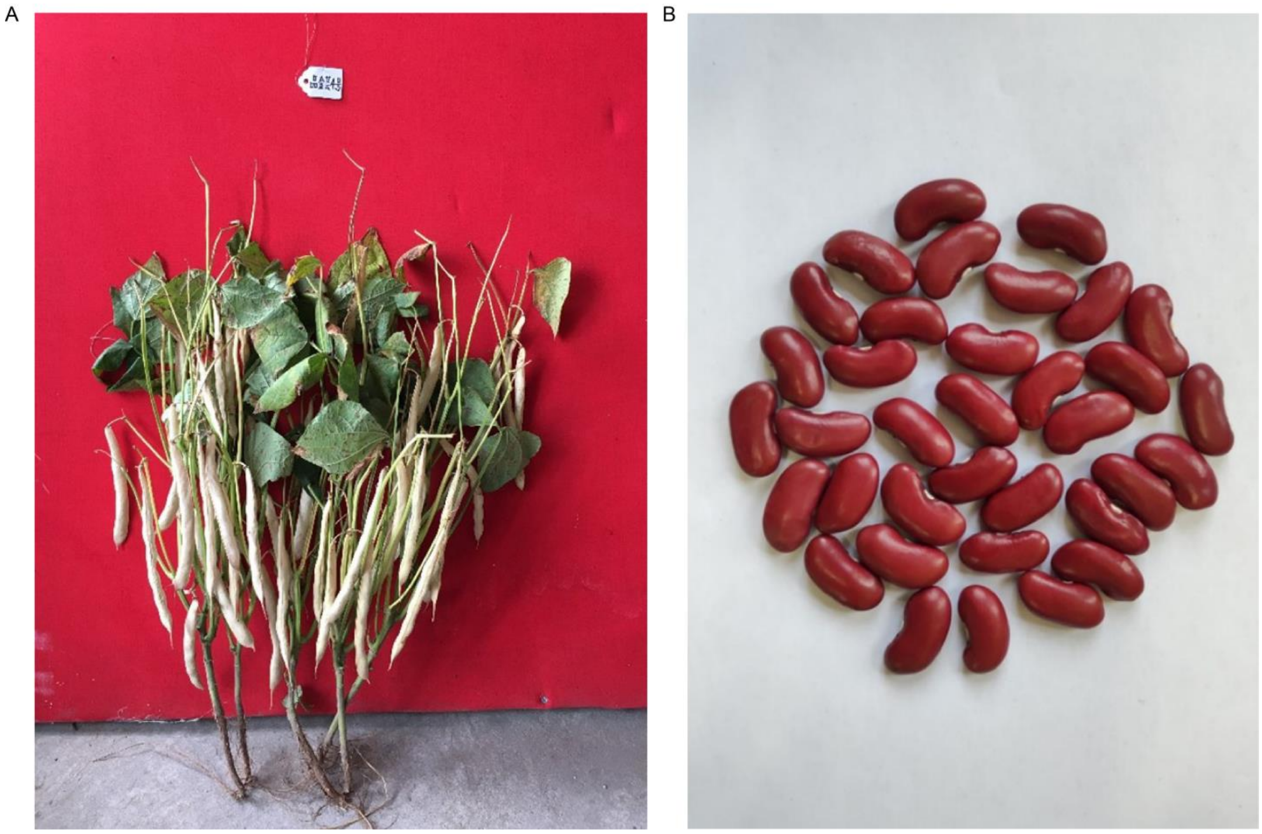

Fig. 1 | The YP4 plant sequenced in this study.A, The plant of YP4. **B**, The beans of YP4.

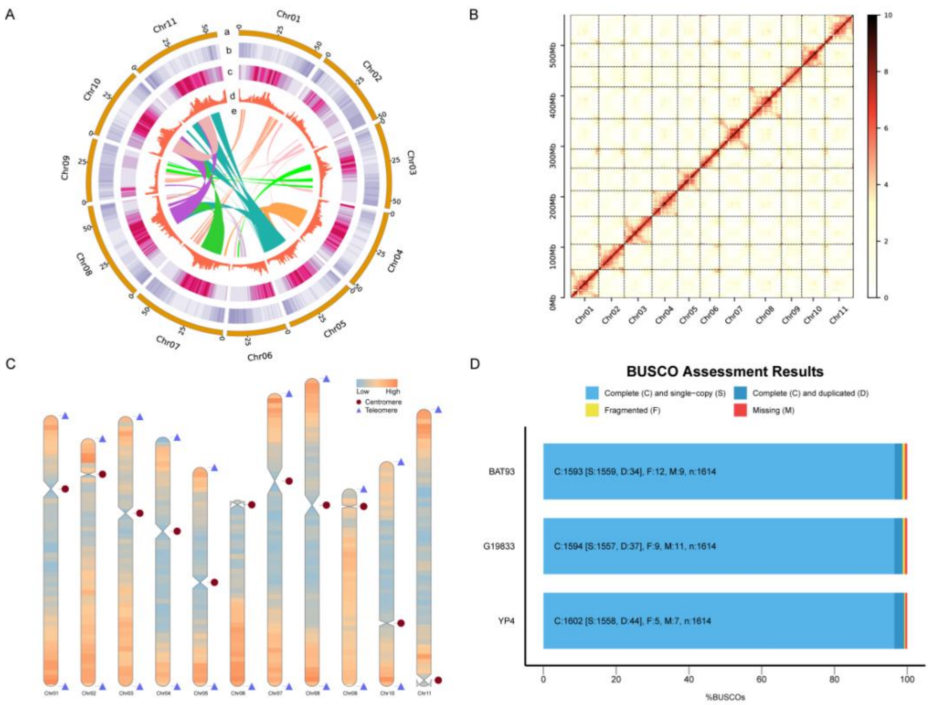

Fig. 2 | High-quality reference of YP4 genome. A, Circos plot showing the characterization of YP4 genome. From outside to inside: a, The length of pseudo-chromosome in the size of Mb. b, gene density in 1-Mb sliding windows. c, percentage of repetitive elements in 1 Mb sliding windows. d, GC content in non-overlapping 1Mb windows. e, collinear regions within the YP4 assembly. **B**, Heatmap displaying Hi-C interactions of YP4 pseudomolecules. Chr01 - Chr11 are the abbreviations of 11 Chromosome. The abscissa and ordinate represent the order of each bin on the corresponding chromosome group. The colour block illuminates the intensity of interaction from yellow (low) to red (high). **C**, Telomere and centromere detection map. Triangles and circles represent telomeres and centromere within the YP4 assembled chromosomes. The orange color represents regions with high gene density, while the sky blue color represents regions with low gene density. **D**, BUSCO assessments of the YP4, G19833, and BAT93 genome.

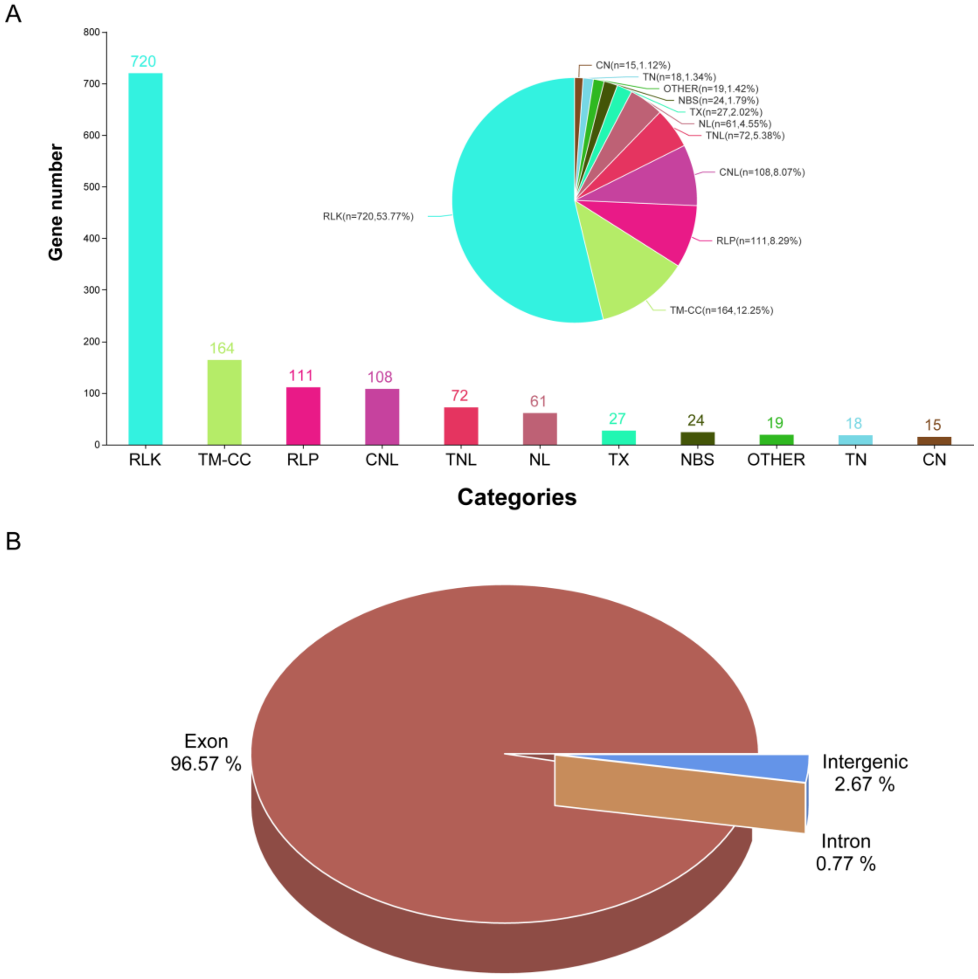

Fig. 3 | The gene annotation of YP4 assembly. A, Summary of RGAs categories in YP4 assembly. RLK, receptor-like kinase; TM, transmembrane; CC, coiled-coil; RLP, receptor-like protein; CNL, CC-NBS-LRR; TNL, TIR-NBS-LRR; NL, NBS-LRR; TX, TIR-unknown domain; NBS, nucleotide-binding site; TN, TIR-NBS; CN, CC-NBS. **B,** RNA-seq clean data verified the accuracy of protein-coding gene prediction.

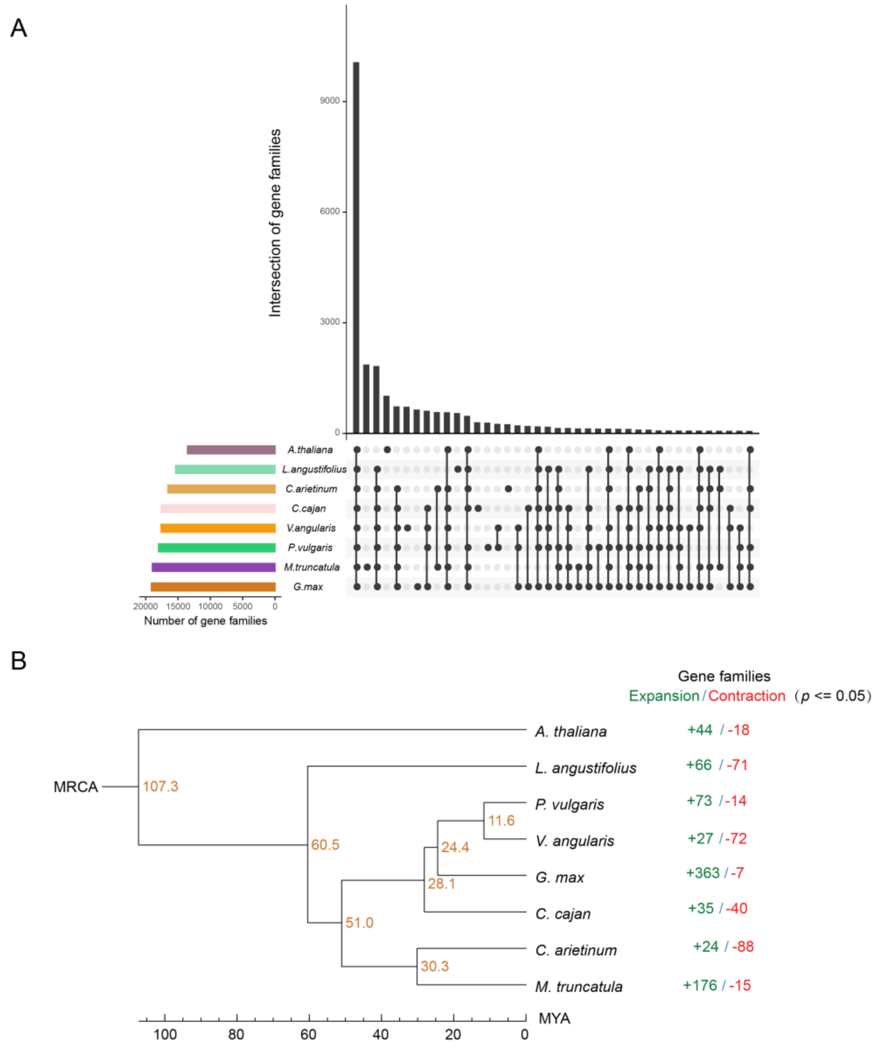

Fig. 4 | Evolution of the YP4 genome. A, UpSetPlot representing the intersections of gene families among the 8 species. Rows and columns represent gene families and intersections, respectively. Black and gray circles indicate the existence or absence of a given intersection. The horizontal bar chart on the left side of the matrix indicates the size of gene family. **B,** Phylogenetic tree of the 8 species. Numbers on nodes indicate the differentiation time. MRCA, most recent common ancestor.

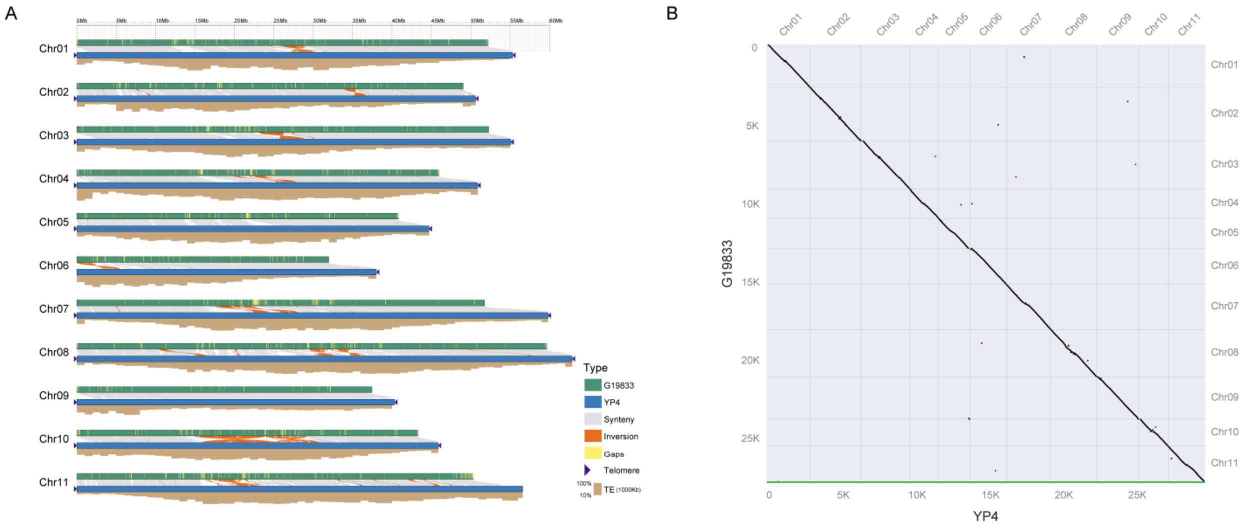

Fig. 5 | Genomic comparision between YP4 and G19833. A, Collinearity between YP4 and G19833. Gray lines illustrate collinear regions between YP4 and G19833. Triangles denote the presence of telomere sequence repeats in YP4. The yellow bar indicates gap regions in G19833. **B,** Dot-plot alignment between YP4 and G19833.

Abbreviations

BLAST: Basic Local Alignment Search Tool; BUSCO: Benchmarking Universal Single-Copy Orthologs; Gb: gigabase pairs; GO: Gene Ontology; Hi-C: High-Throughput Chromosome Conformation Capture; HiFi: High-Fidelity; KEGG: Kyoto Encyclopedia of Genes and Genomes; InDels: insertions/deletions; LINE: long interspersed nuclear element; LTR: long terminal repeat; Mb: megabase pairs; MRCA: most recent common ancestor; MYA: million years ago; RGAs: resistance gene analogs; RNA-seq: RNA sequencing; PASA: Program to Assemble Spliced Alignments; SMRT: Single-Molecule Real-time Sequencing; SNPs: single nucleotide polymorphisms; T2T: telomere-to-telomere

Additional Files

Supplementary Table S1. Summary of whole genome sequencing data of YP4 genome.

Supplementary Table S2. The statistics of the hifiasm assembly.

Supplementary Table S3. The statistics of the anchored chromosome length.

Supplementary Table S4. The identified telomeres in YP4 assembly.

Supplementary Table S5. General statistics of repeats in the YP4 assembly.

Supplementary Table S6. The summary of interspersed repeat contents in YP4 assembly.

Supplementary Table S7. The summary of RNAseq sequencing quality.

Supplementary Table S8. Summary of gene structure prediction in YP4 genome.

Supplementary Table S9. BUSCOs analysis of YP4 gene set completeness.

Supplementary Table S10. Number of functional annotations for predicted genes in the YP4 assembly.

Supplementary Table S11. Statistics on the main types of interspersed repeats within the centromere.

Supplementary Table S12. Tandem repeat unit sequence within the centromere.

Supplementary Table S13. Gene families in YP4 and other species.

Supplementary Table S14. The list of YP4 specific genes.

Supplementary Table S15. The annotation of SNPs between YP4 and G19833.

Supplementary Table S16. The annotation of Indels between YP4 and G19833.

Supplementary Table S17. The gene list impacted by variation between YP4 and G19833.

Supplementary Table S18. The YP4-specific gene list.

Supplementary Table S19. GO enrichment of YP4-specific gene.

Funding

This study is supported by National Natural Science Foundation of China (32241041), The Major Special Science and Technology Plan in Shanxi Province (202101140601027), China Agriculture Research System of MOF and MARA-Food Legumes (CARS-08), The National Key Research and Development Program of China (2021YFD1600600), Biological Breeding Engineering of Shanxi Agricultural University (YZGC148), Hou Ji Laboratory in Shanxi Province (202304010930003).

Ethical Approval

No ethical issues were involved in this study.

Competing Interests

The authors declare that they have no competing interests.

Authors' Contribution

Z. X.M., J.W.C., J.W. and H.G.W. conceived and developed the research; Y.W. and X.P.H. collected the samples, conducted experiments and analyzed the data; C.H.C, P.G. and X.K.Y. performed the data analysis; Y.W., X.P.H. and C.H.C. wrote the first draft of the manuscript; X.D., M.L., H.B.Q and S.H. participated in the research discussions and provided comments to improve the manuscript.

Acknowledgements

We thank every project that provides funding and material support for the study. We also thank each author for their ideas and skills in study design, experimentation, data collection, data analysis and manuscript writing. We sincerely thank the editors and reviewers for their valuable suggestions and comments on this study.

References

1. Graham PH,Vance CP. Legumes: importance and constraints to greater use. Plant Physiol 2003;131(3):872-77. http://doi.org/10.1104/pp.017004.
2. Schmutz J,McClean PE,Mamidi S, et al. A reference genome for common bean and genome-wide analysis of dual domestications. Nat Genet 2014;46(7):707-13. http://doi.org/10.1038/ng.3008.
3. Geil PB,Anderson JW. Nutrition and health implications of dry beans:a review. J Am Coll Nutr 1994 ;13(6):549-58. http://doi.org/10.1080/07315724.1994.10718446.
4. Cichy KA,Caldas GV,Snapp SS,et al. QTL Analysis of Seed Iron, Zinc, and Phosphorus Levels in an Andean Bean Population. Crop Science 2009;49(5):1742-50. http://doi.org/10.2135/cropsci2008.10.0605.
5. Beebe S. Common bean breeding in the tropics. Plant Breeding Reviews 2012;36:357-426. http://doi.org/10.1002/9781118358566.ch5.
6. Mamidi S,Rossi M,Moghaddam SM,et al. Demographic factors shaped diversity in the two gene pools of wild common bean *Phaseolus vulgaris* L. Heredity (Edinb) 2013;110(3):267-76. http://doi.org/10.1038/hdy.2012.82.
7. Vlasova A,Capella-Gutiérrez S,Rendón-Anaya M,et al. Genome and transcriptome analysis of the Mesoamerican common bean and the role of gene duplications in establishing tissue and temporal specialization of genes. Genome Biol 2016;17:32. http://doi.org/10.1186/s13059-016-0883-6.
8. Wu J,Wang LF,Fu JJ,et al. Resequencing of 683 common bean genotypes identifies yield component trait associations across a north-south cline. Nat Genet 2020;52(1):118-25. http://doi.org/10.1038/s41588-019-0546-0.
9. Carrère S,Mayjonade B,Lalanne D,et al. First whole genome assembly and annotation of a European common bean cultivar using PacBio HiFi and Iso-Seq data. Data Brief 2023;48:109182. http://doi.org/10.1016/j.dib.2023.109182.
10. Cortinovis G,Vincenzi L,Anderson R,et al. Adaptive gene loss in the common bean pan-genome during range expansion and domestication. Nat Commun 2024;15(1):6698. http://doi.org/10.1038/s41467-024-51032-2.
11. Meziadi C,Richard MMS,Derquennes A,et al. Development of molecular markers linked to disease resistance genes in common bean based on whole genome sequence. Plant Sci 2016;242:351-57. http://doi.org/10.1016/j.plantsci.2015.09.006.
12. Kang YJ,Kim KH,Shim S,et al. Genome-wide mapping of NBS-LRR genes and their association with disease resistance in soybean. BMC Plant Biol 2012;12:139. http://doi.org/10.1186/1471-2229-12-139.
13 . Abdulla MF,Mostafa K,Aydin A,et al. GATA transcription factor in common bean: a comprehensive genome-wide functional characterization, identification, and abiotic stress response evaluation. Plant Mol Biol 2024;114(3):43. http://doi.org/10.1007/s11103-024-01443-y.
14. Okay A,Kırlıoğlu T,Durdu YŞ,et al. Omics approaches to understand the MADS-box gene family in common

bean (*Phaseolus vulgaris* L.) against drought stress. Protoplasma 2024;261(4):709-24. http://doi.org/10.1007/s00709-024-01928-z.

15. Akbulut SE, Okay E,Aksoy T,et al. The genome-wide characterization of *WOX* gene family in *Phaseolus vulgaris* L.during salt stress. Physiol Mol Biol Plants 2022;28(6):1297-1309. http://doi.org/10.1007/s12298-022-01208-1.
16. Payne ZL,Penny GM,Turner TN,et al. A gap-free genome assembly of Chlamydomonas reinhardtii and detection of translocations induced by CRISPR-mediated mutagenesis. Plant Commun 2023;4(2):100493. http://doi.org/10.1016/j.xplc.2022.100493.
17. Xie LJ,Gong XJ,Yang K,et al. Technology-enabled great leap in deciphering plant genomes. Nat Plants 2024;10(4):551-66. http://doi.org/10.1038/s41477-024-01655-6.
18. Shang LG,He WC,Wang TY,et al. A complete assembly of the rice Nipponbare reference genome. Mol Plant 2023;16(8):1232-36. http://doi.org/10.1016/j.molp.2023.08.003.
19. Chen J,Wang ZJ,Tan KW,et al. A complete telomere-to-telomere assembly of the maize genome. Nat Genets 2023;55(7):1221-31. http://doi.org/10.1038/s41588-023-01419-6.
20. Huang YC,Koo D,Mao YZ,et al. A complete reference genome for the soybean cv. Jack. Plant Commun 2024:5(2):100765. http://doi.org/10.1016/j.xplc.2023.100765.
21. Ding YQ,Wang YL,Xu JX,et al. Short Communication A telomere-to-telomere genome assembly of Hongyingzi, a sorghum cultivar used for Chinese Baijiu production. The Crop Journal 2024;12(2):635-40. http://doi.org/10.1016/j.cj.2024.02.011.
22. Porebski S,Bailey LG,Baum BR. Modification of a CTAB DNA extraction protocol for plants containing high polysaccharide and polyphenol components. Plant Molecular Biology Reporter 1997;15:8-15. http://doi.org/10.1007/BF02772108.
23. Chin CS,Alexander DH,Marks P,et al. Nonhybrid, finished microbial genome assemblies from long-read SMRT sequencing data. Nat Methods 2013;10(6):563-69. http://doi.org/10.1038/nmeth.2474.
24. De Coster W,D'Her S,Schultz DT,et al. NanoPack: visualizing and processing long-read sequencing data. Bioinformatics 2018;34(15):2666 - 69. http://doi.org/10.1093/bioinformatics/bty149.
25. Belton JM,McCord RP,Gibcus JH, et al. Hi-C:a comprehensive technique to capture the conformation of genomes. Methods 2012;58(3):268-76. http://doi.org/10.1016/j.ymeth.2012.05.001.
26. Chen YX, Chen YS,Shi CM,et al. SOAPnuke: a MapReduce acceleration-supported software for integrated quality control and preprocessing of high-throughput sequencing data. GigaScience 2017;7(1):1-6. http://doi.org/10.1093/gigascience/gix120.
27. Chen SF,Zhou YQ,Chen YR,et al. fastp: an ultra-fast all-in-one FASTQ preprocessor. Bioinformatics 2018; 34(17):i884-90. http://doi.org/10.1093/bioinformatics/bty560.
28. Cheng HY,Concepcion GT,Feng XW,et al. Haplotype-resolved de novo assembly using phased assembly graphs with hifiasm. Nat Methods 2021;18(2):170-75. http://doi.org/10.1038/s41592-020-01056-5.
29. Langmead B,Salzberg SL,Fast gapped-read alignment with Bowtie 2. Nat Methods 2012;9(4):357-59. http://doi.org/10.1038/nmeth.1923.
30. Servant N,Varoquaux N,Lajoie BR,et al. HiC-Pro:an optimized and flexible pipeline for Hi-C data processing. Genome Biol 2015;16:259. http://doi.org/10.1186/s13059-015-0831-x.
31. Durand N,Shamim MS,Machol I,et al. Juicer provides a one-click system for analyzing loop-resolution Hi-C experiments. Cell Syst 2016;3(1):95-98. http://doi.org/10.1016/j.cels.2016.07.002.
32. Dudchenko O,Batra SS,Omer AD,et al. De novo assembly of the *Aedes aegypti* genome using Hi-C yields chromosome-length scaffolds. Science 2017;356(6333):92-95. http://doi.org/10.1126/science.aal3327.
33. Zhou YF,Zhang XZ,Jian JB,et al. Gap-free genome assembly of Salangid icefish Neosalanx taihuensis. Sci data 2023;10(1):768. http://doi.org/10.1038/s41597-023-02677-z.

34. Xu GC,Xu TJ,Zhu R,et al. LR_Gapcloser:a tiling path-based gap closer that uses long reads to complete genome assembly. GigaScience 2019;8(1):giy157. http://doi.org/10.1093/gigascience/giy157.
35. Mc Cartney AM,Shafin K,Alonge M,et al. Chasing perfection:validation and polishing strategies for telomere-to-telomere genome assemblies. Nat Methods 2022;19(6):687-95. http://doi.org/10.1038/s41592-022-01440-3.
36. Jain C,Rhie A,Hansen NF,et al. Long-read mapping to repetitive reference sequences using Winnowmap2. Nat Methods 2022;19(6):705-10. http://doi.org/10.1038/s41592-022-01457-8.
37. Vaser R,Sović I,Nagarajan N,et al. Fast and accurate de novo genome assembly from long uncorrected reads. Genome Res 2017;27(5):737-46. http://doi.org/10.1101/gr.214270.116.
38. Seppey M,Manni M,Zdobnov EM. BUSCO:Assessing genome assembly and annotation completeness. Methods Mol Biol 2019;1962:227-45. http://doi.org/10.1007/978-1-4939-9173-0_14.
39. Rhie A,Walenz BP,Koren S,et al. Merqury: reference-free quality, completeness, and phasing assessment for genome assemblies. Genome Biol 2020;21(1):245. http://doi.org/10.1186/s13059-020-02134-9.
40. Qu CM,Zhu MC,Hu R,et al. Comparative genomic analyses reveal the genetic basis of the yellow-seed trait in Brassica napus. Nat Commun 2023;14(1):5194. http://doi.org/10.1038/s41467-023-40838-1.
41. Benson G. Tandem repeats finder: a program to analyze DNA sequences. Nucleic Acids Res 1999;27(2): 573-80. http://doi.org/10.1093/nar/27.2.573.
42. Flynn JM,Hubley R,Goubert C,et al. RepeatModeler2 for automated genomic discovery of transposable element families. Proc Nati Acad Sci U S A 2020;117(17):9451-57. http://doi.org/10.1073/pnas.1921046117.
43. Xu Z,Wang H. LTR_FINDER:an efficient tool for the prediction of full-length LTR retrotransposons. Nucleic Acids Res 2007;35(Web Server issue):W265-68. http://doi.org/10.1093/nar/gkm286.
44. Chen N. Using RepeatMasker to identify repetitive elements in genomic sequences. Curr Protoc Bioinformatics 2004;Chapter 4:Unit 4.10. http://doi.org/10.1002/0471250953.bi0410s05.
45. Bao W,Kojima KK,Kohany O. Repbase Update, a database of repetitive elements in eukaryotic genomes. Mob DNA 2015;6:11. http://doi.org/10.1186/s13100-015-0041-9.
46. Zeng T,He ZJ,He JF,et al. The telomere-to-telomere gap-free reference genome of wild blueberry (*Vaccinium duclouxii)* provides its high soluble sugar and anthocyanin accumulation. Hortic Res 2023;10(11):uhad209. http://doi.org/10.1093/hr/uhad209.
47. Lin YZ,Ye C,Li XZ, et al.quarTeT:a telomere-to-telomere toolkit for gap-free genome assembly and centromeric repeat identification. Hortic Res 2023;10(8):uhad127. http://doi.org/10.1093/hr/uhad127.
48. Li WZ,Godzik A. Cd-hit: a fast program for clustering and comparing large sets of protein or nucleotide sequences. Bioinformatics 2006;22(13):1658-59. http://doi.org/10.1093/bioinformatics/btl158.
49. Grabherr MG, Haas BJ,Yassour M,et al. Full-length transcriptome assembly from RNA-Seq data without a reference genome. Nat Biotechnol 2011;29(7):644-52.http://doi.org/10.1038/nbt.1883.
50. Haas BJ,Delcher AL,Mount SM,et al. Improving the Arabidopsis genome annotation using maximal transcript alignment assemblies. Nucleic Acids Re 2003;31(19):5654-66. http://doi.org/10.1093/nar/gkg770.
51. Kim D,Langmead B,Salzberg SL. HISAT:a fast spliced aligner with low memory requirements. Nat Methods 2015;12(4):357-360. http://doi.org/10.1038/nmeth.3317.
52. Kovaka S,Zimin AV,Pertea GM,et al. Transcriptome assembly from long-read RNA-seq alignments with StringTie2. Genome Biol 2019;20(1):278. http://doi.org/10.1186/s13059-019-1910-1.
53. Zhang AQ,Kong TC,Sun BQ,et al. A telomere-to-telomere genome assembly of Zhonghuang 13, a widely-grown soybean variety from the original center of *Glycine max*. The Crop Journal 2024;12(1):142-53. http://doi.org/10.1016/j.cj.2023.10.003.
54. Wang LF,Zhang MZ,Li MN,et al. A telomere-to-telomere gap-free assembly of soybean genome. Mol Plant 2023;16(11):1711-14. http://doi.org/10.1016/j.molp.2023.08.012.

55. Hou XR,Wang DP,Cheng ZK,et al A near-complete assembly of an Arabidopsis thaliana genome. Mol Plant 2022;15(8):1247-50. http://doi.org/10.1016/j.molp.2022.05.014.
56. Keilwagen J,Hartung F,Grau J. GeMoMa:Homology-Based gene prediction utilizing intron position conservation and RNA-seq data. Methods Mol Biol 2019;1962:161-77. http://doi.org/10.1007/978-1-4939-9173-0_9.
57. Stanke M,Morgenstern B. AUGUSTUS:a web server for gene prediction in eukaryotes that allows user-defined constraints. Nucleic Acids Res 2005;33(Web Server issue):W465-67. http://doi.org/10.1093/nar/gki458.
58. Haas BJ,Salzberg SL,Zhu W,et al. Automated eukaryotic gene structure annotation using EVidenceModeler and the program to assemble spliced alignments. Genome Biol 2008;9(1):R7. http://doi.org/10.1186/gb-2008-9-1-r7.
59. Li PC,Quan XD,Jia GF,et al. RGAugury:a pipeline for genome-wide prediction of resistance gene analogs (RGAs) in plants. BMC Genomics 2016;17(1):852. http://doi.org/10.1186/s12864-016-3197-x.
60. Li DD,Qian J,Li WL,et al. A high-quality genome assembly of the eggplant provides insights into the molecular basis of disease resistance and chlorogenic acid synthesis. Mol Ecol Resour 2021;21(4):1274-86. http://doi.org/10.1111/1755-0998.13321.
61. Li L,Stoeckert Jr CJ,Roos DS. OrthoMCL:identification of ortholog groups for eukaryotic genomes. Genome Res 2003;13(9):2178-89. http://doi.org/10.1101/gr.1224503.
62. Lex A,Gehlenborg N,Strobelt H,et al. UpSet: visualization of intersecting sets. IEEE Trans Vis Comput Graph 2014;20(12):1983-92. http://doi.org/10.1109/TVCG.2014.2346248.
63. Edgar RC. Muscle5: High-accuracy alignment ensembles enable unbiased assessments of sequence homology and phylogeny. Nat Commun 2022;13(1):6968. http://doi.org/10.1038/s41467-022-34630-w.
64. Minh BQ,Schmidt HA,Chernomor O,et al. IQ-TREE 2: new models and efficient methods for phylogenetic inference in the genomic era. Mol Biol Evol 2019;37(5):1530-34. http://doi.org/10.1093/molbev/msaa015.
65. Yang Z. PAML:a program package for phylogenetic analysis by maximum likelihood. Comput Appl Biosci 1997;13(5):555-56. http://doi.org/10.1093/bioinformatics/13.5.555.
66. Hedges SB,Dudley JT,Kumar S. TimeTree:a public knowledge-base of divergence times among organisms. Bioinformatics 2006;22(23):2971-72. http://doi.org/10.1093/bioinformatics/btl505.
67. Bie TD,Cristianini N,Demuth JP,et al. CAFE:a computational tool for the study of gene family evolution. Bioinformatics 2006;22(10):1269-71. http://doi.org/10.1093/bioinformatics/btl097.
68. Wu TZ,Hu EQ,Xu SB,et al. ClusterProfiler 4.0: a universal enrichment tool for interpreting omics data. Innovation(Camb) 2021;2(3):100141. http://doi.org/10.1016/j.xinn.2021.100141.
69. Marçais G,Delcher AL,Phillippy AM,et al. MUMmer4:a fast and versatile genome alignment system. PLoS Comput Biol 2018;14(1):e1005944. http://doi.org/10.1371/journal.pcbi.1005944.
70. Wang K, Li MY, Hakonarson HH. ANNOVAR:functional annotation of genetic variants from high-throughput sequencing data. Nucleic Acids Res 2010;38(16):e164. http://doi.org/10.1093/nar/gkq603.
71. Tang HB,Bowers JE,Wang XY,et al. Synteny and Collinearity in plant genomes. Science 2008;320(5875): 488-88. http://doi.org/10.1126/science.1153917.
72. Li M, Chen CH, Wang HG,et al. Telomere-to-telomere genome assembly of sorghum. Sci Data 2024;11(1):835. http://doi.org/10.1038/s41597-024-03664-8.
73. Li H. Aligning sequence reads, clone sequences and assembly contigs with BWA-MEM. arXiv: Genomics 2013. http://doi.org/10.48550/arXiv.1303.3997.
74. Song JM, Xie WZ,Wang S,et al. Two gap-free reference genomes and a global view of the centromere architecture in rice. Mol Plant 2021;14(10):1757-67. http://doi.org/10.1016/j.molp.2021.06.018
75. Shi XY, Cao S, Wang X,et al. The complete reference genome for grapevine (*Vitis vinifera* L.) genetics and

breeding. Hortic Res 2023;10(5):uhad061. http://doi.org/10.1093/hr/uhad061.
76. Wang Y, Hao X, Chen C, et al. Supporting data for "Telomere-to-telomere genome of common bean (Phaseolus vulgaris L., YP4)" GigaScience Database. 2024. https://doi.org/10.5524/102636

Click here to access/download;Table;Table 1. Comparison of three common bean assemblies.xlsx

Table 1. Comparison of three common bean assemblies

Assembly feature	YP4	*G19833	†BAT93
Size of assembly	560,297,700	521,076,696	549,748,340
Contig N50	55,110,595	39,053	10,795
Scaffold N50	55,110,595	50,367,376	39,037,607
Longest scaffold	62,894,056	59,662,532	50,710,336
Number of gaps	0	40,860	45,300
Number of protein-coding genes	29,925	28,134	30,491
Repetitive elements	61.20%	45.42%	35.50%
Quality value	54.86	-	-
Complete BUSCOs (N=1,614)	99.50%	99.40%	99.40%

*G19833 from Ensembl database (release-56)

†BAT93 from NCBI database under accession GCA_001517995.1

[#]Flavert from NCBI database under accession number GCA_029448765.1

[#]**Flavert**
615,703,893
19,791,875
54,932,568
63,359,058
34
29,549
-
-
99.19%

Click here to access/download;Table;Table 2. The characteristic of centromeres in YP4 assembly.xlsx

Table 2. The characteristic of centromeres in YP4 assembly.

Chromosomes	Start	End	Centro length	Repeat content (%)
Chr01	13,328,531	16,691,213	3,362,683	98.20
Chr02	6,817,424	7,789,605	972,182	89.28
Chr03	18,427,633	21,069,777	2,642,145	96.82
Chr04	17,673,363	20,755,122	3,081,760	95.35
Chr05	22,263,001	24,704,779	2,441,779	99.22
Chr06	227,300	1,666,213	1,438,914	66.82
Chr07	15,175,230	20,645,715	5,470,486	92.31
Chr08	23,748,882	28,008,021	4,259,140	93.89
Chr09	3,289,103	3,900,793	611,691	89.63
Chr10	32,420,713	33,554,620	1,133,908	88.27
Chr11	54,078,975	56,597,072	2,518,098	68.01

Tandem repeats content (%)	LTR-Gypsy (%)	Gene number
47.42	46.76	23
69.75	9.22	28
31.08	60.78	20
38.33	48.26	22
61.46	38.69	10
37.00	19.35	39
31.18	48.27	77
30.97	53.06	55
71.30	10.17	10
40.52	30.39	25
57.84	0.74	91

Click here to access/download;Figure;Figure1 The YP4 plant sequenced in this study .pdf

A B

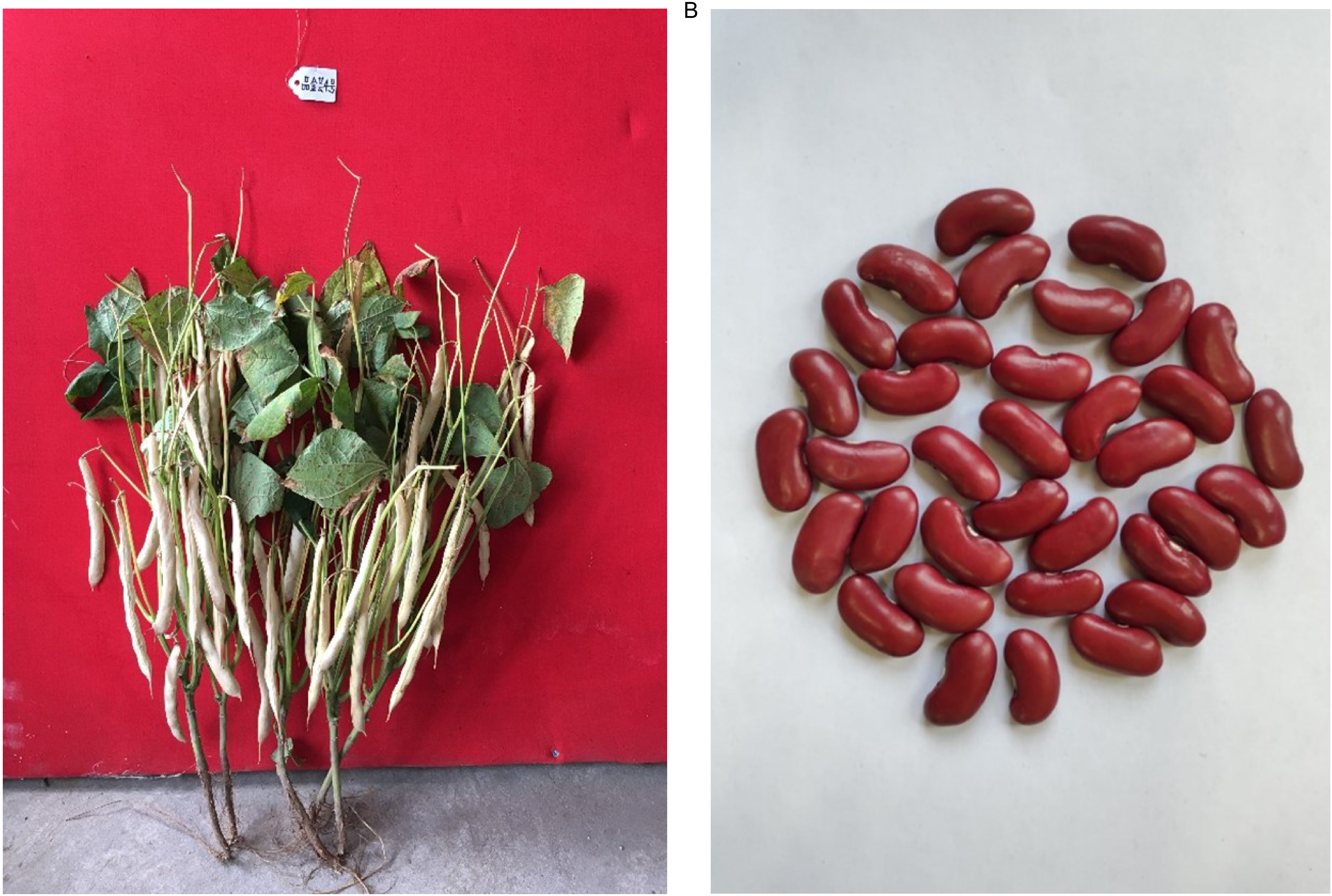

Figure2

Click here to access/download;Figure;Figure2 High-quality reference of YP4 genome.pdf

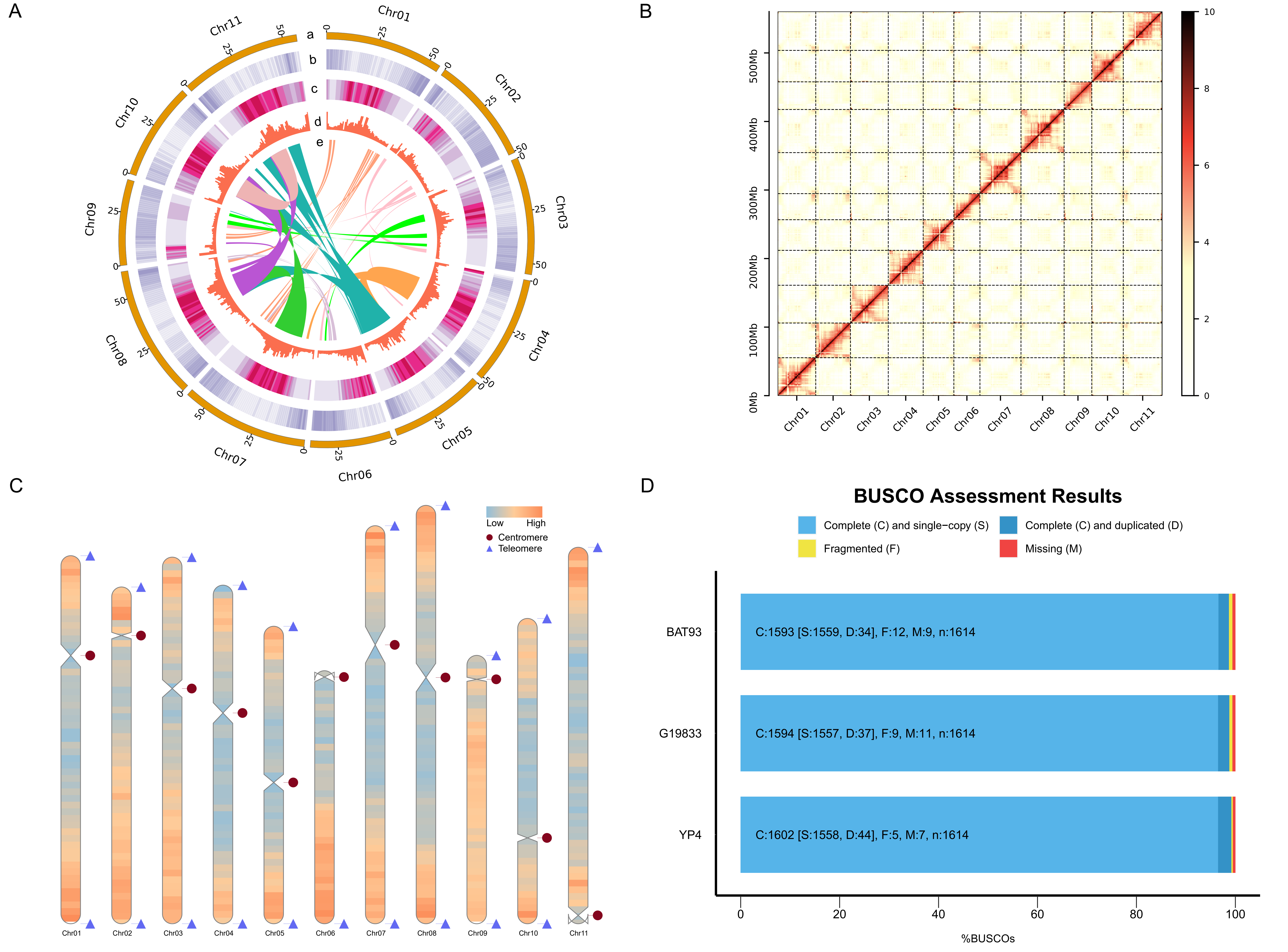

Figure3

Click here to access/download;Figure;Figure3 The gene annotation of YP4 assembly.pdf

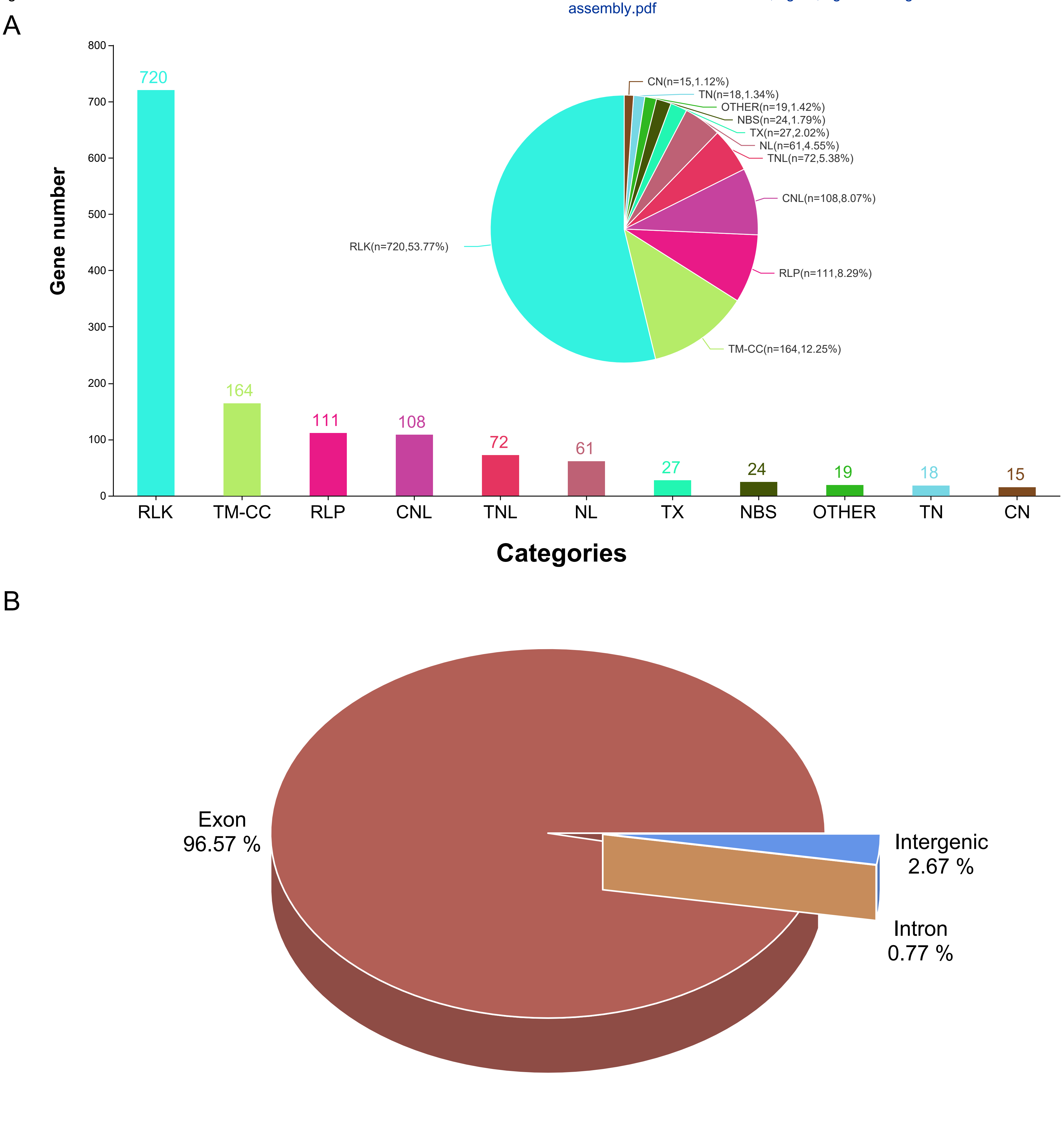

Figure4

Click here to access/download;Figure;Figure4 Evolution of the YP4 genome.pdf

A

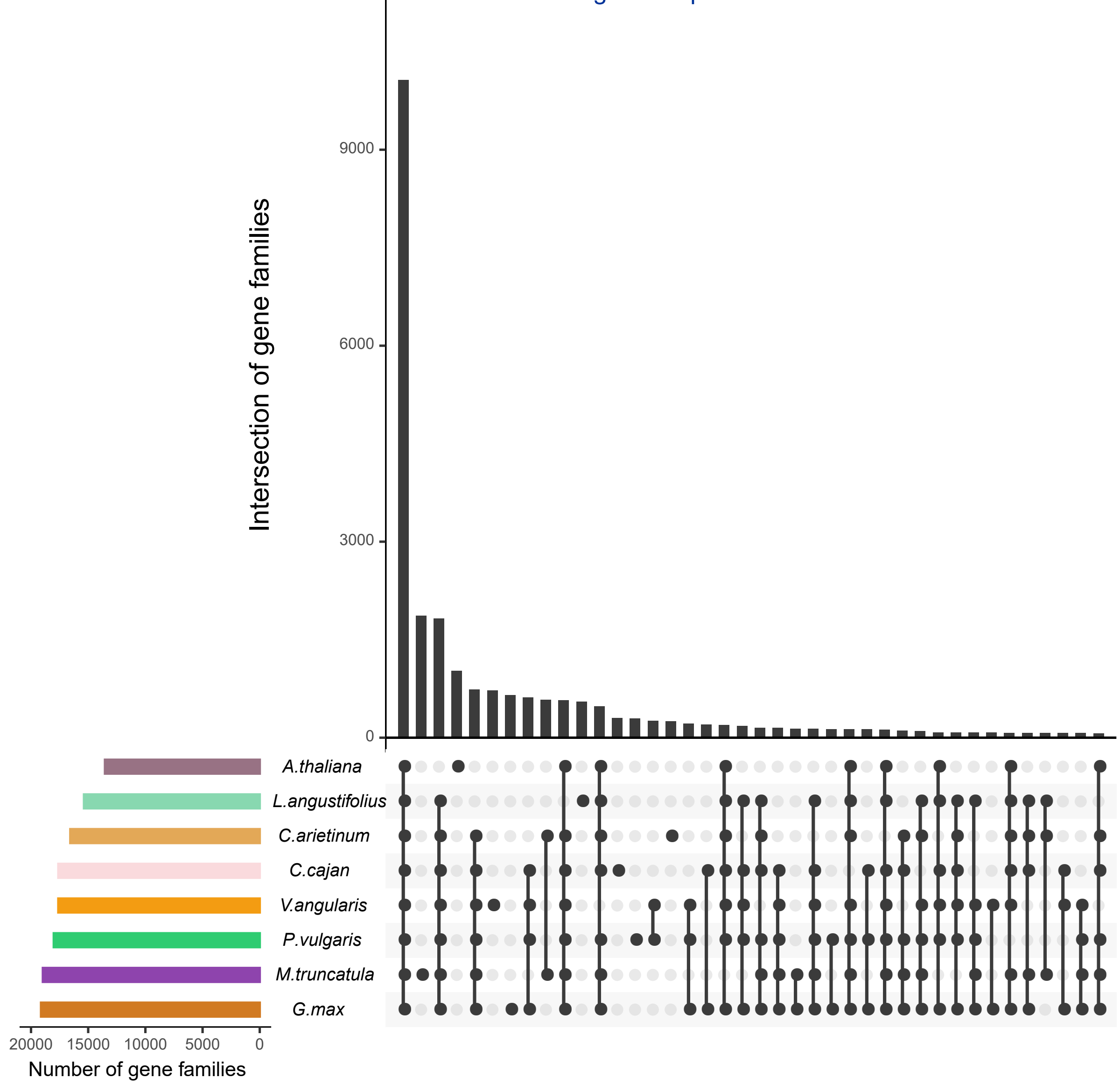

B

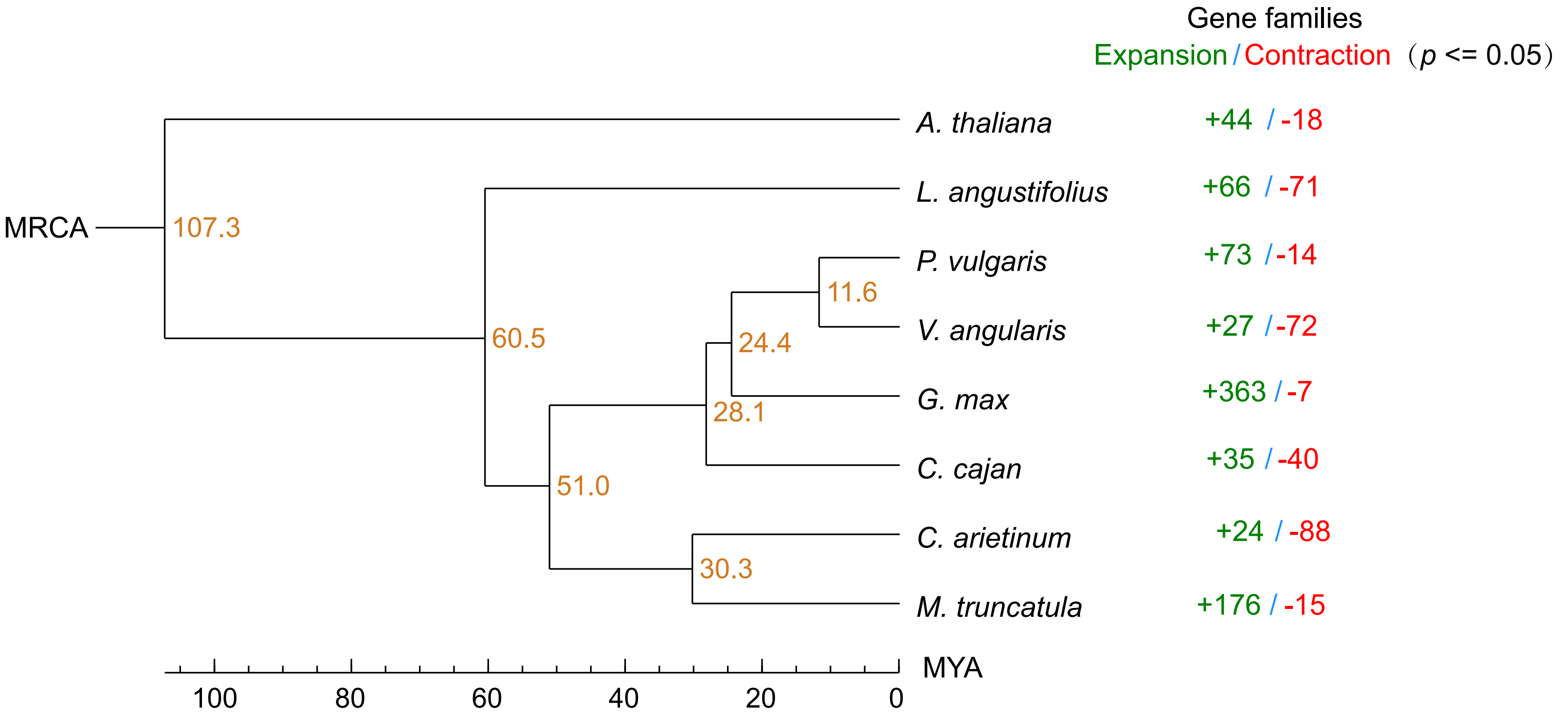

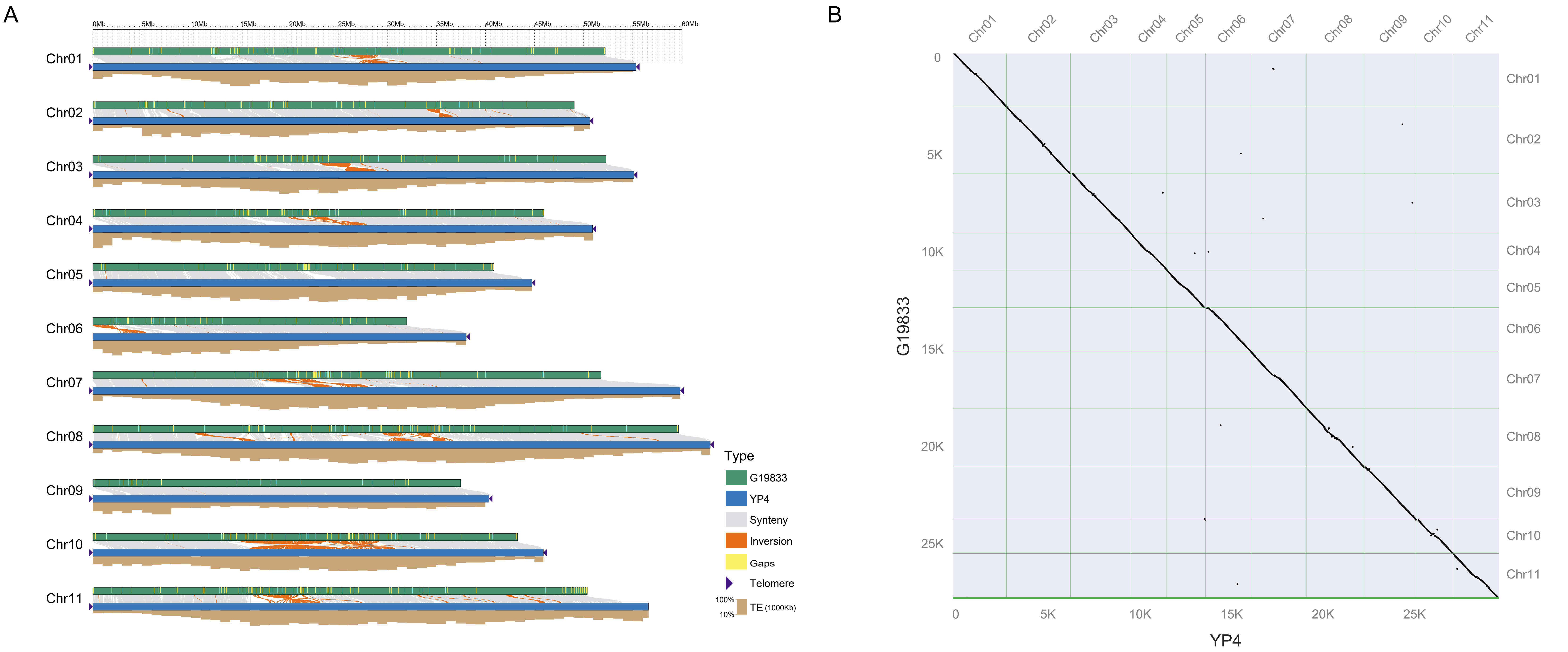
A
0Mb
5Mb
10Mb
15Mb
20Mb
25Mb
30Mb
35Mb
40Mb
45Mb
50Mb
55Mb
60Mb
Chr01
Chr02
Chr03
Chr04
Chr05
Chr06
Chr07
Chr08
Chr09
Chr10
Chr11
Type
G19833
YP4
Synteny
Inversion
Gaps
Telomere
100%
10%
TE (1000Kb)
B
Chr01
Chr02
Chr03
Chr04
Chr05
Chr06
Chr07
Chr08
Chr09
Chr10
Chr11
0
5K
10K
15K
20K
25K
G19833
YP4

Supplementary Figure

Click here to access/download
Supplementary Material
Supplementary Figure1-9.docx

Supplementary Table

Click here to access/download
Supplementary Material
Supplementary Table S1-S19.xlsx
