# Supplementary material for: Telomere-to-telomere genome of common bean (Phaseolus vulgaris L., YP4)
Source: Gigascience. 2025 May 14;14:giaf001. doi: 10.1093/gigascience/giaf001 (PMC12077395; doi:10.1093/gigascience/giaf001)
Supplement: giaf001_Response_to_Reviewer_Comments_Original_Submission [file giaf001_response_to_reviewer_comments_original_submission.pdf]

# Author's Response To Reviewer Comments

Dear editor,
We sincerely thank you and the reviewers for your valuable comments and instructive advice, which help us to improve and revise our manuscript (Manuscript Number: GIGA-D-24-00244, Title: Telomere-to-telomere gap-free genome of common bean (Phaseolus vulgaris L., YP4)). We have made detailed changes accordingly. Our point-by-point responses to the reviewers' comments are provided as follows for your consideration. For ease of reviewing, all the significant changes in the revised manuscript have been highlighted in red.
Reviewer reports:
Reviewer #1: This study presents a gap-free reference genome for the common bean. Although the data is good, it does not show much valuable biological insights. Maybe, the best application of the T2T assembly is for the study of centromere structure. However, the authors did not perform any analysis on the repeat unit in centromeres.
Answer: Thanks for your positive comments on our work. The manuscript, designed as a Data Note-type article, focuses on the analytical aspects of constructing the T2T genome rather than focusing on biological stories. It follows a format similar to the high-quality Chiridota heheva genome published in Gigascience (Pu, Zhou et al. 2024), prioritizing detailed analysis and data presentation.
Following your recommendations, we conducted an analysis of the centromere region, revealing conserved centromere function and 11 repeat unit sequences. Additionally, we explored YP4-specific genes to uncover valuable biological insights. Please find more details in lines 303-323 and 363-371 of the revised manuscript.

Reference：
Pu, Y., et al., A high-quality chromosomal genome assembly of the sea cucumber Chiridota heheva and its hydrothermal adaptation. GigaScience, 2024. 13.

Line 81: "It originating from" grammar error
Answer: Thank you for your reminder. We changed the sentence to "It originates from". Please find more details in lines 85 of the revised manuscript.

Line 234: "Notably, the contig N50 was 824-, and 2981-fold longer than earlier released genome versions" should list the two earlier version, respectively.
Answer: Thanks for your nice comment. These previously published genome versions are G19833 (Schmutz et al., 2014), BAT93 (Vlasova et al., 2016), and Flavert (recently published; Carrère, Mayjonade et al. 2023), and we provide detailed contig N50 values for these genomes. Please find more details in lines 250-253 of the revised manuscript.

Reference:
Carrère, S., et al., First whole genome assembly and annotation of a European common bean cultivar using PacBio HiFi and Iso-Seq data. Data in Brief, 2023. 48: p. 109182.
Schmutz, J., et al., A reference genome for common bean and genome-wide analysis of dual domestications. Nature Genetics, 2014. 46: p. 707 - 713.
Vlasova, A. et al. Genome and transcriptome analysis of the Mesoamerican common bean and the role of gene duplications in establishing tissue and temporal specialization of genes. in Genome biology Vol. 17 32 (2016).

Line 279: "Notably, 96.57% the RNA-seq reads" grammar error
Answer: Thank you for your reminder. We added the word "of" in the sentence. Please find more details in lines 297-298 of the revised manuscript.

Fig 3b seems strange. Does the whole pie should be cut into 3 parts? In addition, the legend "RNA-seq clean data verified the accuracy of protein-coding gene prediction" is not suitable.
Answer: Thanks for your comment. We referred to Fig 5b (see below) of the oriental armyworm genome

study (Xu et al., 2023) to create Fig 3b and its legend. The pie chart in our figure comprises three segments: Exon, Intron, and Intergenic, displayed in a 3D stereoscopic format. Our primary objective is to showcase the precision of gene set prediction, underlined by the substantial proportion of transcriptome reads found in the exon region (96.57%). A similar figure was also created in the Astragalus membranaceus genome study (Fan et al., 2024).

Reference:

Fan, H., et al., Chromosome-scale genome assembly of Astragalus membranaceus using PacBio and Hi-C technologies. Scientific Data, 2024. 11(1): p. 1071.
Xu, C., et al. Chromosome level genome assembly of oriental armyworm Mythimna separata. Scientific data, 2023. 10, 597

There are lots of grammar error needs corrected. It is better to use some AI or ask a native speaker to polish the language.
Answer: Thanks for your comment. In the revised version, we initially utilized ChatGPT to improve the writing quality. Following that, we sought guidance from seasoned professors with extensive writing proficiency. We sincerely hope that these modifications comply with the publishing guidelines.

Reviewer #2: The manuscript by Wang et al., describes a telomere-to-telomere sequence assembly of common bean (P. vulgaris) and a basic annotation. Two high quality genome assemblies of common bean have been published before. One (Schmutz et al., 2014) has been mentioned, another (Carrere et al., 2023) has not been mentioned in the manuscript. The second assembly was based on SMRT reads and reported a 566.23 Mb-long chromosome scale assembly with 29,549 annotated genes and BUSCO completeness of 99.2%.
Answer: Thanks for your nice comment. In the revised article, the background section has been enhanced to incorporate the description of the common bean genome published in 2023 by Carrère, Mayjonade et al. (Carrère, Mayjonade et al. 2023). Key indicators related to this study have been summarized in Table 1. Furthermore, a recent report on the pan-genome research of the common bean by Cortinovis, Vincenzi et al. in 2024 (Cortinovis, Vincenzi et al. 2024) has been included in the background section. These updates contribute to a more comprehensive understanding of the current research landscape on the common bean genome. For further details, please refer to lines 60-62 of the revised manuscript.

Reference:

Carrère, S., et al., First whole genome assembly and annotation of a European common bean cultivar using PacBio HiFi and Iso-Seq data. Data in Brief, 2023. 48: p. 109182.
Cortinovis, G., et al., Adaptive gene loss in the common bean pan-genome during range expansion and domestication. Nature Communications, 2024. 15(1): p. 6698.

The present assembly claims, it is gap-free. Actually, claiming gap-free is a big ask. It is difficult to prove. Telomere-to-telomere is a correct terminology. The present manuscript does not report any new scientific information.
Answer: Thank you for your reminder. The primary challenge in genome assembly is not the genome size, but the presence of repetitive sequences. In plants, centromere regions are rich in retrotransposons and tandem repeats, while telomeres consist of highly conserved tandem nucleotide repeats. In this study, we employed the latest sequencing technology and assembly algorithms to assemble the YP4 genome, successfully capturing all centromeres. Proving the quality of gap-free assemblies is difficult. Methods such as fluorescence in situ hybridization (FISH) and chromatin immunoprecipitation followed by high-throughput sequencing (ChIP-seq) are essential for validating the intricate genomic architectures and repetitive regions. These techniques will inform our future research efforts. To enhance the rigor of the article, we have removed the term "gap-free" in the revised manuscript.
The manuscript, intended for a Data Note-type article, emphasizes the analytical aspects of constructing the T2T genome for further study rather than focusing on biological stories. Its structure closely resembles

that of the high-quality Chiridota heheva genome recently published in Gigascience (Pu, Zhou et al. 2024), prioritizing detailed analysis and data presentation.
To enhance the significance of the data, we identified genes within the centromeric regions and conducted GO functional enrichment analysis. The most significant GO term we discovered was "nucleic acid binding," a finding that aligns with similar observations in rice gap-free studies (Song et al., 2021). For further details, please refer to lines 303-323 of the revised manuscript.

Reference：

Pu, Y., et al., A high-quality chromosomal genome assembly of the sea cucumber Chiridota heheva and its hydrothermal adaptation. GigaScience, 2024. 13.
Song, J.-M., et al., Two gap-free reference genomes and a global view of the centromere architecture in rice. Molecular Plant, 2021. 14(10): p. 1757-1767.

Had they identified any gene or locus associated with an important trait in this assembly which has been absent in the previously published assemblies, then they could have argued the importance and better utility of these assembly. At present form of
the manuscript, it is just another high-quality assembly of common bean. The other analyses presented in the manuscript are routine and do not provide new insight.
Answer: Thanks for your nice comment. This study aims to provide the first T2T version of the common bean genome, enabling researchers to perform diverse omics analyses, including selection signal analysis of the centromere region, structural variation analysis, and pan-genomic studies. Additionally, we believe the new insights from our revised manuscript are as follows:
(1)We have added the characteristics of centromeres, discovering conserved centromere functions and identifying 11 repeat unit sequences.
(2)We conducted a comparative analysis of YP4-specific genes against G19833, indicating that YP4 may exhibit enhanced resistance and a more intricate regulatory network.

For further details, please refer to lines 303-323 and 363-371 of the revised manuscript.

With best regards,
Yours sincerely
